# Phylogenomic Barcoding of Soil Seed Bank–Persistent and Wind‐Dispersed Non‐Native Plant Species in South Georgia

**DOI:** 10.1111/1755-0998.70068

**Published:** 2025-11-08

**Authors:** Juan Viruel, Calum J. Sweeney, Rachel Day, Kaitalin White, Wayne Dawson, Bradley Myer, Kelvin Floyd, Marcella Corcoran, Carey Kelting, Sally Poncet, Félix Forest, Colin Clubbe, Rosemary J. Newton

**Affiliations:** ^1^ Technological College University of Zaragoza, Crta. Cuarte sn Huesca Spain; ^2^ Royal Botanic Gardens Kew, Richmond UK; ^3^ National Parks and Wildlife Service Killarney Ireland; ^4^ Department of Biosciences Durham University Durham UK; ^5^ Department of Evolution, Ecology and Behaviour, Institute of Infection, Veterinary and Ecological Sciences University of Liverpool Liverpool UK; ^6^ Indigena Biosecurity International Nelson New Zealand

**Keywords:** angiosperms353, genome skimming, high‐throughput sequencing, hyb‐seq, invasive, phylogenetics, seedlings identification, target capture sequencing

## Abstract

Climate change and invasive species are leading drivers of biodiversity loss, with island ecosystems being especially vulnerable. South Georgia, a remote sub‐Antarctic island, is 170 km long with approximately 30,000 ha of vegetated coastal areas, as snow and ice dominate the inland regions. Human activities on the island have historically introduced non‐native species, resulting in 41 introduced vascular plant species compared with only 24 native ones. To address this imbalance, the South Georgia Non‐Native Plant Management Strategy was implemented (2016–2020) to control non‐native plant populations. We assessed emergent seedlings from South Georgia soil samples and wind‐dispersed seeds to determine which species persist in the soil seed bank and contribute to dispersal. Using a molecular barcoding approach, we evaluated traditional markers (*rbc*L and *mat*K) and optimized a high‐throughput Angiosperms353 sequencing pipeline for accurate seedling identification. We generated a reference library covering all native and non‐native species and applied this to 1,498 emergent seedlings and 737 trapped seeds. Molecular barcoding identified 21 species, including 10 non‐natives and 11 natives. Strikingly, 84% of emergent seedlings were non‐native, with Class III invasive species ( *Cerastium fontanum* , *Poa annua* , *Taraxacum officinale* ) dominating across most sites and in all wind traps. By contrast, Class I and II species occurred rarely and only at a few sites, indicating that management efforts have substantially reduced their spread, though viable seeds persist in the soil. These findings highlight both the continued threat from persistent seed banks of dominant invaders and the value of molecular barcoding for long‐term monitoring. Our approach provides a framework for biosecurity and restoration management in South Georgia and other vulnerable ecosystems under climate change pressures.

## 1 Introduction

Climate change and invasive species represent two of the leading drivers of biodiversity loss globally (Nelson 2005; Mainka and Howard 2010; Hald‐Mortensen 2023; IPBES 2023). Invasive species can be highly competitive in diverse ecosystems, supported by traits such as a wide range of germination and growth conditions, high reproductive capacity, and efficient dispersal mechanisms (Grime et al. 2007; Mainka and Howard 2010; Huebner 2022; Gioria et al. 2023). These characteristics make them a significant conservation concern worldwide, threatening native biodiversity and incurring high economic costs (Keller et al. 2017; Robuchon et al. 2025). Additionally, the interactions between climate change and invasive species can create synergistic effects, where invasive species may gain further competitive advantages in disturbed ecosystems under climate warming scenarios, thus intensifying their impact on ecosystem resilience and native species survival (Mainka and Howard 2010; Gioria et al. 2023; Colberg et al. 2024). This phenomenon has been observed globally, with certain non‐native species spreading rapidly in regions where warming temperatures reduce ecological barriers, for example frost, which can restrict seedling establishment (Hellmann et al. 2008; Sorte et al. 2013; Guiden and Roca 2025). Concurrently, climate change scenarios such as the Shared Socioeconomic Pathways (SSP) highlight potential futures, ranging from environmentally conscious pathways to more detrimental ones marked by inequality and regional rivalry (Riahi et al. 2017; Meinshausen et al. 2020). Climate change has already profoundly impacted ecosystems globally, with regions like the sub‐Antarctic experiencing accelerated warming (Anisimov et al. 2007; Colesie et al. 2022; Werner et al. 2023; van der Merwe et al. 2024). Island ecosystems are especially susceptible to climate change due to limited habitable space, small population sizes, and low functional redundancy, resulting in a disproportionate loss of biodiversity (Wong et al. 2005; Harter et al. 2015; Ferreira et al. 2019; Macinnis‐Ng et al. 2021; Lee et al. 2022). Non‐native species introduced into new environments may become invasive if conditions enable them to outcompete native species. This threat is expected to intensify under climate warming as invasive species show increased resilience and adaptability to broader environmental conditions (Cretaz and Kelty 1999; Colautti and MacIsaac 2004; Grime et al. 2007; Mainka and Howard 2010; Finch et al. 2021; Osland et al. 2023; Bradley et al. 2024; Guiden and Roca 2025).

The remote sub‐Antarctic island of South Georgia, approximately 170 km in length and encompassing around 30,000 ha of vegetated coastal areas, supports a mosaic of six habitat types. The littoral zone is characterised by plants tolerant to sea spray and salt, while coastal flats and slopes are nutrient‐rich and dominated by tussock grass ( *Poa flabellata* (Lam.) Raspail), a native species forming dense vegetative plumes, described by Captain Cook in 1775 (Hall 2007; Galbraith et al. 2011; Groff et al. 2020). Inland hillsides and valley slopes are generally sheltered and covered by grasses such as *Festuca contracta* Kirk, whereas seepage slopes and valley floors are often waterlogged and dominated by rushes. Glacial deposits, scree, and inland rock are windswept with little soil, supporting mainly mosses and lichens, and sea cliffs are largely exposed and mostly devoid of vascular plants (Galbraith et al. 2011).

South Georgia holds significant conservation value as a biodiversity hotspot within the Southern Ocean, with its marine shelf among the most species‐rich regions documented in this part of the world (Hogg et al. 2011; Trathan 2023). Largely uninhabited, the island is recognised as one of the world's most important wilderness areas, with intact ecosystems and unique flora and fauna adapted to its sub‐Antarctic conditions. It also served as a glacial refugium, allowing certain species to persist through past climatic extremes (Aguado‐Lara et al. 2025). Protecting its biodiversity is therefore critical for maintaining genetic diversity and ecological resilience in one of the planet's most remote and fragile ecosystems. However, human occupation since the early 20th century, particularly intensive whaling between 1904 and 1965, led to the introduction of numerous non‐native species (Moore et al. 1999; Galbraith et al. 2011; Upson et al. 2017). The eradication of reindeer and rodents has reduced grazing pressure, potentially promoting the spread of introduced plants (Cook et al. 2010; GSGSSI 2016, 2022), while glacier retreat continues to open new ground for plant colonisation (Tichit et al. 2024). As a result, introduced flora outnumbered native species in both richness and extent, with major concentrations on the Lewin, Thatcher, and Barff Peninsulas (Figure S1; Floyd 2019).

The South Georgia Non‐Native Plant Management Strategy (2016–2020), led by the Government of South Georgia and the South Sandwich Islands (GSGSSI), classified non‐native flora into three management classes: Class I (species with limited populations), Class II (species potentially detrimental to native biodiversity), and Class III (widely dispersed species difficult to control) (GSGSSI 2016, 2022). This initiative aimed to fully eradicate 75% of Class I species and reduce the distribution of Class II and Class III species to limit spread and support native flora conservation. These management efforts build on previous eradication programmes for invasive vertebrates (such as the declaration of rat‐free status in 2018; https://sght.org/news/south‐georgia‐declared‐rodent‐free/; Martin 2015), demonstrating South Georgia's long‐term commitment to ecological restoration. These prior efforts have set a solid foundation for developing sophisticated plant eradication strategies, providing a model for invasive species management in other remote ecosystems (McIntosh and Walton 2000). However, assessing the success of this programme is challenging, especially regarding Class I and II species that may remain dormant in the soil seed bank. As restoration and management efforts intensify, the ability to accurately detect and identify non‐native plant species, particularly at early life stages, remains challenging yet important. Morphological identification of seedlings is often unreliable due to a lack of diagnostic traits for young seedlings, highlighting the need for molecular identification alternatives (Galbraith et al. 2011).

Molecular barcoding, which identifies species based on selected genetic markers, offers a reliable alternative when morphological identification is not possible (Abdi et al. 2024), especially for young seedlings. This technique relies on comparing sequences from unidentified samples to a curated database of verified plant sequences (Chac and Thinh 2023). Traditional barcoding has employed the plastid regions *rbc*L and *mat*K due to their relatively high universality across plant taxa (CBOL 2009; Kress and Erickson 2012; Letsiou et al. 2024). Due to the scarcity of data for species occurring throughout sub‐Antarctic islands, recent efforts have focused on increasing the representativeness of sequence data from species native to islands affected by invasive species, such as on Marion Island (Chau et al. 2020), where traditional markers also showed high discriminatory power and success rates in a flora with such a low and phylogenetically divergent diversity. While these markers sometimes fail to differentiate closely related species (Sahin et al. 2025), such as those within the Poaceae family (Birch et al. 2017), this highlights that *rbc*L and *mat*K, despite their limitations, can still be very effective for molecular barcoding in simplified island communities. To overcome their limitations more generally, advances in phylogenomic methods and high‐throughput sequencing (HTS) now enable in‐depth sequencing across many loci, significantly improving taxonomic resolution for non‐model plants, especially in conservation contexts where precise identification is critical (Escudero et al. 2020; Baker et al. 2022).

In this study, we evaluated the success of the invasive plant eradication programme in South Georgia by quantifying the presence of invasive species seeds in the soil seed bank and dispersed by wind. Specifically, we: (i) assessed the resolution of traditional barcoding markers (*rbc*L and *mat*K) for South Georgia's flora; (ii) developed an optimised HTS‐based barcoding method to distinguish between native and non‐native plant species; and (iii) evaluated the longer‐term persistence of species targeted for eradication by identifying seedlings emerging from soil samples and dispersed by wind across key areas on the island.

## 2 Materials and Methods

**FIGURE 1:**
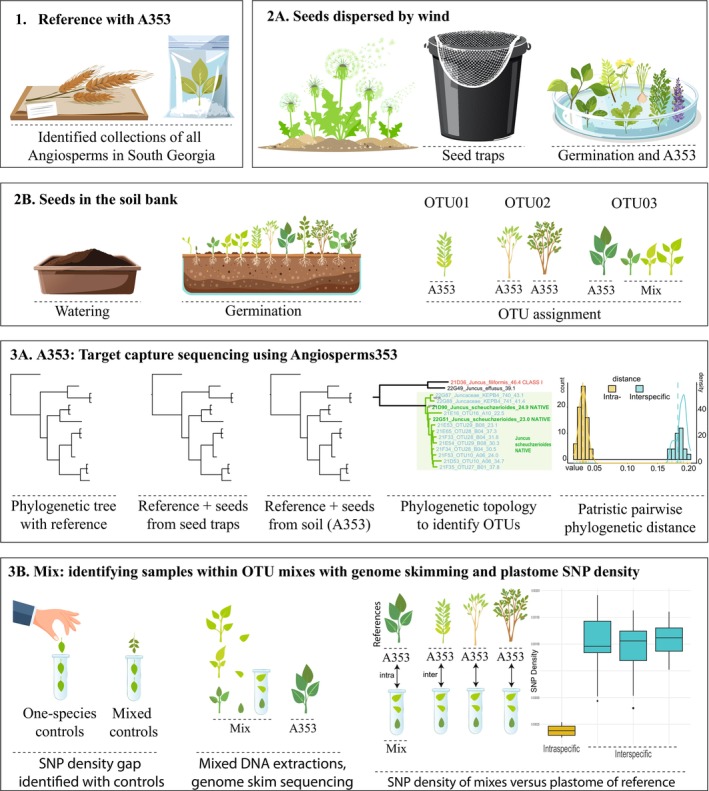
Experimental design framework to identify seedlings from South Georgia using molecular barcoding: (1) producing a reference library with Angiosperms353; (2A) sampling seeds collected by wind, (2B) sampling seedlings emerging from soil samples, (3A) sequencing individual samples with Angiosperms353 to identify seedlings using phylogenetic topology and patristic pairwise phylogenetic distances, (3B) identifying samples within OTU mixes with genome skimming and plastome SNP density. A353, target capture sequencing using the Angiosperms bait set kit. OTU, operational taxonomic unit.

A framework summarising the methodologies herein presented and experimental design is visualised in Figure 1.

### 2.1 Seed and Soil Sampling and Plant Material

Sampling sites were selected to maximise the likelihood of detecting non‐native propagules, either within the soil seed bank or among wind‐dispersed seeds (Figure S1). Sites were chosen based on the presence of Class I and Class II invasive species that still require management, or because such species had been subject to control efforts at these locations in the recent past.

#### 2.1.1 Soil Sampling

Soil samples were collected from 20 invaded sites, with five replicates per site, between 1 January and 3 February 2019 (Figure S1). At each sampling point, soil was removed from a 7 × 7 cm area to a depth of 5 cm, yielding approximately 200 cm^3^ per sample. Prior to sampling, any above‐ground vegetation was clipped back as close to the soil surface as possible and discarded. Soil samples were placed in labelled polyethylene Ziploc bags, and the trowel was cleaned between sites to prevent cross‐contamination. Bags were opened to allow samples to air dry before transport to the Millennium Seed Bank (Royal Botanic Gardens, Kew, Wakehurst, UK), where germination experiments were conducted in quarantine conditions (under Department for Environment, Food and Rural Affairs (Defra) Soil Permit 101466‐398618‐0).

Germination tests were set up in 180 mm (L) × 140mm (W) × 45mm (D) foil containers with drainage holes punched into the base. Into each tray, 200 cm^3^ of an autoclaved 2:1 Melcourt potting soil: perlite mix with 25 g of osmocote per 4 L was added, overlaid with 100 cm^3^ of autoclaved sand. Germination containers were saturated overnight in trays of deionised water before the addition of 80 cm^3^ of each South Georgia soil sample. Samples were moistened with a fine spray under a designated quarantine hood, sealed in Ziploc bags, and incubated at 25°C/10°C (12 h/12 h day–night). Germination temperatures were chosen following examination of Millennium Seed Bank routine seed germination data for both non‐native and native South Georgia plant species (results not shown) to likely be the best temperature regime to promote germination in most species, rather than directly replicating field conditions on the island. Trays were checked weekly for at least 60 days, and leaf and stem tissue from emergent seedlings was harvested and dried in silica gel. Trays were discarded after 60 days if no new seedling emergence had been observed for at least 14 days.

Each seedling was assigned to an operational taxonomic unit based on morphology. For Angiosperms353 sequencing, we targeted all OTUs/site combinations to ensure representative coverage. All Poaceae seedlings were sequenced individually due to the difficulty of resolving closely related taxa in this family. For the remaining seedlings belonging to the same OTU/site, we employed a pooled genome skimming approach, a high‐throughput approach that extracts genomic information from total DNA without enrichment steps (Dodsworth 2015), to validate their identity (see below for details).

#### 2.1.2 Wind Sampling

Seed traps were deployed at six sites representing three habitat types: two invaded sites, two native sites, and two sites recently exposed by retreating glaciers. At each site, five bucket traps (modified after Morris et al. 2011) were installed in January 2019, each oriented in a different direction to capture seeds from all wind exposures. Traps were left in the field for a period of 40–60 days before retrieval.

Seeds were transported to the Millennium Seed Bank, sown on 1% agar, and incubated at 25°C 12 h/day and 10°C 12 h/night for at least 7 weeks, with regular monitoring. Germinated seedlings were transferred into pots containing compost and grown until large enough to harvest, after which leaf and stem tissue was dried in silica gel for DNA analysis. Ungerminated seeds were dissected longitudinally with fine forceps and a scalpel under a light microscope to assess whether they were empty. Ungerminated seeds with contents were tested for viability using a tetrazolium test (Leist et al. 2003; Miller 2010).

### 2.2 Evaluation of Traditional Barcoding Regions to Identify Plant Species in South Georgia

All the available *rbc*L and *mat*K sequence data for the 57 angiosperm species known to occur in South Georgia were obtained from the GenBank database (National Center for Biotechnology Information, NCBI), as well as *Persea americana* used as an outgroup taxon for the phylogenetic analyses. Species belonging to the same genus were selected when sequence data were not available for any of the South Georgian species. The plastid markers *rbc*L and *mat*K were selected due to the volume of sequences available and their established track record as barcoding regions (CBOL 2009). When data were not available for a taxon, sequences from a congeneric species were retrieved (hereafter “placeholders”). Sequences were cleaned, trimmed, and aligned using MUSCLE and default settings in the software MEGA7 (Kumar et al. 2016). Identical haplotypes were identified using the Fabox DNACollapser tool to remove duplicate data (available at https://users‐birc.au.dk/palle/php/fabox/dnacollapser.php). Phylogenetic analyses as well as pairwise genetic distance from alignments were used to assess the barcoding potential for species‐level identification. Both maximum likelihood (RAxML v.8; Stamatakis 2014) and Bayesian inference (MrBayes 3.2; Ronquist et al. 2012) methods were employed to construct molecular phylogenetic trees. The best fit model was selected using the corrected Akaike Information Criterion implemented in jModelTest 2 (Darriba et al. 2012) via the CIPRES servers (Miller et al. 2010), which was GTR + I + G for both *rbc*L and *mat*K. RAxML analyses were performed on CIPRES (Miller et al. 2010) using default settings and 1000 bootstrap replicates. MrBayes analyses were performed using Markov chain Monte Carlo sampling running for two million generations with sampling occurring every 1000 generations. A heating temperature of 0.2 was used. The first 25% of trees were specified as burn‐in and discarded before computing a majority‐rule consensus tree. Pairwise intraspecific genetic distances were compared to interspecific distances to assess the discrimination resolution of *rbc*L and *mat*K as barcoding regions using the R package BarcodingR (Zhang et al. 2020). Genetic distances were calculated using the TN93 model allowing for variation in rates of transversions and transitions (Tamura and Nei 1993), and then heatmaps were produced using the R package Ape (Paradis and Schliep 2019). Successful identification was determined based on the “liberal” tree‐based method applied by Meier et al. (2006), a revised version of Hebert et al. (2003) which extends beyond sequence clustering as criteria for unambiguous identification.

### 2.3 Generation of a Reference DNA Sequence Library Using Angiosperms353 to Enable Molecular Barcoding for the South Georgia Flora

DNA was extracted using a CTAB‐based protocol (Doyle and Doyle 1987) for samples of at least one specimen for all native and introduced angiosperm species in South Georgia (Table 1). We constructed genomic libraries as optimized in Viruel et al. (2019) for half volumes with the NEBNext Ultra II DNA Library Prep Kit for Illumina (New England Biolabs, Ipswich, Massachusetts, USA), AMPure XP magnetic beads, and NEBNext Multiplex Oligos for Illumina (Dual Index Primer Sets I and II) as tags for simultaneous sequencing. Pools containing twelve genomic libraries were mixed in equimolar conditions and enriched using half reactions of the Angiosperms353 probe kit (Johnson et al. 2019) as detailed in the myBaits kit manual v3.02 (Arbor Biosciences). DNA concentration and library fragment size were calculated using a QuantusTM fluorometer (Promega Corp.) and an Agilent 4200 TapeStation (Agilent Technologies, Santa Clara, CA, United States), respectively. Sequencing was performed on a HiSeq (Illumina Inc.) by Macrogen (Seoul, South Korea), which produced 2 × 150 bp paired‐end reads. Sequence data are available at the European Nucleotide Archive repository: project PRJEB88856.

**TABLE 1:** List of plant species known to occur in South Georgia, including common name, dispersal strategy, and their native or introduced status. Introduced species were classified according to distribution extent, invasiveness, and ease of control. The aim of the South Georgia Non‐Native Plant Management Strategy 2016–2020 was to eradicate at least 75% of Class I species, reduce the distribution and abundance of Class II species, and reduce selected populations of Class III species to reduce further spread into uninvaded sites.

Species name	Author	Common name	Family	Dispersal unit	Intro or native	Class
*Acaena magellanica*	(Lam.) Vahl	Greater burnet	Rosaceae	Seed	Native	
*Acaena tenera*	Albov	Lesser burnet	Rosaceae	Seed	Native	
*Achillea millefolium*	L.	Yarrow	Asteraceae	Fruit (single seed)	Introduced	I
*Achillea ptarmica*	L.	Sneezewort	Asteraceae	Fruit (single seed)	Introduced	I
*Agrostis capillaris*	L.	Common bent	Poaceae	Fruit/Floret	Introduced	II
*Agrostis vinealis*	Schreb.	Brown bent	Poaceae	Fruit/Floret	Introduced	I
*Alopecurus magellanicus*	Lam.	Antarctic foxtail	Poaceae	Fruit/Floret	Native	
*Anthoxanthum odoratum*	L.	Sweet vernal grass	Poaceae	Fruit/Floret	Introduced	I
*Anthriscus sylvestris*	(L.) Hoffm.	Cow parsley	Apiaceae	Fruit	Introduced	I
*Avenella flexuosa*	(L.) Drejer	Wavy hairgrass	Poaceae	Fruit/Floret	Introduced	I
*Callitriche antarctica*	Engelm. ex Hegelm.	Antarctic starwort	Plantaginaceae	Fruit (single seed)	Native	
*Capsella bursa‐pastoris*	(L.) Medik.	Shepherd's purse	Brassicaceae	Seeds (from pod)	Introduced	I
*Cardamine glacialis*	(G.Forst.) DC.	Bittercress	Brassicaceae	Seeds (from pod)	Introduced	I
*Carex aquatilis*	Wahlenb.	Water sedge	Cyperaceae	Fruit (utricle)	Introduced	I
*Carex meridensis*	(Steyerm.) J.R.Starr	Smith's sedge	Cyperaceae	Fruit (utricle)	Native	
*Carex nigra*	(L.) Reichard	Common sedge	Cyperaceae	Fruit (utricle)	Introduced	I
*Carex vallis‐pulchrae*	Phil.	Marsh sedge	Cyperaceae	Fruit (utricle)	Introduced	I
*Cerastium fontanum*	Baumg.	Common mouse‐ear	Caryophyllaceae	Seeds (from capsule)	Introduced	III
*Colobanthus quitensis*	(Kunth) Bartl.	Antarctic pearlwort	Caryophyllaceae	Seeds	Native	
*Colobanthus subulatus*	(d'Urv.) Hook.f.	Lesser pearlwort	Caryophyllaceae	Seeds	Native	
*Dactylis glomerata*	L.	Cocksfoot	Poaceae	Fruit/Floret	Introduced	I
*Deschampsia antarctica*	É. Desv.	Antarctic hair‐grass	Poaceae	Fruit/Floret	Native	
*Deschampsia cespitosa*	(L.) P.Beauv.	Tufted hair grass	Poaceae	Fruit/Floret	Introduced	I
*Deschampsia parvula*	(Hook.f.) É. Desv.	Punkgrass	Poaceae	Fruit/Floret	Introduced	II
*Elymus repens*	(L.) Gould	Couch grass	Poaceae	Fruit/Floret	Introduced	I
*Empetrum rubrum*	Vahl ex Willd.	Diddle‐dee	Ericaceae	Fruit (fleshy edible drupe)	Introduced	I
*Festuca contracta*	Kirk	Tufted fescue	Poaceae	Fruit/Floret	Native	
*Festuca rubra*	L.	Red fescue	Poaceae	Fruit/Floret	Introduced	I
*Galium antarticum*	Hook.f.	Antarctic bedstraw	Rubiaceae	Fruits (containing 3 seeds)	Native	
*Juncus filiformis*	L.	Thread rush	Juncaceae	Seeds	Introduced	I
*Juncus scheuchzerioides* ^a^	Gaudich.	Native rush	Juncaceae	Seeds	Native	
*Leptinella scariosa*	Cass.	Feathery buttonweed	Asteraceae	Fruit (single seed)	Introduced	I
*Lobelia oligophylla*	(Wedd.) Lammers	Berry lobelia	Campanulaceae	Fruit (berry with many seeds)	Introduced	I
*Luzula congesta* ^b^	(Thuill.) Lej.	Congested wood‐rush	Juncaceae	Seeds (3 per capsule)	Introduced	I
*Luzula multiflora* ^b^	(Ehrh.) Lej.	Heath wood‐rush	Juncaceae	Seeds (3 per capsule)	Introduced	I
*Montia fontana*	L.	Water blinks	Montiaceae	Seed	Native	
*Nardus stricta*	L.	Mat grass	Poaceae	Fruit/Floret	Introduced	I
*Phleum alpinum*	L.	Alpine cat's‐tail	Poaceae	Fruit/Floret	Native	
*Poa annua*	L.	Annual meadow grass	Poaceae	Fruit/Floret	Introduced	III
*Poa flabellata*	(Lam.) Raspail	Tussac grass	Poaceae	Fruit/Floret	Native	
*Poa pratensis*	L.	Smooth meadow‐grass	Poaceae	Fruit/Floret	Introduced	II
*Poa trivialis*	L.	Rough meadow‐grass	Poaceae	Fruit/Floret	Introduced	II
*Ranunculus acris*	L.	Meadow buttercup	Ranunculaceae	Fruit (achene with single seed)	Introduced	I
*Ranunculus biternatus*	Sm.	Antarctic buttercup	Ranunculaceae	Fruit (achene with single seed)	Native	
*Ranunculus repens*	L.	Creeping buttercup	Ranunculaceae	Fruit (achene with single seed)	Introduced	I
*Rostkovia magellanica*	(Lam.) Hook.f.	Brown (or short) rush	Juncaceae	Seed	Native	
*Rumex acetosella*	L.	Sheep's sorrel	Polygonaceae	Fruit (Dry)	Introduced	I
*Rumex crispus*	L.	Curled dock	Polygonaceae	Fruit (Dry)	Introduced	I
*Sagina procumbens*	L.	Procumbent pearlwort	Caryophyllaceae	Seeds (from capsule)	Introduced	I
*Scorzoneroides autumnalis*	(L.) Moench	Autumn hawkbit	Asteraceae	Fruit (single seed)	Introduced	I
*Stellaria media*	(L.) Vill.	Common chickweed	Caryophyllaceae	Seeds (from capsule)	Introduced	I
*Taraxacum officinale* agg.	Anon.	Dandelion	Asteraceae	Fruit (single seed)	Introduced	III
*Trifolium repens*	L.	White clover	Fabaceae	Seed	Introduced	I
*Tripleurospermum inodorum*	(L.) Sch.bip.	Scentless mayweed	Asteraceae	Fruit (single seed)	Introduced	I
*Trisetum spicatum*	(L.) K.Richt.	Spikey trisetum	Poaceae	Fruit/Floret	Introduced	II
*Vaccinium vitis‐idaea*	L.	Cowberry	Ericaceae	Fruit (berry)	Introduced	I
*Veronica serpyllifolia*	L.	Thyme leaved speedwell	Plantaginaceae	Seeds (from capsule)	Introduced	I

^a^ Included within *Juncus scheuchzerioides* is *Juncus inconspicuus*, which is no longer recognized as a different species.

^b^ Originally *Luzula multiflora* subsp. *congesta* but this is now recognized as a different species to *Luzula multiflora* .

Trimmomatic v.0.35 (Bolger et al. 2014) was used to discard low‐quality bases (TRAILING 30) and to trim adapter sequences (ILLUMINACLIP) based on the reports produced by FastQC v.0.11.7 (Andrews 2010). We then used HybPiper v.1.3.1 (Johnson et al. 2016) with the reference file mega353 (McLay et al. 2021) to retrieve nuclear genes from the Angiosperms353 kit, performing both the mapping of reads and the subsequent *de novo* assembly.

Phylogenomic methods were followed to reconstruct phylogenetic trees containing one representative per species occurring in South Georgia. Sixty samples were used to build the molecular barcoding reference for the South Georgian flora, including all known native (16) and introduced (41) angiosperms, one hybrid (*Acaena magellanica* × *A. tenera* ), and two plant species new to South Georgia and occurring in the Falkland Islands (*Gaultheria pumila*, *Juncus effusus* ). *Trimenia moorei* obtained from the Kew Tree of Life Explorer (Baker et al. (2022); https://www.ebi.ac.uk/ena/browser/view/ERR5006124) was used as the outgroup taxon. *Nardus stricta* was obtained from RNAseq data (ERR1744598). The DNA sequence matrix produced for each gene was aligned as implemented in MAFFT v.7 (Katoh et al. 2002) using the –auto parameter and subsequently debugged with trimAl v.1.4.1 (Capella‐Gutiérrez et al. 2009) using the–automated1 option. We followed a supermatrix approach, using the concatenated and partitioned alignment obtained with FASconCAT‐G (Kück and Longo 2014) and with analyses conducted with IQTREE (Nguyen et al. 2015), applying a GTR + GAMMA substitution model and running 10,000 bootstrap replicates.

### 2.4 Identification of Invasive Species in South Georgia From Seeds Dispersed by Wind and in the Soil Seed Bank

Seedlings were individually dried in silica gel and assigned to operational taxonomic units (OTUs) based on morphological attributes for sampling site and replicate for three of the five replicates from soil samples. The remaining seedlings from soil samples were identified visually after confirming the identification of species using molecular barcoding for the first three replicates. At least one representative of each OTU per site was selected for molecular barcoding identification (i.e., at least one sample sequenced using Angiosperms353). DNA isolation, target capture approaches, and sequence data generation were conducted using the same approach as described above for the generation of the reference library (see Section 2.3). We also used the same phylogenomic methods to reconstruct phylogenetic trees as mentioned above, including both the samples from the reference DNA library and the unidentified samples obtained from seedlings.

Assignment of OTUs to species was done based on two criteria: (1) the phylogenetic placement of the OTUs in the tree comprising both OTUs and samples forming the reference DNA library, and (2) phylogenetic distances between OTUs and the closest reference DNA library sample. Monophyletic clades formed by unidentified samples and reference DNA library samples will be considered the same taxon, on the basis of expecting intraspecific phylogenetic distances to be lower than interspecific phylogenetic distances. Pairwise phylogenetic distances were calculated using the patristic method (distTips function from adephylo R package; Jombart et al. 2010), and heatmaps (fviz_dist function in factoextra R package; Kassambara and Mundt 2020) and histograms with density plots (ggpubr R package; Kassambara and Mundt 2020) showing intra‐ and interspecific pairwise phylogenetic distances were built for each genus, creating subsets of pairwise distances using the function dist_subset in usedist R package (Bittinger 2020).

To assess if the remaining seedlings which were not sequenced individually belonged to only one OTU, we compared their plastome sequences and estimated SNP (single nucleotide polymorphism) density from genome skimming data. These seedlings were pooled using equal amounts of dry weight in groups ranging from 2‐11 individuals. DNA was extracted from these pools and genomic libraries were constructed and sent for sequencing following the same approach as above, without the Angiosperms353 enrichment step. We also prepared two types of control pools for optimizing our analysis: (i) four pools with mixed species at different proportions: (1) 1:1 *Taraxacum*:*Phleum* (sample ID 21M88), (2) 1:1 *Taraxacum*:*Acaena* (21M89), (3) replicate of 1:1 *Taraxacum*:*Acaena* (21M90), (4) 1:9 *Taraxacum*:*Poa* (21M91), and (ii) pools with samples from the same species: (1) *Acaena* (21M94), (2) *Taraxacum* (21M95), (3) *Phleum* (21M96). Plastomes were reconstructed from off‐target reads obtained from the sequencing of Angiosperms353 for each of the samples sequenced individually using GetOrganelle v.1.7 (Jin et al. 2020) and the parameters ‐R 20 ‐k 21,45,65,85,105 ‐F embplant_pt ‐t 5. Paired reads from the pooled samples were mapped against the plastome reference built for each of the samples sequenced individually with Angiosperms353 occurring in the same site using BWA (Burrows Wheeler Aligner) v.0.7 (Li and Durbin 2009), and SAM files sorted and converted to BAM using SAMTools v.1.13 (Danecek et al. 2021). We used nQuire (Weiß et al. 2018) to count the number of SNPs found when comparing the reads from the pooled samples against the plastome of each of the samples occurring on each site, and calculated SNP density by dividing the number of SNPs by the total length of the plastome reference sequence for each sample. In nQuire, we used the ‐f parameter to adjust the minimum allelic frequency that was filtered, obtaining SNPs filtered at 20% (by default in nQuire) and 10% (‐f 0.1). Pools of samples attributed to an OTU are expected to have lower SNP density when compared to the plastome sequence of the same OTU (i.e., species), and higher SNP density when compared to other species. We used our control pooled samples to assess the implications in SNP density when more than one species is mixed in a pool. The code used to calculate SNP density and generate density plots and boxplots is provided in Data S1.

## 3 Results

### 3.1 Assessment of the Resolution Obtained With Traditional Molecular Markers for Molecular Barcoding

A total of 810 sequences of *rbc*L were retrieved from GenBank with an aligned length of 552 bp, and 606 sequences of *mat*K with an aligned length of 747 bp. RAxML and MrBayes trees had congruent topologies and concurred with relationships outlined in APG IV (The Angiosperm Phylogeny Group 2016). The highest proportion of species which can be identified was reconstructed with *mat*K (81%, 39/48), as opposed to *rbc*L (70%, 34/48). Genetic distances between species were on average three times greater when using *mat*K (32.8% vs. 10.8% polymorphisms) (Figure S2). Three clades (7 species in total) exhibited identical haplotypes shared across different species when using *rbc*L (Figure S2). Thus, we will focus on the details of the *mat*K results only hereafter.

Strong bootstrap and posterior probability support values were obtained for clades corresponding with family delimitations, with the lowest support values found within Poaceae and Cyperaceae (Figure S2). Sequences of *mat*K were available for 10/16 native species, and all genera of Poaceae on South Georgia could be identified. However, delineating the native *Poa flabellata* from the introduced *P. annua* was not possible. All genera can be identified using *mat*K (Figure S2). There were three categories of species that could be identified: (1) Those that can be identified outright using *mat*K, (2) those for which only one sequence is available and identifiable based on genetic distance to the closest relative only, and (3) those identified using a placeholder. Sequence availability was a limiting factor, with nine species having only a single sequence of *mat*K available. Although samples of *Cardamine hirsuta* and *Rumex crispus* were polyphyletic, they could still be identified due to their low intra‐specific genetic distance to closely related species.

Although we initially evaluated *rbc*L and *mat*K, their resolution was insufficient to reliably discriminate several closely related groups (e.g., Poaceae). For this reason, we did not use traditional markers to identify any seedlings. All seedling identifications reported in this study were therefore performed exclusively using the Angiosperms353 target capture sequencing approach, which provided the required taxonomic resolution across all families (see below).

### 3.2 Reconstruction of the Tree of Life for the Entire South Georgian Flora

**FIGURE 2:**
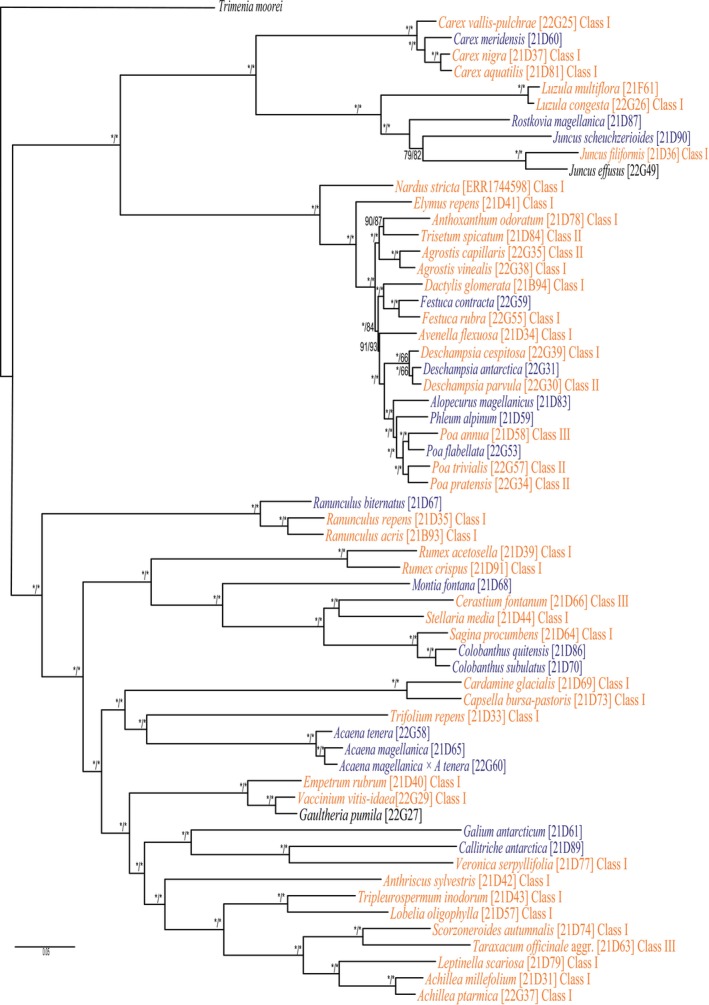
Phylogenetic tree with all angiosperms known to occur in South Georgia (in orange, non‐natives, in dark blue, natives) and relevant species occurring in neighbouring islands (in black) reconstructed using Angiosperms353 sequence data. Numbers on branches represent SH‐aLRT support (%)/ultrafast bootstrap support (%); asterisk * represents 100.

On average, 4,174,719 paired‐end reads were sequenced per sample (1,127,382–9,742,766) for Angiosperms353 data. The proportion of reads on target (i.e., enrichment efficiency) ranged from 6% to 49% (18% on average). The number of genes with sequences was 303 on average, although this value decreased to 232 when accounting for the number of genes with at least 50% of their total length. Gene recovery statistics for each of the samples in our reference are shown in Table 2. The phylogenetic tree reconstructed with our reference dataset showed remarkably high bootstrap support (i.e., 100) for all clades (Figure 2 and Figure S3).

**TABLE 2:** Target capture efficiency for the 60 samples used to reconstruct the phylogenetic tree for the entire flora of South Georgia, including 16 native species, 40 introduced species, one hybrid, and three additional plant species occurring in the Falkland Islands. ‘Paired‐end reads’ is the number of reads obtained after quality trimming with Trimmomatic. ‘Enrich. eff.’ is the proportion of reads on target. ‘Length %’ is the recovery length percentage calculated based on the nucleotides retrieved per sample relative to the full length of the reference target gene set (i.e., Angiosperms353 target capture kit).

Species name	Sample name	DNA ID	Collection ID	Location	Paired‐end reads	Enich. eff.	Assembled genes	Genes at 50%	Length %
*Acaena magellanica*	21D65	522861_7	Tissue Bank 16,158	SGSSI: South Georgia	7,291,542	0.26	341	301	83.3
*Acaena magellanica × A. tenera*	22G60	DNA_87	Ken Passfield 07/03/2022	King Edward Point. −54.280037, −36.497263	4,010,062	0.38	342	315	85.1
*Acaena tenera*	22G58	DNA_77	Ken Passfield 04/03/2022	Grytviken. −54.279307, −36.51128	5,704,547	0.35	340	316	88.0
*Achillea millefolium*	21D31	12692_35	Tissue Bank 15,705	France: Aquitaine: Dordogne	2,006,485	0.20	309	234	64.9
*Achillea ptarmica*	22G37	DNA_24	Sally Poncet 27/01/2022	Husvik. −54.1784, −36.713	1,414,621	0.28	335	308	79.4
*Agrostis capillaris*	22G35	DNA_26	Kelvin Floyd 28/01/2022	Grytviken. −54.286876, −36.502751	5,398,629	0.19	335	285	76.4
*Agrostis vinealis*	22G38	DNA_22	Sally Poncet 27/01/2022	Husvik. −54.1812, −36.7117	3,855,486	0.19	330	301	80.8
*Alopecurus magellanicus*	21D83	18,918	Tissue Bank 18,918	SGSSI: South Georgia	6,411,853	0.10	272	184	55.0
*Anthoxanthum odoratum*	21D78	522920_13	Tissue Bank 16,189	SGSSI: South Georgia	4,929,437	0.12	261	144	45.7
*Anthriscus sylvestris*	21D42	51884_1	Tissue Bank 15,858	Norway: Buskerud	1,890,260	0.14	280	146	45.9
*Avenella flexuosa*	21D34	26406_20	Tissue Bank 15,746	UK: England: West Sussex	6,335,292	0.07	264	165	51.2
*Callitriche antarctica*	21D89	18,901	Tissue Bank 18,901	SGSSI: South Georgia	1,277,891	0.15	282	179	50.3
*Capsella bursa‐pastoris*	21D73	591159_1	Tissue Bank 16,208	Italy	3,333,220	0.49	347	334	89.6
*Cardamine glacialis*	21D69	510718_2	Tissue Bank 16,033	SGSSI: South Georgia	3,728,520	0.39	348	339	92.5
*Carex aquatilis*	21D81	39,385	Tissue Bank 39,385	SGSSI: South Georgia	2,842,855	0.15	297	193	57.2
*Carex meridensis*	21D60	521598_8	Tissue Bank 16,106	SGSSI: South Georgia	8,938,614	0.16	304	181	52.2
*Carex nigra*	21D37	70971_14	Tissue Bank 15,907	UK: England: Greater London	3,549,684	0.16	304	211	59.6
*Carex vallis‐pulchrae*	22G25	DNA_01	Sally Poncet 16/01/2022	Hansen Valley. −54.1349, −36.695	2,354,756	0.13	297	213	60.6
*Cerastium fontanum*	21D66	521277_3	Tissue Bank 16,059	SGSSI: South Georgia	1,573,683	0.17	297	189	54.6
*Colobanthus quitensis*	21D86	39,246	Tissue Bank 39,246	SGSSI: South Georgia	1,484,494	0.22	312	220	59.3
*Colobanthus subulatus*	21D70	510682_3	Tissue Bank 16,031	SGSSI: South Georgia	2,093,029	0.17	305	210	58.5
*Dactylis glomerata*	21B94	44149_25	Tissue Bank 15,812	France: Pays de la Loire	6,614,265	0.10	319	269	72.7
*Deschampsia antarctica*	22G31	DNA_17	Sally Poncet 26/01/2022	Husvik. −54.1762, −36.7131	2,993,162	0.14	311	272	73.9
*Deschampsia cespitosa*	22G39	DNA_21	Sally Poncet 27/01/2022	Husvik. −54.1788, −36.7133	4,253,728	0.17	328	288	74.6
*Deschampsia parvula*	22G30	DNA_18	Sally Poncet 26/01/2022	Husvik. −54.1762, −36.7131	3,320,997	0.13	311	275	72.6
*Elymus repens*	21D41	482,510_10	Tissue Bank 482,510	SGSSI: South Georgia	8,232,640	0.15	213	97	35.3
*Empetrum rubrum*	21D40	505273_1	Tissue Bank 16,012	Falkland Is.: West Falkland	3,695,368	0.13	348	309	81.9
*Festuca contracta*	22G59	DNA_72	Ken Passfield 04/03/2022	Grytviken. −54.28046, −36.508501	2,720,418	0.09	263	202	57.8
*Festuca rubra*	22G55	DNA_66	Ken Passfield 26/02/2022	Ocean Harbour. −54.343781, −36.269474	3,981,426	0.12	309	256	69.6
*Galium antarticum*	21D61	52155_2	Tissue Bank 16,098	SGSSI: South Georgia	3,504,424	0.15	330	286	75.4
*Gaultheria pumila*	22G27	DNA_04	Sally Poncet 17/01/2022	Huselva. −54.1866, −36.7208	3,073,251	0.39	350	333	87.1
*Juncus effusus*	22G49	DNA_33	Kelvin Floyd 29/01/2022	Maiviken. −54.26184, −36.497569	5,740,634	0.08	241	133	39.1
*Juncus filiformis*	21D36	132013_10	Tissue Bank 15,917	UK: England: Durham	9,742,766	0.20	284	162	46.4
*Juncus scheuchzerioides*	21D90	39,220	Tissue Bank 39,220	SGSSI: South Georgia	1,650,324	0.14	191	78	24.9
*Leptinella scariosa*	21D79	662156_4	Tissue Bank 16,294	Falkland Is.: East Falkland	1,127,382	0.20	296	192	55.9
*Lobelia oligophylla*	21D57	510671_6	Tissue Bank 16,027	SGSSI: South Georgia	4,684,680	0.24	341	261	72.7
*Luzula congesta*	22G26	DNA_08	Kelvin Floyd 20/01/2022	Herbfield. −54.281318, −36.510359	1,616,099	0.14	256	192	49.7
*Luzula multiflora*	21F61	16,309	Tissue Bank 16,309	SGSSI: South Georgia	6,385,512	0.17	286	194	50.1
*Montia fontana*	21D68	510729_7	Tissue Bank 16,040	SGSSI: South Georgia	3,883,651	0.21	342	284	75.4
*Nardus stricta*	ERR1744598		Tissue Bank		47,047,436	0.04	316	299	83.5
*Phleum alpinum*	21D59	521532_22	Tissue Bank 16,083	SGSSI: South Georgia	8,994,133	0.13	314	254	68.2
*Poa annua*	21D58	522849_23	Tissue Bank 16,151	SGSSI: South Georgia	9,283,720	0.11	327	275	73.3
*Poa flabellata*	22G53	DNA_50	Kelvin Floyd 12/02/2022	Husdal. −54.183537, −36.712844	4,018,875	0.14	323	283	77.3
*Poa pratensis*	22G34	DNA_13	Kelvin Floyd 24/01/2022	Grytviken. −54.284768, −36.506786	6,586,106	0.19	332	296	78.7
*Poa trivialis*	22G57	DNA_64	Ken Passfield 25/02/2022	Ocean Harbour. −54.340397, −36.275224	3,242,041	0.12	315	284	77.8
*Ranunculus acris*	21B93	49524_3	Tissue Bank 15,834	UK: England: East Sussex	2,024,164	0.06	230	180	53.0
*Ranunculus biternatus*	21D67	510730_1	Tissue Bank 16,042	SGSSI: South Georgia	2,966,785	0.12	263	142	44.9
*Ranunculus repens*	21D35	134794_1	Tissue Bank 15,920	UK: England: West Sussex	2,723,651	0.11	199	111	35.4
*Rostkovia magellanica*	21D87	39,238	Tissue Bank 39,238	SGSSI: South Georgia	2,143,887	0.17	238	106	32.1
*Rumex acetosella*	21D39	65230_1	Tissue Bank 15,889	UK: England: Suffolk	8,926,264	0.14	316	234	62.5
*Rumex crispus*	21D91	36142_7	Tissue Bank 15,778	Colombia: Huila	4,372,137	0.21	330	243	64.5
*Sagina procumbens*	21D64	522919_3	Tissue Bank 16,162	SGSSI: South Georgia	4,527,063	0.18	329	268	69.6
*Scorzoneroides autumnalis*	21D74	647492_6	Tissue Bank 16,250	SGSSI: South Georgia	1,434,133	0.22	286	188	52.3
*Stellaria media*	21D44	350026_9	Tissue Bank 15,978	Jordan: Ajloun	4,454,294	0.13	324	251	66.8
*Taraxacum officinale* agg.	21D63	510567_5	Tissue Bank 16,017	SGSSI: South Georgia	1,614,379	0.18	319	266	67.9
*Trifolium repens*	21D33	29599_13	Tissue Bank 15,762	Greece: Makedhonia	2,624,390	0.22	336	260	70.0
*Tripleurospermum inodorum*	21D43	59237_12	Tissue Bank 15,870	UK: England: West Sussex	2,268,963	0.15	294	177	52.5
*Trisetum spicatum*	21D84	39,347	Tissue Bank 39,347	SGSSI: South Georgia	9,677,611	0.09	297	207	57.8
*Vaccinium vitis‐idaea*	22G29	DNA_19	Kelvin Floyd 27/01/2022	Fellfield. −54.334080, −36.534368	4,484,141	0.39	351	339	89.2
*Veronica serpyllifolia*	21D77	546780_4	Tissue Bank 16,206	SGSSI: South Georgia	2,296,006	0.19	335	289	77.1

### 3.3 Molecular Barcoding of Seedlings

#### 3.3.1 Molecular Barcoding of Seedlings Captured With Wind Traps and Emerging From Soil Samples

Figure S4 represents the phylogenetic tree of all South Georgia species, including samples from seed traps, which confirmed four as *Festuca contracta* , four as *Poa annua* , eight as *Poa flabellata* , and two as *Colobanthus quitensis*.

**FIGURE 3:**
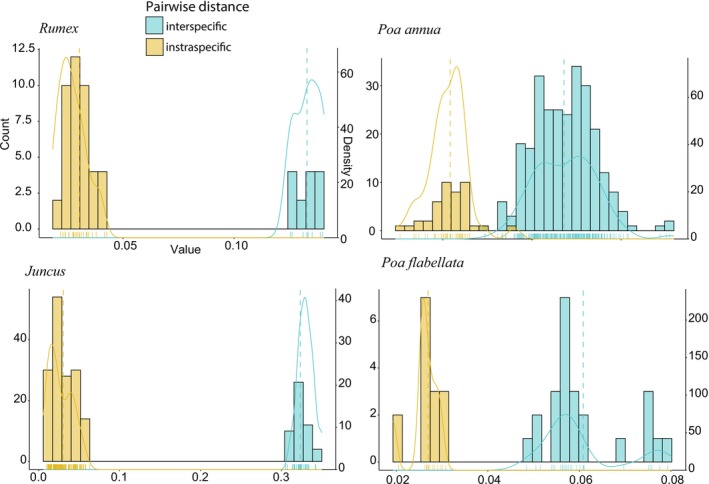
Density plots showing the inter‐ and intraspecific phylogenetic distances for several genera and species calculated from a phylogenetic tree including all seedlings from the soil samples (see Figure S6).

The phylogenetic tree including seedlings from soil samples sequenced with Angiosperms353 and samples from all known species to occur in South Georgia (Figure 3 and Figure S5) allowed the identification of six seedlings as *Ranunculus biternatus*, six in the *Acaena* clade, one as *Scorzoneroides autumnalis*, eight as *Taraxacum officinale* , five as *Rumex acetosella* , one as *Montia fontana* , one in the *Colobanthus subulatus*/ *C. quitensis* clade, three as *Sagina procumbens* , 37 as *Cerastium fontanum* , two as *Rostkovia magellanica*, 11 as *Juncus scheuchzerioides*, two sister to Poaceae or to *Nardus stricta* , two as *Trisetum spicatum* , 13 as *Festuca contracta* , 21 as *Deschampsia antarctica* , 14 as *Phleum alpinum* , eight as *Poa pratensis* , three as *Poa flabellata* , and 44 as *Poa annua* .

Using the identification of seedlings sequenced with Angiosperms353 (Figures S4 and S5), we then confirmed that mixes constructed with the remaining samples belonging to their OTU/site combinations were mixes of samples of the same species (Figure S6 and Table S1). Control mixes containing samples of the same species had a lower SNP density compared to those including DNA of more than one species (Figure S6A), and this gap between intra‐ and interspecific phylogenetic distances was also observed in the mixes of each OTU/site combination (Figure S6B). By plotting the intra‐ and interspecific phylogenetic distances for each mix (Figure S6C), we verified in all cases that interspecific distances to the plastome sequence of any other species occurring in each site were lower than any intraspecific variation against the plastome sequence of the same OTU, thus confirming homogeneity of samples belonging to the same species. Importantly, no new OTUs were detected in replicates 4 or 5 that had not already been identified in replicates 1–3.

Seedlings were assigned to 21 plant species, of which 1256 seedlings were from 10 non‐native species (749 *Cerastium fontanum* , 341 *Poa annua* , 6 *Poa pratensis* , 5 *Deschampsia cespitosa* , 2 *Trisetum spicatum* , 75 *Cardamine glacialis*, 43 *Sagina procumbens* , 1 *Scorzoneroides autumnalis*, 28 *Taraxacum officinale* , 6 *Rumex acetosella* ), 236 seedlings from 11 natives (135 *Juncus scheuchzerioides*, 5 *Rostkovia magellanica*, 23 *Deschampsia antarctica* , 22 *Phleum alpinum* , 5 *Poa flabellata* , 12 *Festuca contracta* , 1 *Galium antarcticum*, 4 *Montia fontana* , 19 *Ranunculus biternatus*, 10 *Acaena magellanica*/ *A. tenera* ), and six non‐identified. Two of the non‐identified samples (21E18 and 21F46) formed a clade sister to all Poaceae. These cases most likely reflect either limited resolution of the markers for closely related Poaceae lineages or gaps in the available genomic reference library.

#### 3.3.2 Incidence of Invasives in the Soil Seed Bank and Caught in Wind Traps

**FIGURE 4:**
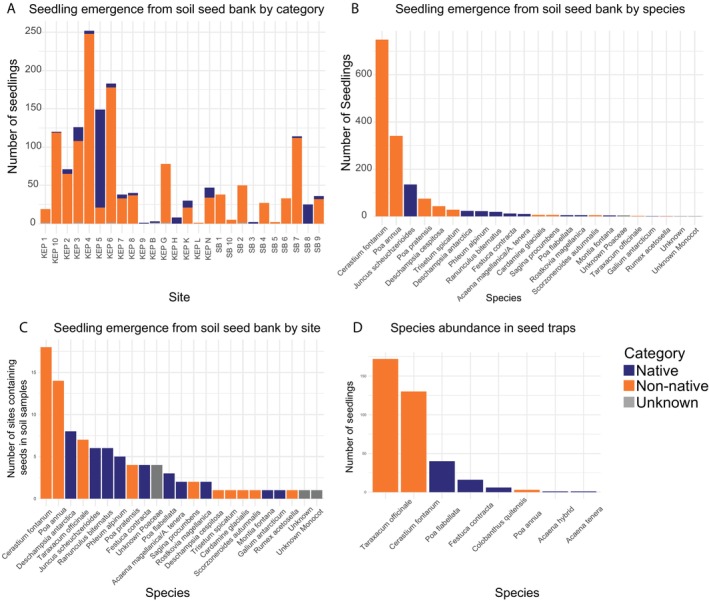
Analysis of invasive species incidence after having identified all seedlings using seedling morphology and molecular barcoding approaches. (A) Bar plots showing the total number of seedlings by category (invasive, native) per collecting site for soil samples. (B) Bar plots showing the total number of seedlings found for each species in soil samples. (C) Incidence of each species across all studied sites for soil samples. (D) Number of seedlings germinated from seed traps per species.

A total of 1498 seedlings emerged from the 130 soil samples, ranging between 1 and 137 seedlings per tray where emergence was observed, with 48 soil trays not producing any seedlings. Most (83.8%) emergent seedlings (1256) were non‐native, with 15.2% comprising native (236 seedlings) species (Figure 4A). Of the non‐native seedlings (1092), 86.9% belonged to the three Class‐III invasive species ( *Cerastium fontanum* , *Poa annua* , *Taraxacum officinale* ), while 4.9% (61 seedlings) belong to Class I and 8.2% (103 seedlings) to Class II (Figure 4B). Class III invasive species were found in soil samples from 18 ( *C. fontanum* ), 14 ( *P. annua* ), and 7 ( *Taraxacum officinale* ) of the 26 sites, respectively, while Class II species were found in 5 sites: *Poa pratensis* in 4 sites and *Trisetum spicatum* in one site. All Class I species (*Cardamine glacialis*, *Deschampsia cespitosa* , *Rumex acetosella* , and *Scozoneroides autumnalis*) were found in only one site except *Sagina procumbens* , which was present in two (Figure 4C).

In total, 737 wind‐dispersed seeds were recovered from 30 seed traps placed in 6 sites across the island. Of these, 243 did not germinate and were found on dissection to be empty—i.e., lacking an embryo and/or endosperm (95 non‐native, 5 native, 143 unidentified). The remaining 494 seeds included 369 that successfully germinated (64 natives and 305 non‐natives), and 42 non‐germinated full seeds that stained red following a Tetrazolium Test (Leist et al. 2003, Miller 2010) were considered viable (Figure S3). Only Class III non‐native species were captured by the wind traps (172 *Taraxacum officinale* , 130 *Cerastium fontanum* seeds); all remaining 67 seeds belonged to six native species (Figure 4D). Non‐native seeds were captured in invaded and recently deglaciated sites and in one of the two native plant species sites.

## 4 Discussion

The findings of this study provide invaluable insights for the long‐term management of invasive plant species on South Georgia, particularly through improved understanding of the soil seed bank as potential reservoirs of non‐native species. By evaluating the resolution of traditional barcoding markers and developing an optimised HTS‐based approach, we were able to detect both native and non‐native species at high taxonomic resolution. These molecular tools not only enhance our ability to monitor and track invasive species but also hold broader potential for biodiversity assessments and conservation management in remote or logistically challenging environments.

### 4.1 Implications for Invasive Species Management

The dominance of seeds of non‐native plant species in the soil seed bank highlights the persistent threat posed by invasive species (Ruwanza 2025). The prevalence of Class III invasive species ( *Cerastium fontanum* , *Poa annua* , and *Taraxacum officinale* ) across multiple sites suggests that these species have not only successfully established but also continue to spread, facilitated by their high seed production, persistent seed banks, and effective dispersal mechanisms (D'Antonio and Meyerson 2002; Pyšek et al. 2012). *Cerastium fontanum* spreads efficiently via stoloniferous growth and extensive seed production, allowing it to persist in nutrient‐rich soils and disturbed areas (Bonis et al. 1997; Tichit et al. 2024). *Poa annua* , with its flexible annual‐to‐perennial life cycle, flowers multiple times per year and thrives in compacted and disturbed soils, an advantage that has contributed to its colonisation of extreme environments, including Antarctica (Chwedorzewska et al. 2014; Molina‐Montenegro et al. 2019; Hughes et al. 2020). *Taraxacum officinale* , a wind‐dispersed perennial, reproduces mainly through apomixis, ensuring rapid colonisation, while its deep taproot enables regrowth following removal (Verduijn et al. 2003; Molina‐Montenegro et al. 2018; Reutova et al. 2023). Their resilience is further reinforced by their ability to rapidly colonise disturbed habitats and exploit the competitive advantage conferred by climate change (Walther et al. 2009; Nosalewicz et al. 2025). Recent genetic investigations of *Poa annua* across sub‐Antarctic islands have demonstrated that human‐mediated multiple introductions strongly shape the genetic diversity, structure, and reproductive strategies of these populations. Mairal et al. (2022) reported that *P. annua* populations at human‐impacted sites, such as station areas, exhibit high genetic diversity and admixture, unlike those in more isolated areas. A broader survey across Southern Ocean archipelagos revealed low differentiation among archipelagos but high within‐archipelago structure, indicating repeated introductions followed by convergent reproductive traits such as outcrossing and polyploidy (Mairal et al. 2023). These traits enhance adaptability and persistence in extreme sub‐Antarctic environments and complicate management, as apomixis enables clonal seed production while polyploidy provides genetic buffering against eradication pressures. These findings suggest that similar processes may underlie the invasion dynamics of other non‐native species on South Georgia, and that such reproductive mechanisms may hinder eradication by facilitating rapid colonisation and long‐term persistence in the soil seed bank. Future population genomic work could test whether the genetic divergence observed among invasive seedlings reflects multiple introduction events, increased propagule pressure via human activity, or both.

Due to the spread and dominance of these Class III invasive species, the South Georgia Non‐Native Plant Management Strategy did not target them directly for eradication, with only targeted removal around some buildings. Therefore, their high occurrence in the soil seed bank and in the bucket traps to collect wind‐dispersed seeds was no surprise. The much higher abundance of non‐native seedlings compared with native ones (1256 vs. 236 in soil and 302 vs. 67 in traps) likely reflects true ecological abundances rather than germination bias. This is consistent with the dominance of *Poa annua* and *Cerastium fontanum* , both Class III invasive species that form dense stands where present and are not targeted for eradication due to their widespread distribution. Nevertheless, we cannot assume that all South Georgian species germinate optimally under the experimental conditions used, and some seeds may have remained dormant, introducing potential germination bias. Soil seed bank persistence also varies among taxa: species with transient seed banks are less likely to be detected if sampling occurs after seed reserves have been depleted (Gioria et al. 2021). The relatively low detection of native seedlings therefore probably reflects both the ecology of many native species and the management focus of our sampling strategy, which was intentionally designed to maximize the likelihood of detecting non‐natives in areas of greatest conservation concern (Figure S1) rather than to comprehensively capture native diversity.

Conversely, the restricted current distribution of Class I and II species in the soil seed bank suggests that management efforts have been largely effective in eradicating (Class I) and limiting non‐native plant species expansion (Class II). While we do not have pre‐eradication soil seed bank data to quantify changes in abundance or range, our findings provide a snapshot of residual invasive seed presence following sustained control interventions. The low frequency of detection for many targeted species likely reflects the impact of management following chemical control. However, the continued presence of viable seeds for several Class I and II species highlights the potential for re‐establishment, underscoring the importance of long‐term monitoring. Dormancy mechanisms in invasive species often enable seeds to persist for years, posing a significant challenge to complete eradication (Galera et al. 2019; Gioria et al. 2021).

Notably, despite the intensive management interventions, several Class I and II species remain detectable in the soil seed bank, including *Poa pratensis* , *Deschampsia cespitosa* , *Trisetum spicatum* , *Cardamine glacialis*, *Sagina procumbens* , *Scorzoneroides autumnalis*, and *Rumex acetosella* . These species exhibit a range of adaptive traits that may contribute to their continued presence. *Poa pratensis* and *Deschampsia cespitosa* , for example, are highly competitive grasses with extensive rhizome networks and prolific seed production, which facilitate rapid regrowth following disturbance (Guglielmin et al. 2008; Pertierra et al. 2013; Xue et al. 2023). *Trisetum spicatum* and *Cardamine glacialis* are known for their ability to colonise harsh environments (Birks 2021; Talebi et al. 2022; Stolsmo et al. 2024), and *Sagina procumbens* and *Rumex acetosella* exhibit considerable tolerance to different habitats, traits that may enhance their ability to survive after eradication activities (Visser et al. 2010; Cooper et al. 2011; Franzese and Ghermandi 2014; Visscher et al. 2023). *Scorzoneroides autumnalis*, with its wind‐dispersed seeds and ability to form persistent seed banks, represents an ongoing risk for reinvasion, particularly in areas where disturbances create open patches of soil, as observed in many other Asteraceae species (Baskin and Baskin, 2023).

No Class I or II species were detected in the seed traps. In the localities surveyed, non‐native plant control measures were scheduled early in the field season to ensure, where possible, that targeted species were removed before flowering and seed set. The only exception was *Cardamine glacialis* in 2017, where some individuals were observed to seed earlier than expected (Floyd K, pers. obs.). The persistence of these Class I and II species in the soil seed bank in these localities likely reflects past seed rain events and the influence of dormancy mechanisms that extend their viability.

Because most soil samples were taken from previously invaded and disturbed sites, we did not assess geographic or altitudinal patterns of seedling emergence. The current distribution of non‐native plants on South Georgia largely reflects their historical introduction around coastal whaling stations, followed by subsequent spread into adjacent native vegetation via reindeer and wind dispersal. Our study also included *Juncus effusus* and *Gaultheria pumila*, sequenced from Falkland Islands material, because plants suspected to belong to these taxa were observed on South Georgia during the study. Both species have since been confirmed on the island and are considered likely non‐native introductions. Their presence highlights the ongoing challenges of distinguishing between native and non‐native taxa in the sub‐Antarctic, where incomplete floristic knowledge and recent arrivals can complicate management decisions.

The low representation of certain Class I and II species in the soil seed bank is unlikely to reflect methodological limitations. Our HTS pipeline achieved consistent recovery of hundreds of nuclear loci across all families, including rare taxa, providing sufficient resolution for confident identification. Therefore, the limited detection of these species most likely reflects their genuinely reduced presence following eradication and control interventions. Nonetheless, we cannot entirely rule out the possibility of very low‐density populations falling below our detection threshold, and continued long‐term monitoring remains warranted. While the current low detection rates of Class I and II species suggest that eradication measures may have had a suppressive effect, we cannot conclusively attribute reductions to management actions in the absence of pre‐eradication data. Nevertheless, the identification of viable seeds from these classes highlights the need to integrate soil seed bank management strategies with continued localized control efforts to eliminate remaining populations (see Results). Incorporating molecular tools to monitor the genetic diversity of invasive populations could provide insights into their adaptability and inform refined management interventions (Estoup and Guillemaud 2010; McGaughran et al. 2024).

South Georgia's unique flora and its ecological significance within the sub‐Antarctic region necessitate proactive measures to mitigate the compounded threats of invasive species and climate change. Accelerated glacial retreat and warming temperatures may create new niches for invasive species, which, combined with human activities, will further amplify their competitive advantage over native flora (Frenot et al. 2005; Tichit et al. 2024). Warmer conditions may extend the growing season and enhance germination rates for non‐native species, allowing them to outcompete slower‐growing native plants that are adapted to colder climates (Pauchard et al. 2016; Duell et al. 2021). The ever‐present threat of new introductions, such as the recent report of the first record of a ladybird beetle (Tichit et al. 2023), highlights the necessity for continued surveillance for non‐native species to prevent their establishment and spread. Adaptive management strategies that incorporate climate projections, habitat restoration, and preventative biosecurity measures will be essential for preserving native biodiversity and maintaining ecosystem resilience in the face of environmental change.

### 4.2 From Traditional Molecular Markers Towards a New HTS‐Based Molecular Barcoding Pipeline to Identify Plant Species

Traditional molecular barcoding methods are widely used for identifying invasive species (e.g., Nath et al. 2024). In this study, *mat*K provided higher resolution than *rbc*L for distinguishing both native and non‐native species in South Georgia, although both markers exhibited the lowest resolution within Poaceae. A meta‐analysis of Arctic flora reported similarly low resolution within Poaceae using these two barcoding regions, with only a marginal increase in species resolution when *mat*K and *rbc*L were combined (~20% vs. 25%) (Saarela et al. 2013). In contrast, the same study demonstrated a high resolution within the genus *Carex*, successfully identifying 85% of the 34 species evaluated using *mat*K, whereas our study was unable to distinguish between closely related species. Similarly, Ranunculaceae showed over twice the species‐level resolution with *mat*K compared to *rbc*L, based on 19 and 20 species, respectively (Saarela et al. 2013). One limitation of this study is that all reference sequences were obtained from GenBank, encompassing multiple geographic origins, where sequencing quality and taxonomic assignments may vary significantly. Furthermore, the efficacy of concatenated *rbc*L with *mat*K sequences, which may increase the resolution of the identifications (Braukmann et al. 2017), was not evaluated due to the limited availability of paired sequences from the same individual and/or study. While traditional barcoding methods were effective for many species, HTS approaches provided finer taxonomic resolution, particularly for species with limited genetic reference data (Antil et al. 2023). The ability of HTS to recover complete plastid genomes and nuclear sequences offers a more robust framework for species identification and phylogenetic analyses, overcoming some of the limitations associated with single‐locus barcoding markers. For these reasons, we did not pursue traditional markers further and instead relied on high‐throughput sequencing (HTS).

A key innovation of this study is the development of a novel bioinformatic pipeline tailored for the assembly, analysis, and phylogenetic placement of HTS data from both native and invasive plant species. By integrating tools such as HybPiper (Johnson et al. 2016) and GetOrganelle (Jin et al. 2020), alongside custom scripts for SNP density estimation and phylogenetic validation, the pipeline ensures robust and reproducible results even with complex datasets. Unlike conventional barcoding approaches that rely on single‐locus plastid markers, this HTS‐based method enables the recovery of hundreds of nuclear loci, greatly improving taxonomic resolution and phylogenetic accuracy (Weitemier et al. 2014; Dodsworth et al. 2019). The use of the universal Angiosperms353 target capture kit makes it particularly valuable for regions with incomplete genetic reference libraries, enabling researchers to generate high‐quality data for conservation planning and ecological monitoring. This approach enables a significant increase in the resolution of phylogenetic trees and the accuracy of molecular barcoding approaches using traditional markers and/or plastome sequence data (Letsiou et al. 2024). Target capture sequencing methods have proven highly effective in accurately identifying samples using molecular barcoding (e.g., in *Anacyclus*, Manzanilla et al. 2022; in *Aloe*, Woudstra et al. 2021), offering a powerful tool for phylogenetic and biodiversity studies across diverse plant lineages.

As sequencing costs continue to decline and computation pipelines become more accessible (Pezzini et al. 2023), Hyb‐Seq approaches are poised to become a cornerstone of high‐throughput molecular barcoding, facilitating the generation of robust, large‐scale reference libraries that will support future ecological and evolutionary studies. Our approach represents a significant methodological advance by integrating genome skimming (Dodsworth 2015) for pooled DNAs with individual sequencing using Hyb‐Seq (Weitemier et al. 2014), greatly enhancing efficiency while maintaining taxonomic resolution. To provide a direct comparison, traditional plastid barcoding with *rbcL* and *mat*K is inexpensive (approximately £5–10 per sample) and fast (about a week from DNA to identification). By contrast, the Angiosperms353 target capture approach required a moderate investment of time (2–3 weeks for library preparation and 3 weeks for sequencing) and cost (£30–40 per individual sample; including DNA extraction, library preparation, enrichment, and sequencing). Genome skimming of pooled samples was slightly cheaper, at ~£20–25 per pool, and faster to process than Angiosperms353, with the added advantage that pooling multiple individuals further reduces effective per‐sample costs. By sequencing only one representative per OTU/site with Angiosperms353 and assigning the remaining samples through pooled genome skimming, we reduced overall costs substantially compared with sequencing all seedlings individually. Additionally, further optimization of laboratory workflows for Angiosperms353 sequencing could be incorporated by refining DNA extraction and library preparation protocols, as outlined by Hale et al. (2020), or by integrating emerging molecular barcoding approaches that enhance efficiency and accuracy (e.g., Gostel et al. 2020). We successfully processed over 1000 samples while sequencing only one representative per site and operational taxonomic unit (OTU). This cost‐effective strategy facilitates rapid and reliable species identification, making it an attractive approach for large‐scale biodiversity monitoring and conservation programmes, particularly in remote or understudied ecosystems where resources are limited.

It is important to acknowledge, however, that our pipeline still depends on germinating seedlings and the expertise required to visually group them into OTUs prior to sequencing. These steps remain relatively hands‐on and may represent a bottleneck for some applications, even if the downstream molecular workflow is streamlined and scalable. A potential limitation of plastome‐based SNP analyses is the presence of nuclear plastid DNA sequences (NUPTs; Richly and Leister 2004), which may map to the plastome and generate spurious variants. In our study, we consider the effect of NUPTs to be minimal. Control mixes with samples of the same species showed no unexpected increases in SNP density, and in morphologically clear cases such as *Cerastium fontanum* , mixed samples were unequivocally assigned to a single species. Moreover, the plastome assemblies generated by GetOrganelle were long and contiguous, and reconstruction of references with HybPiper yielded consistent results (results not shown). These observations support the conclusion that NUPTs did not significantly influence our SNP analyses.

At present, there are no known formal plans to establish long‐term, in situ monitoring of soil seed banks on South Georgia. Nevertheless, all Class I invasive species sites will continue to be revisited as part of management, with systematic checks for seedling emergence. These repeated observations will provide valuable insights into the persistence of soil seed banks of invasive taxa. The molecular barcoding approach developed here further enables rapid identification of seedlings that are otherwise difficult to assign, enhancing the feasibility of such monitoring, and will be universally accessible for future applications across the South Atlantic islands and Antarctic region, particularly following the generation of a comprehensive reference library encompassing all angiosperms established on South Georgia. This genomic resource enhances the utility of our methodology by facilitating the accurate identification of both native and non‐native species across ecologically similar territories. For instance, many of the invasive species sequenced in this study are also of conservation concern in the Falkland Islands and Antarctica, where non‐native flora has been identified as a major threat to fragile ecosystems (Broughton and McAdam 2009; Hughes et al. 2020). By leveraging this growing dataset, conservation practitioners and researchers will be able to implement molecular barcoding for rapid biodiversity assessments and long‐term monitoring efforts in these remote landscapes.

A key stage in any molecular barcoding approach is the availability of reliable reference sequences, which is crucial for ensuring accurate taxonomic identifications. Many publicly available genomic resources, such as those hosted on GenBank or the European Nucleotide Archive (ENA), will facilitate data sharing and reanalysis, ensuring that the molecular resources generated in this study contribute to broader conservation and ecological research initiatives (e.g., Leebens‐Mack et al. 2019; Hinchliff and Smith 2014); however, these repositories vary significantly in quality due to differences in sequencing methodologies, taxonomic validation, and specimen provenance. Misidentified or low‐quality reference sequences can introduce errors in species identification, potentially leading to misinterpretations in biodiversity assessments and potential misallocation of limited resources. The Angiosperms353 bait capture kit has emerged as a standard approach in molecular ecology studies of plants, offering a robust and widely applicable framework for phylogenetic and ecological research (Johnson et al. 2019). As the volume of publicly available genomic data continues to expand, the applicability of this method will further increase, enabling cross‐regional comparisons and more refined assessments of plant biodiversity (Zuntini et al. 2024). When conducting new sequencing efforts, it is essential to use well‐identified plant material to improve the reliability of barcoding datasets (Hollingsworth et al. 2011; Cowell et al. 2022). Ideally, reference samples should be collected from the same geographical region where the study is taking place, as intraspecific genetic variation can differ significantly across biogeographic regions (Lima‐Cordón et al. 2025). In cases where regionally collected samples are unavailable, researchers should prioritise sequences from herbarium voucher specimens with well‐documented taxonomic verification (Dormontt et al. 2018). For this study, we ensured the reliability of our reference sequences by using plant material exclusively collected from South Georgia, taxonomically verified using morphological assessments by experts familiar with the region's flora. Sequencing yield was consistently high across all reference samples, indicating that the DNA extraction and sequencing protocols were effective in capturing the target loci. The high sequencing success of these well‐identified reference samples strengthens the robustness of our molecular barcoding approach and reinforces the value of region‐specific reference libraries in ecological and conservation studies.

The molecular barcoding approach presented in this study will be broadly applicable for future applications across the South Atlantic islands and the wider Antarctic region, particularly following the generation of a comprehensive reference library encompassing all angiosperms established on South Georgia. This genomic resource enhances the utility of our methodology by facilitating the accurate identification of both native and non‐native species across ecologically similar territories. Many of the invasive species sequenced here are also of conservation concern in the Falkland Islands and Antarctica, where non‐native flora has been identified as a major threat to fragile ecosystems (Broughton and McAdam 2009; Hughes et al. 2020). By leveraging this growing dataset, conservation practitioners and researchers will be able to implement molecular barcoding for rapid biodiversity assessments and long‐term monitoring efforts in these remote landscapes. Given the accelerating impacts of climate change and anthropogenic disturbances in sub‐Antarctic ecosystems, molecular barcoding will be an invaluable tool for tracking species distributions, detecting new invasions, and informing adaptive management strategies for threatened plant communities.

### 4.3 Future Directions

Future research should prioritize investigating the genetic diversity and population structure of invasive populations on South Georgia to assess their adaptability, potential for further spread, and genetic responses to management interventions. Understanding any evolutionary trajectories of these species will help refine control strategies and predict future invasion risks under changing environmental conditions. From a management perspective, a crucial recommendation is to avoid soil disturbance and the loss of vegetation cover in areas previously occupied by non‐native populations. Where disturbance is unavoidable, close monitoring and removal of non‐native seedlings, combined with efforts to encourage native vegetation closure, would be essential to limit reinvasion.

Beyond South Georgia, the bioinformatic pipeline developed in this study should be expanded and applied to other regions to evaluate its efficacy in diverse ecological contexts and to refine its analytical capabilities. Its integration with global genetic reference databases and DNA approaches (e.g., environmental DNA, eDNA) could significantly enhance its value for large‐scale biodiversity monitoring and the early detection of invasive species. By combining HTS with ecological modeling, molecular tools can be used not only for detection but also for identifying invasion pathways and priority areas for intervention. Sequencing approaches such as Angiosperms353 and pooled genome skimming offer cost‐effective routes for scaling up such analyses. In our study, Angiosperms353 sequencing provided robust resolution at approximately £30–40 per sample, while pooled genome skimming reduced costs to ~£20–25 with shorter processing times, making both methods well‐suited for conservation applications in resource‐limited or logistically challenging settings.

In addition to these conceptual advances, future work should emphasize increasing computational efficiency, automation, and accessibility of analytical pipelines, enabling conservation practitioners and policymakers to deploy molecular barcoding as a standard tool in long‐term monitoring programmes. As sequencing technologies continue to improve and costs decline, the integration of high‐throughput molecular data into conservation decision‐making will become increasingly feasible, ensuring that management of vulnerable ecosystems, particularly in remote regions such as the sub‐Antarctic, is informed by robust and timely genetic evidence.

## Author Contributions

R.J.N., W.D., C.C., and B.M. secured funding from the UK Government's Biodiversity Challenge Fund, Darwin Plus scheme. K.F. and S.P. conducted fieldwork and collected soil and seed samples in South Georgia. R.J.N., K.W., M.C., and C.K. germinated soil samples and classified seedlings in operational taxonomic units. J.V., F.F. designed the molecular laboratory and bioinformatic experiments. C.J.S., J.V. assessed the use of traditional molecular markers. R.D., J.V. conducted molecular laboratory work and sequencing. J.V. ran the bioinformatic analysis. R.J.N., C.C. analyzed invasive incidence in South Georgia from seedling results; J.V., C.J.S. drafted the manuscript. All co‐authors revised the text.

## Conflicts of Interest

The authors declare no conflicts of interest.

## Supporting information


**Data S1:** Code used to calculate SNP density and produce boxplots and density plots.

**Figure S1:** Map with the collecting sites in South Georgia. Open squares, soil samples; solid circles, wind traps.

**Figure S2:** (A) Phylogenetic tree reconstructed using *mat*K and *rbc*L, and maximum likelihood as implemented in RAxML, and heatmap showing associated pairwise phylogenetic distances between each taxon pair. The width of the circles in nodes represent bootstrap support. (B) pairwise genetic distances calculated between intra‐ and interspecific samples in *Rumex crispus* and *R. acetosella* . (C) pairwise phylogenetic distances calculated between intra‐ and interspecific samples for *Poa pratensis* and *P. trivialis* .

**Figure S3:** Extended phylogenetic tree with all angiosperms known to occur in South Georgia (in orange, non‐natives, in dark blue, natives) and relevant species occurring in neighbouring islands reconstructed using Angiosperms353 sequence data.

**Figure S4:** Phylogenetic tree including all reference species and seedlings from the wind traps (in orange, non‐natives; in dark blue, natives; in light blue, unidentified samples).

**Figure S5:** Phylogenetic tree including all reference species and seedlings from the soil samples (in orange, non‐natives; in dark blue, natives; in light blue, unidentified samples).

**Figure S6:** Analysis of DNA mixes (see Materials and Methods). (A) SNP density values obtained for control samples done with interspecific and intraspecific mixes of seedlings. (B) boxplots with the overall SNP density values obtained for interspecific and intraspecific pairwise comparisons. (C) boxplots with SNP density values for interspecific and intraspecific pairwise comparisons for each mixed DNA sample.

**Table S1:** Results of comparing the SNP density between reference libraries and DNA mixes. Control 1 [RD217 = RD21E07‐5uL = 100 ng] + [RD233 = 21E23‐2.5uL = 102.5]. Control 2 [RD217 = RD21E07‐5uL = 100 ng] + [RD251 = 21E41‐5uL = 85 ng]. Control 3 [RD251 = 21e41‐3uL = 51] + [RD217 = 21E07‐3uL = 60 ng]. Control 4 [RD217 = RD21E07‐1uL = 20 ng] + [RD233 = 21E33‐4.5uL = 184.5 ng].

## Data Availability

Genetic data: Raw sequence reads have been deposited in the European Nucleotide Archive under BioProject PRJEB88856. Benefit‐Sharing Statement: South Georgia is a UK Overseas Territory with no permanent residents or indigenous communities. It is administered by the Government of South Georgia and the South Sandwich Islands (GSGSSI), based in the Falkland Islands. This project was conducted with the full support of GSGSSI and under a Memorandum of Collaboration (MoC) between all partners. The Royal Botanic Gardens, Kew, holds a separate MoC with GSGSSI covering the collection and transfer of materials such as herbarium specimens, seeds, and soil samples. These materials were collected under an annual Regulated Activity Permit issued by GSGSSI in line with Nagoya Protocol compliance, and all specimens have been accessioned and tracked through Kew's collections database. Benefits Generated: (i) Research findings have been shared with GSGSSI and the broader scientific community via formal reports, presentations at SG Terrestrial Protected Area Advisory Group meetings, and through open access publication of all data generated by the project (see Data Accessibility section above); (ii) Public engagement was achieved through blogs, press releases, and newspaper articles aimed at general audiences, including South Georgia visitors; (iii) The project contributes directly to the GSGSSI Terrestrial Protected Area Research and Monitoring Plan; (iv) All partners directly involved with the project are included as co‐authors; (v) The project provided training opportunities for students, who gained hands‐on experience in genomic laboratory techniques and data analysis; these students are also acknowledged as co‐authors.
