# Supplementary material for: Phylogenomic Barcoding of Soil Seed Bank–Persistent and Wind‐Dispersed Non‐Native Plant Species in South Georgia
Source: Mol Ecol Resour. 2025 Nov 8;26(1):e70068. doi: 10.1111/1755-0998.70068 (PMC12627909; doi:10.1111/1755-0998.70068)
Supplement: Supplementary file 1 — Data S1: Code used to calculate SNP density and produce boxplots and density plots. [file MEN-26-e70068-s004.docx]

Data S1. Code used to calculate SNP density and produce boxplots and density plots.

#Get organelle commands#

get_organelle_from_reads.py -1 /beegfs/scratch/scratchFS/users_area/jv12kg/HN00154785/paired/21D51_PAIREDF.fastq -2 /beegfs/scratch/scratchFS/users_area/jv12kg/HN00154785/paired/21D51_PAIREDR.fastq -o plastome_output -R 20 -k 21,45,65,85,105 -F embplant_pt -t 5 -o 21D51

g

## Calculate number of SNPs using nQuire, where List01 contains a list of sample names

bwa mem -t 30 /data/users_area/jv12kg/SG/ref_hybpiper/21D45_Poa_annua.fasta /data/users_area/jv12kg/trimmomatic/21D45_PAIREDF.fastq /data/users_area/jv12kg/trimmomatic/21D45_PAIREDF.fastq > 21D45_21D45.sam

while read i

do samtools sort $i.sam -@ 30 -o ${i%.*}_sorted.bam

done < list.txt

module load nquire

while read i

do

nquire create -b /beegfs/scratch/scratchFS/users_area/jv12kg/SG/maptoref_hybpiper/$i*.bam -o ${i%.*}_nQuire -x

nquire denoise -o ${i%.*}_denoised ${i%.*}_nQuire.bin

nquire histo ${i%_.*}_denoised.bin > histodenoised${i%_.*}.txt

nquire histo ${i%_.*}_nQuire.bin > histonoised${i%_.*}.txt

nquire lrdmodel ${i%_.*}_denoised.bin >> histodenoised${i%_.*}.txt

nquire lrdmodel ${i%_.*}_nQuire.bin >> histonoised${i%_.*}.txt

nquire histotest ${i%_.*}_denoised.bin >> histodenoised${i%_.*}.txt

nquire histotest ${i%_.*}_nQuire.bin >> histonoised${i%_.*}.txt

nquire view ${i%_.*}_denoised.bin > ${i%_.*}_SNPs.txt

nquire view ${i%_.*}_nQuire.bin >> histonoised${i%_.*}.txt

nquire create -b /beegfs/scratch/scratchFS/users_area/jv12kg/SG/maptoref_hybpiper/$i*.bam -o ${i%_.*}_f01_nQuire -x -f 0.1

nquire denoise -o ${i%_.*}_f01_denoised ${i%_.*}_f01_nQuire.bin

nquire view ${i%_.*}_f01_denoised.bin > ${i%_.*}_f01_SNPs.txt

nquire create -b /beegfs/scratch/scratchFS/users_area/jv12kg/SG/maptoref_hybpiper/$i*.bam -o ${i%_.*}_f005_nQuire -x -f 0.05

nquire denoise -o ${i%_.*}_f005_denoised ${i%_.*}_f005_nQuire.bin

nquire view ${i%_.*}_f005_denoised.bin > ${i%_.*}_f005_SNPs.txt

wc -l ${i%_.*}_SNPs.txt >> SNPs_f20.txt

wc -l ${i%_.*}_f01_SNPs.txt >> SNPs_f01.txt

wc -l ${i%_.*}_f005_SNPs.txt >> SNPs_f005.txt

done < ../List01.txt

### Commands to get sequence length from plastome references

for i in *.fasta; do grep -v ">" $i | tr -d '\n' | wc -c > ${i%.*}.txt; done

grep "" *.txt

##Commands to make boxplot graphs using SNP density

library(usedist)

library(reshape2)

library(adephylo)

library(sidier)

library(ggpubr)

library(cowplot)

library(ape)

library(factoextra)

read.csv(file = "mixes_results_nopools.csv")->mixes_results

phist <- gghistogram(mixes_results, x = "SNPdens", add = "mean", rug = TRUE, fill = "dist", palette = c("#00AFBB", "#E7B800"))

pdensity <- ggdensity(mixes_results, x = "SNPdens", color= "dist", palette = c("#00AFBB", "#E7B800"), alpha = 0) + scale_y_continuous(expand = expansion(mult = c(0, 0.05)), position = "right") + theme_half_open(11, rel_small = 1) + rremove("x.axis")+ rremove("xlab") + rremove("x.text") + rremove("x.ticks") + rremove("legend")

aligned_plots <- align_plots(phist, pdensity, align="hv", axis="tblr")

ggdraw(aligned_plots[[1]]) + draw_plot(aligned_plots[[2]])

#Control plots

read.csv(file = "Control1.csv")->Control1

ggplot(Control1, aes(x=as.factor(SP_Ref), y=SNPdens)) +

geom_boxplot(fill="slateblue", alpha=0.2) +

xlab("Mapped against species")

library(ggplot2)

library(dplyr)

library(gridExtra)

library(ggridges)

data <- read.csv("Results_Mixes.csv") # Update with the actual file path

data <- data %>%

mutate(

SNP20_density = SNP20 / Ref.Length,

SNP10_density = SNP10 / Ref.Length,

SNP05_density = SNP05 / Ref.Length

)

data <- data %>%

mutate(

Mix_Type = ifelse(SP.Mixed == SP.Ref, "Intraspecific", "Interspecific"),

Control_Type = case_when(

MIX %in% c("21M94", "21M95", "21M96") ~ "Single-Species Control",

MIX %in% c("21M88", "21M89", "21M90", "21M91") ~ "Mixed-Species Control",

TRUE ~ "Non-Control"

)

)

single_species_controls <- data %>% filter(Control_Type == "Single-Species Control")

control_data <- data %>% filter(Control_Type != "Non-Control")

non_control_data <- data %>% filter(Control_Type == "Non-Control")

control_plots <- list(

SNP20_density = ggplot(control_data, aes(x = Control_Type, y = SNP20_density, fill = Mix_Type)) +

geom_boxplot(aes(color = Mix_Type), outlier.shape = NA) +

labs(title = "Control Data - SNP20 Density", y = "SNP20 Density") +

theme_minimal() +

scale_fill_manual(values = c("Intraspecific" = "lightblue", "Interspecific" = "lightcoral")) +

scale_color_manual(values = c("Intraspecific" = "blue", "Interspecific" = "red")),

SNP10_density = ggplot(control_data, aes(x = Control_Type, y = SNP10_density, fill = Mix_Type)) +

geom_boxplot(aes(color = Mix_Type), outlier.shape = NA) +

labs(title = "Control Data - SNP10 Density", y = "SNP10 Density") +

theme_minimal() +

scale_fill_manual(values = c("Intraspecific" = "lightblue", "Interspecific" = "lightcoral")) +

scale_color_manual(values = c("Intraspecific" = "blue", "Interspecific" = "red")),

SNP05_density = ggplot(control_data, aes(x = Control_Type, y = SNP05_density, fill = Mix_Type)) +

geom_boxplot(aes(color = Mix_Type), outlier.shape = NA) +

labs(title = "Control Data - SNP05 Density", y = "SNP05 Density") +

theme_minimal() +

scale_fill_manual(values = c("Intraspecific" = "lightblue", "Interspecific" = "lightcoral")) +

scale_color_manual(values = c("Intraspecific" = "blue", "Interspecific" = "red"))

)

for (name in names(control_plots)) {

ggsave(paste0(name, "_control_boxplot.pdf"), plot = control_plots[[name]])

ggsave(paste0(name, "_control_boxplot.emf"), plot = control_plots[[name]], device = "emf")

ggsave(paste0(name, "_control_boxplot.jpg"), plot = control_plots[[name]], dpi = 300)

}

##### Script for density plots

read.csv(file = "Poadf.csv")->Poadf

phist <- gghistogram(Poadf, x = "value", add = "mean", rug = TRUE, fill = "dist", palette = c("#00AFBB", "#E7B800","olivedrab4"))

pdensity <- ggdensity(Poadf, x = "value", color= "dist", palette = c("#00AFBB", "#E7B800","olivedrab4"), alpha = 0) + scale_y_continuous(expand = expansion(mult = c(0, 0.05)), position = "right") + theme_half_open(11, rel_small = 1) + rremove("x.axis")+ rremove("xlab") + rremove("x.text") + rremove("x.ticks") + rremove("legend")

aligned_plots <- align_plots(phist, pdensity, align="hv", axis="tblr")

ggdraw(aligned_plots[[1]]) + draw_plot(aligned_plots[[2]])
