# Supplementary figures and images for: Phylogenomic Barcoding of Soil Seed Bank–Persistent and Wind‐Dispersed Non‐Native Plant Species in South Georgia
Source: Mol Ecol Resour. 2025 Nov 8;26(1):e70068. doi: 10.1111/1755-0998.70068 (PMC12627909; doi:10.1111/1755-0998.70068)

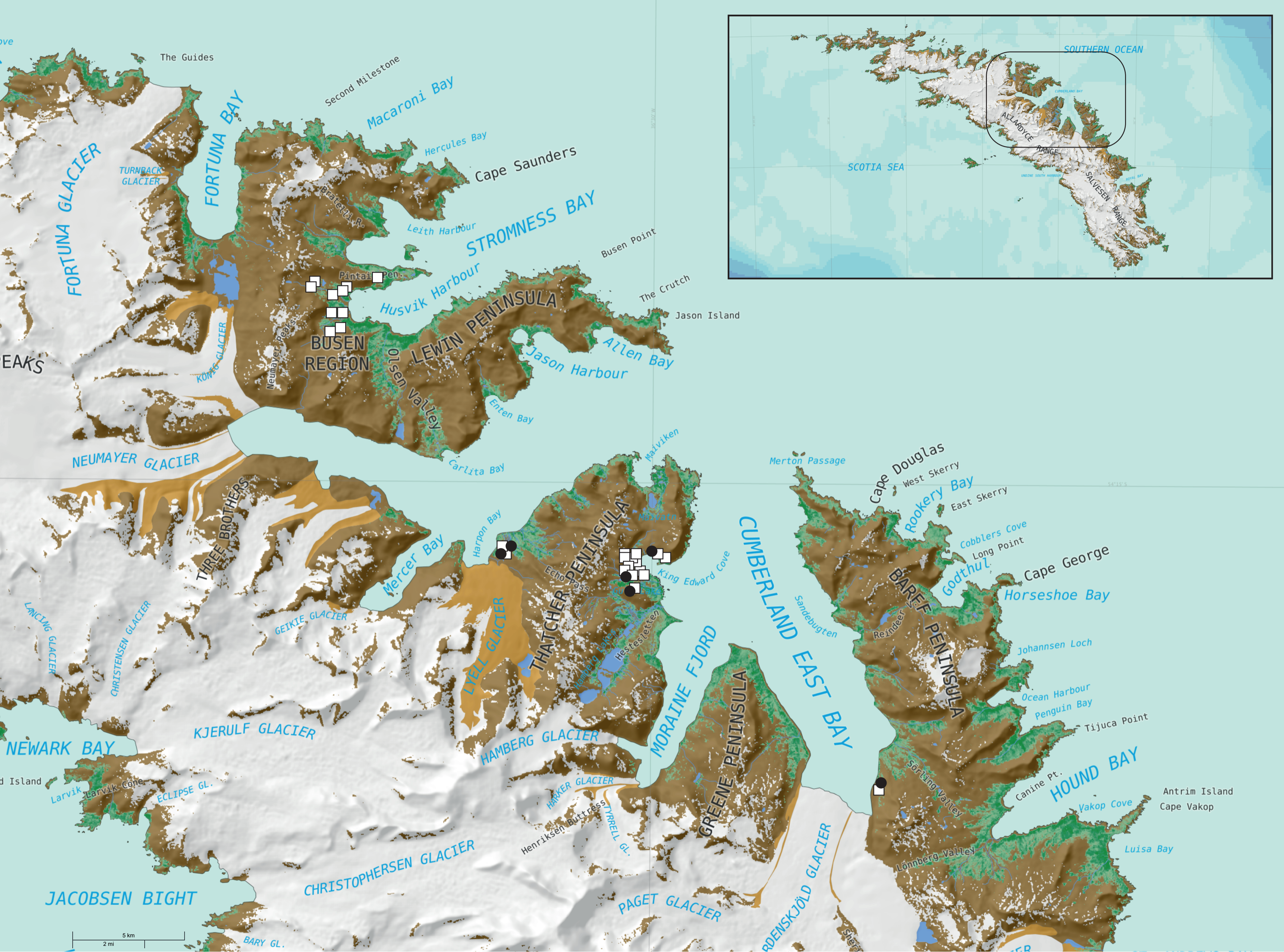

Supplement: Supplementary file 2 — Figure S1: Map with the collecting sites in South Georgia. Open squares, soil samples; solid circles, wind traps. [file MEN-26-e70068-s008.pdf]

matK  
A

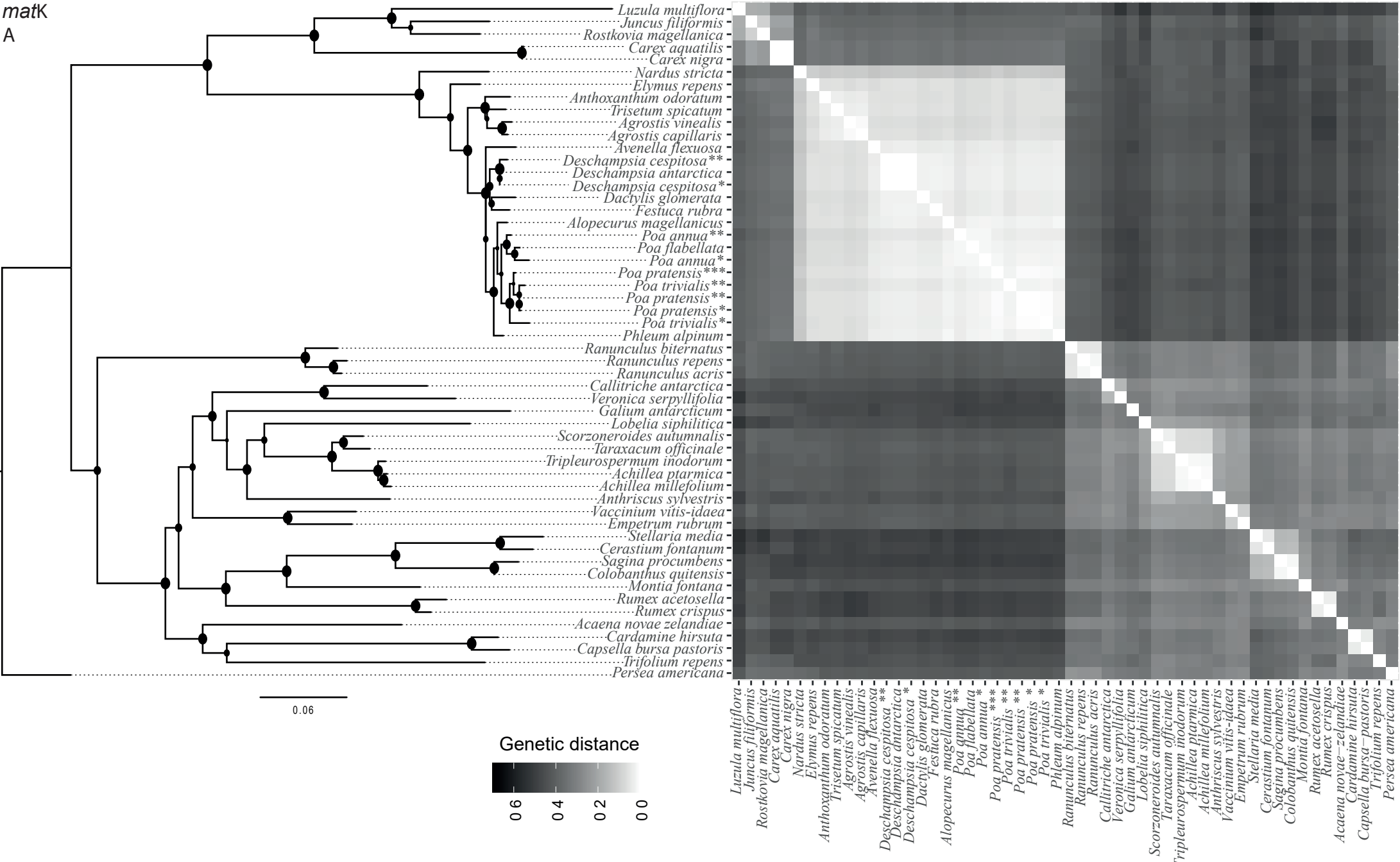

B

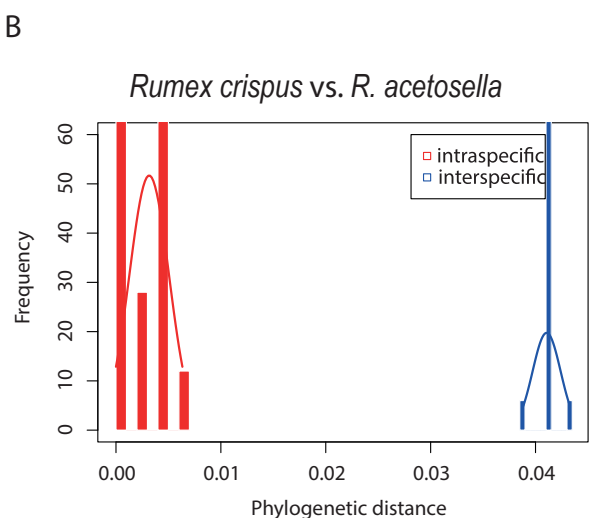

C

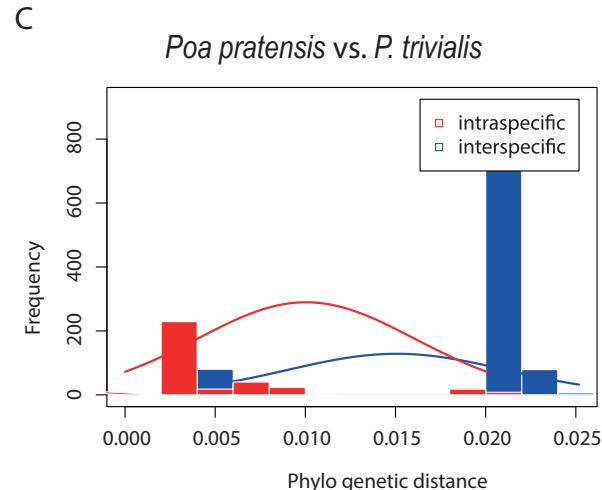

rbcl

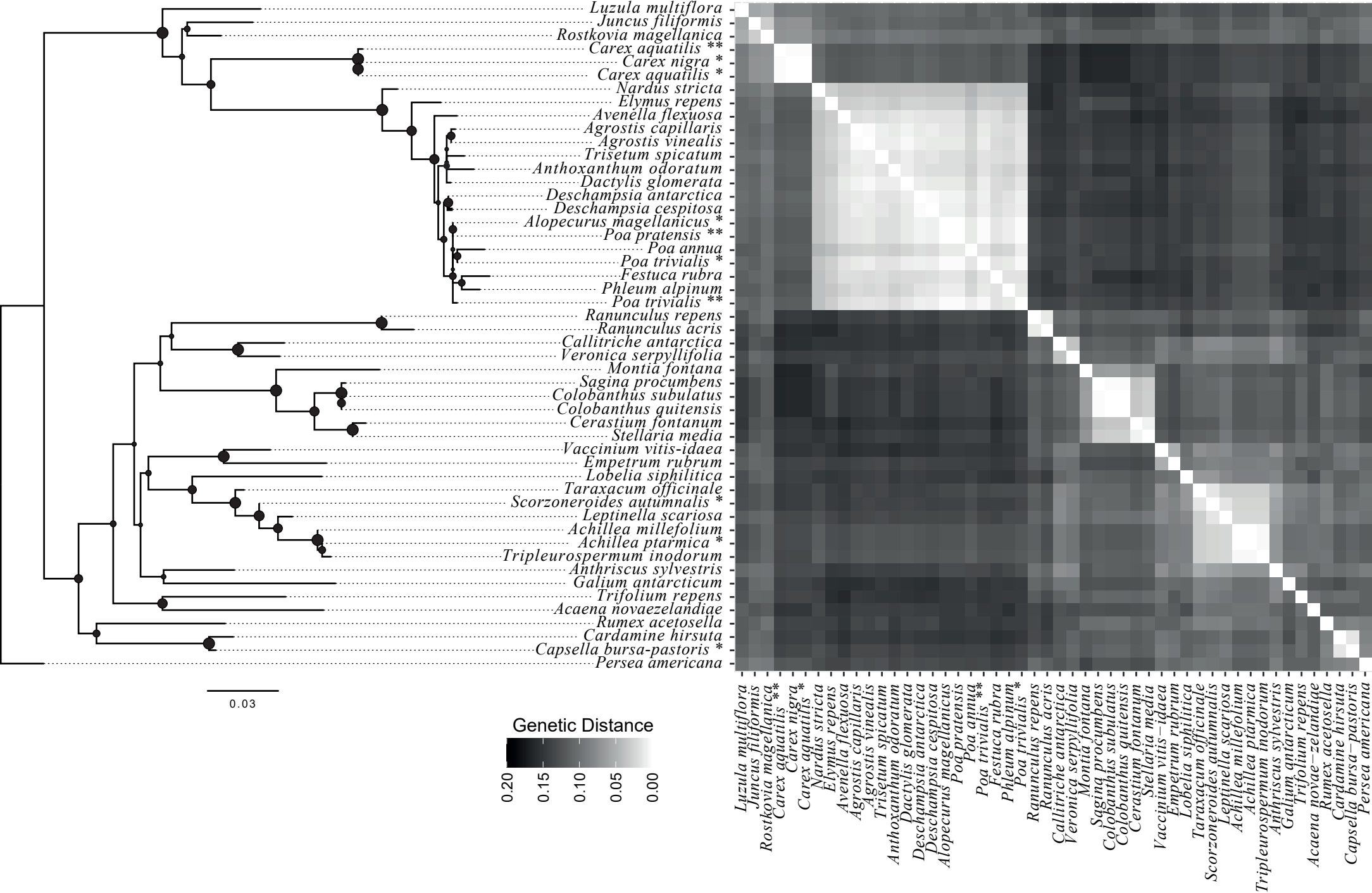

Supplement: Supplementary file 3 — Figure S2: (A) Phylogenetic tree reconstructed using matK and rbcL, and maximum likelihood as implemented in RAxML, and heatmap showing associated pairwise phylogenetic distances between each taxon pair. The width of the circles in nodes represent bootstrap support. (B) pairwise genetic distances calculated between intra‐ and interspecific samples in Rumex crispus and R. acetosella . (C) pairwise phylogenetic distances calculated between intra‐ and interspecific samples for Poa pratensis and P. trivialis . [file MEN-26-e70068-s006.pdf]

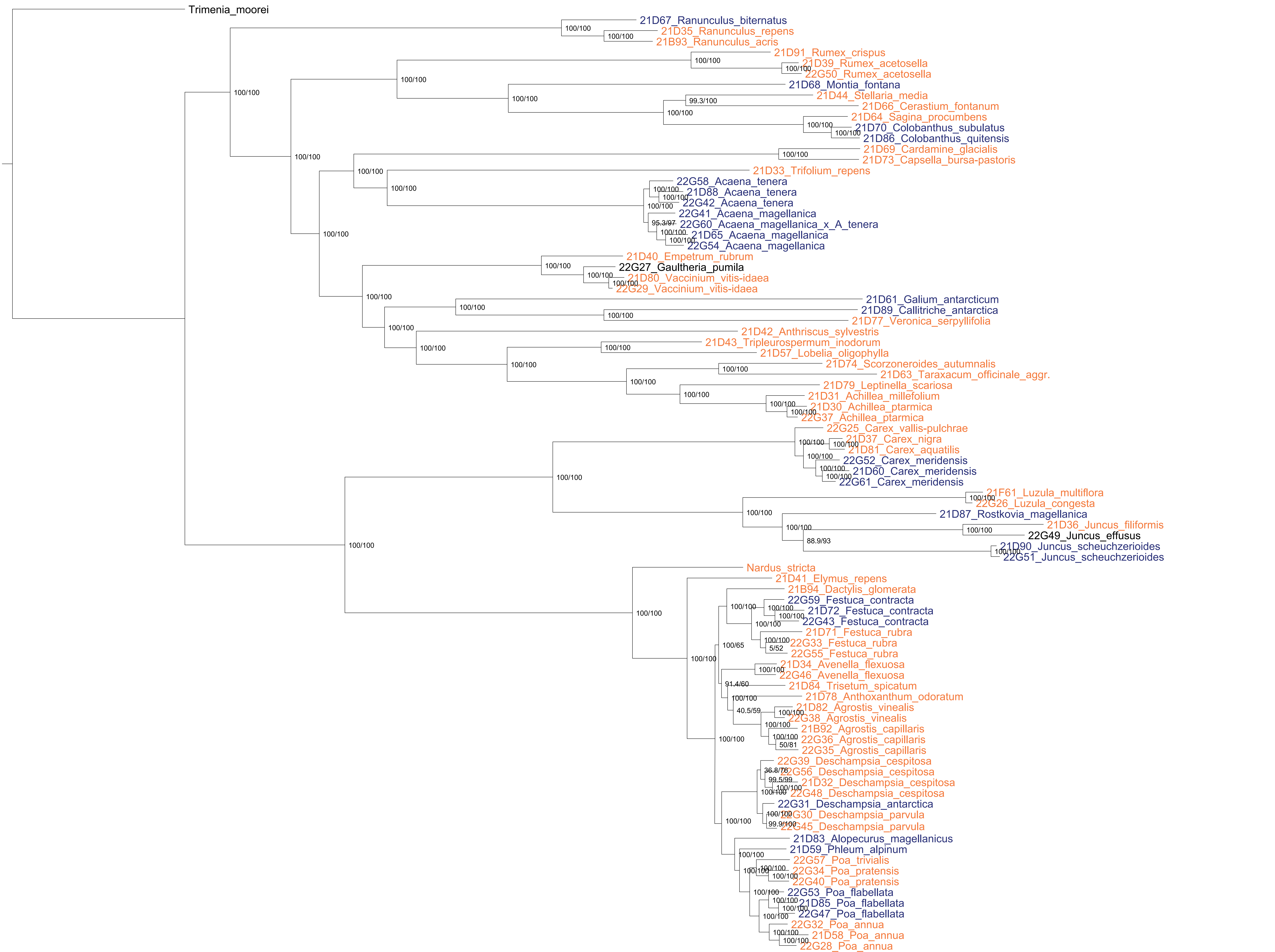

Supplement: Supplementary file 4 — Figure S3: Extended phylogenetic tree with all angiosperms known to occur in South Georgia (in orange, non‐natives, in dark blue, natives) and relevant species occurring in neighbouring islands reconstructed using Angiosperms353 sequence data. [file MEN-26-e70068-s001.pdf]

- *Trimeria moore*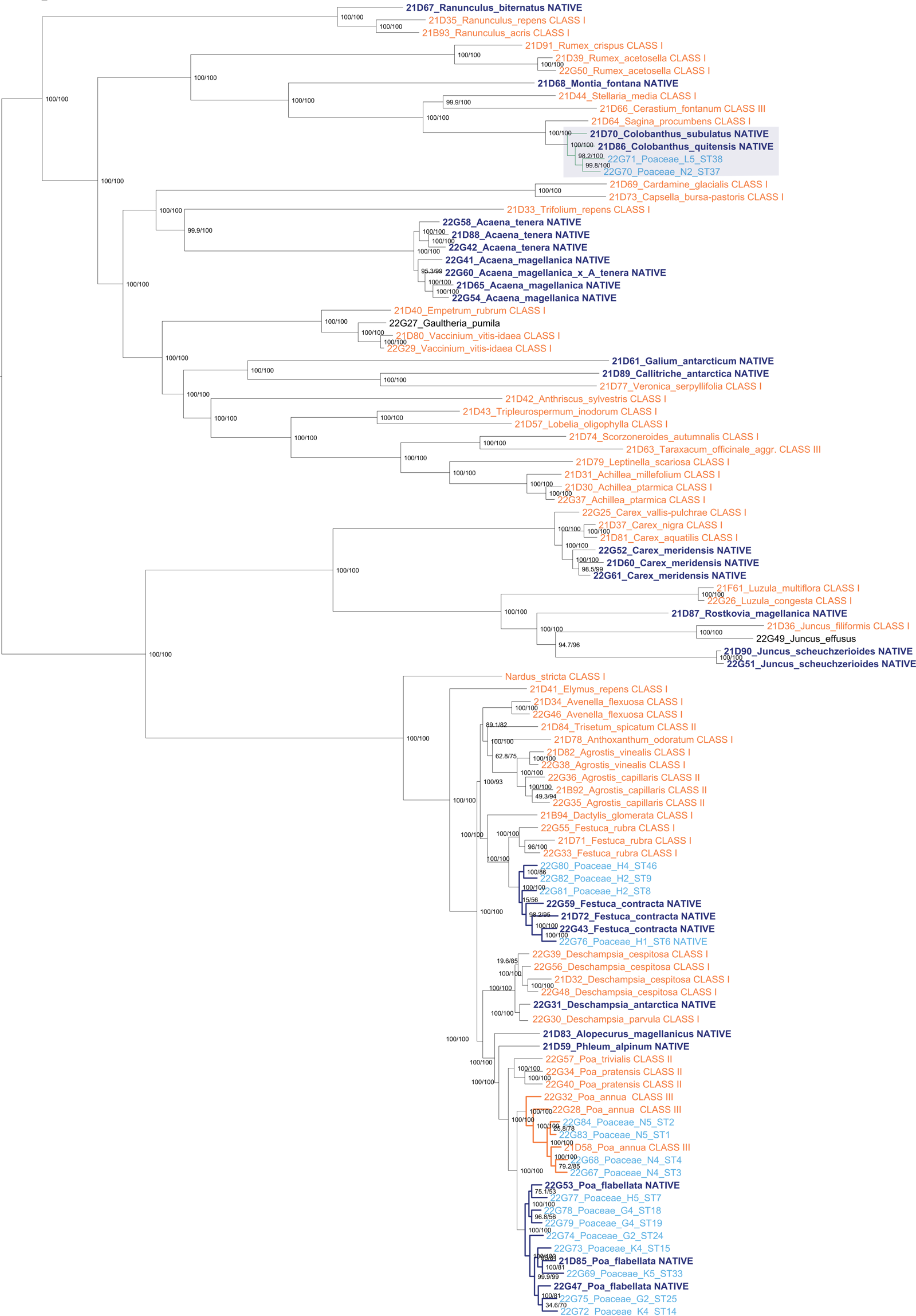

Supplement: Supplementary file 5 — Figure S4: Phylogenetic tree including all reference species and seedlings from the wind traps (in orange, non‐natives; in dark blue, natives; in light blue, unidentified samples). [file MEN-26-e70068-s002.pdf]

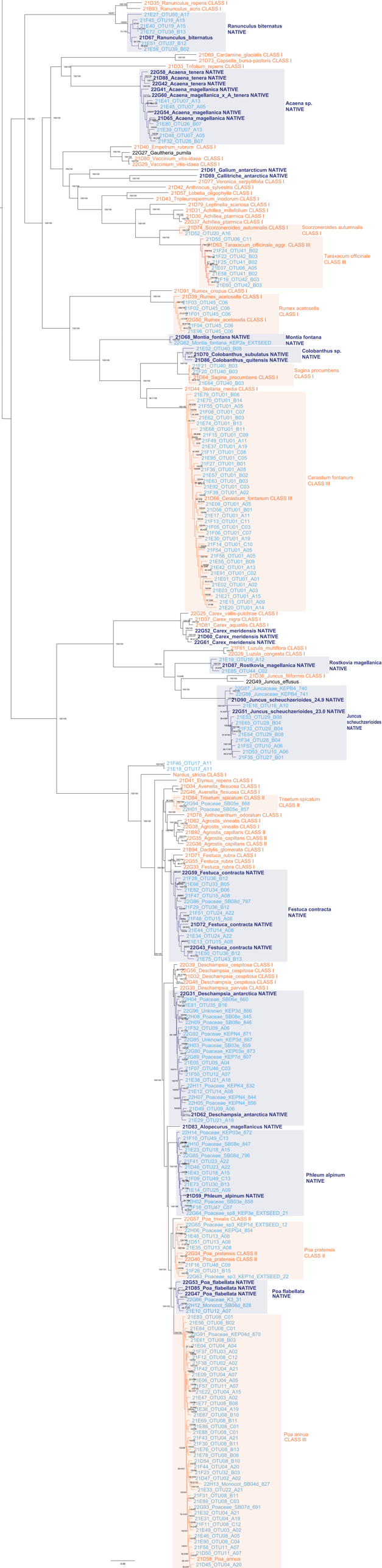

Supplement: Supplementary file 6 — Figure S5: Phylogenetic tree including all reference species and seedlings from the soil samples (in orange, non‐natives; in dark blue, natives; in light blue, unidentified samples). [file MEN-26-e70068-s003.pdf]

A

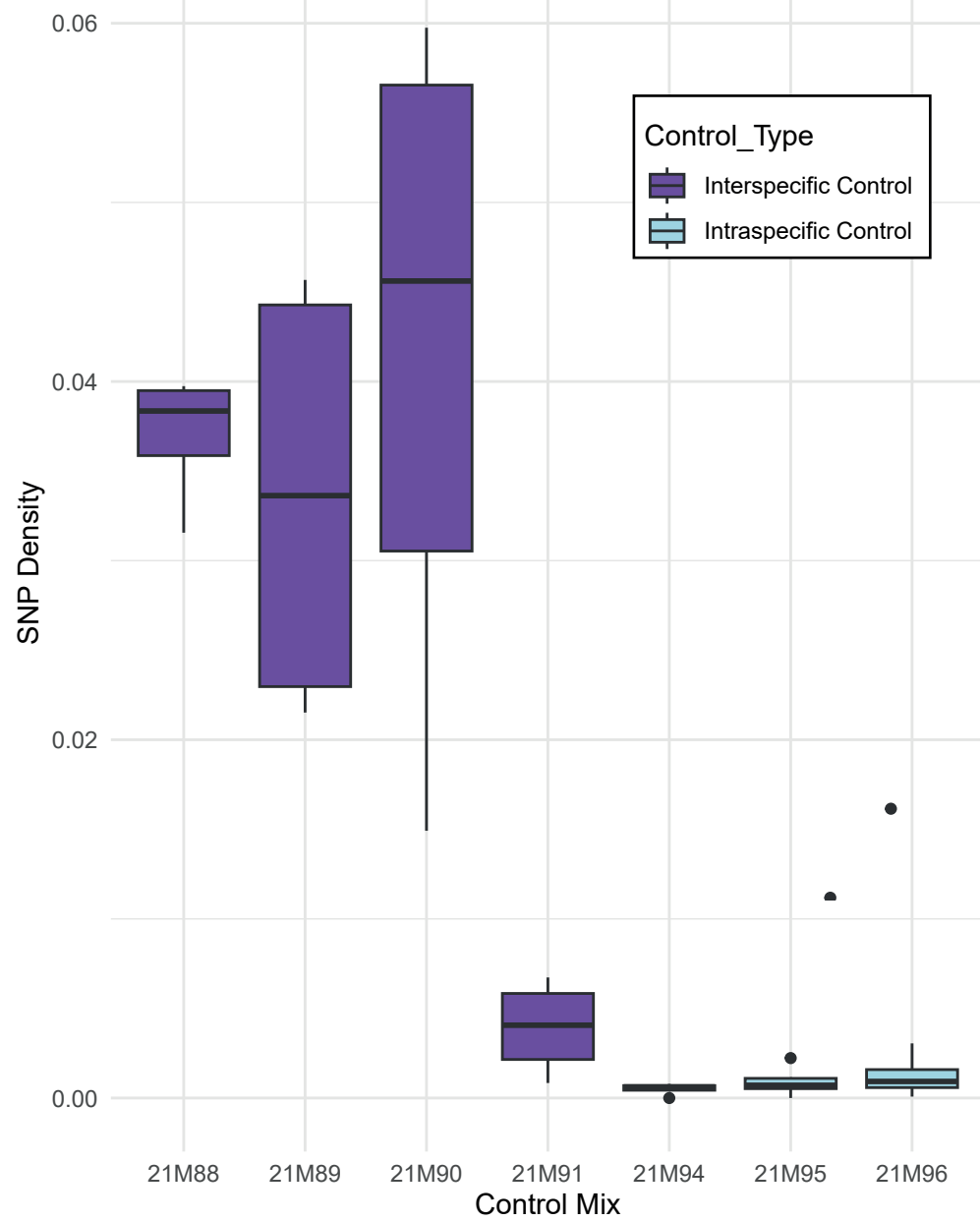

B

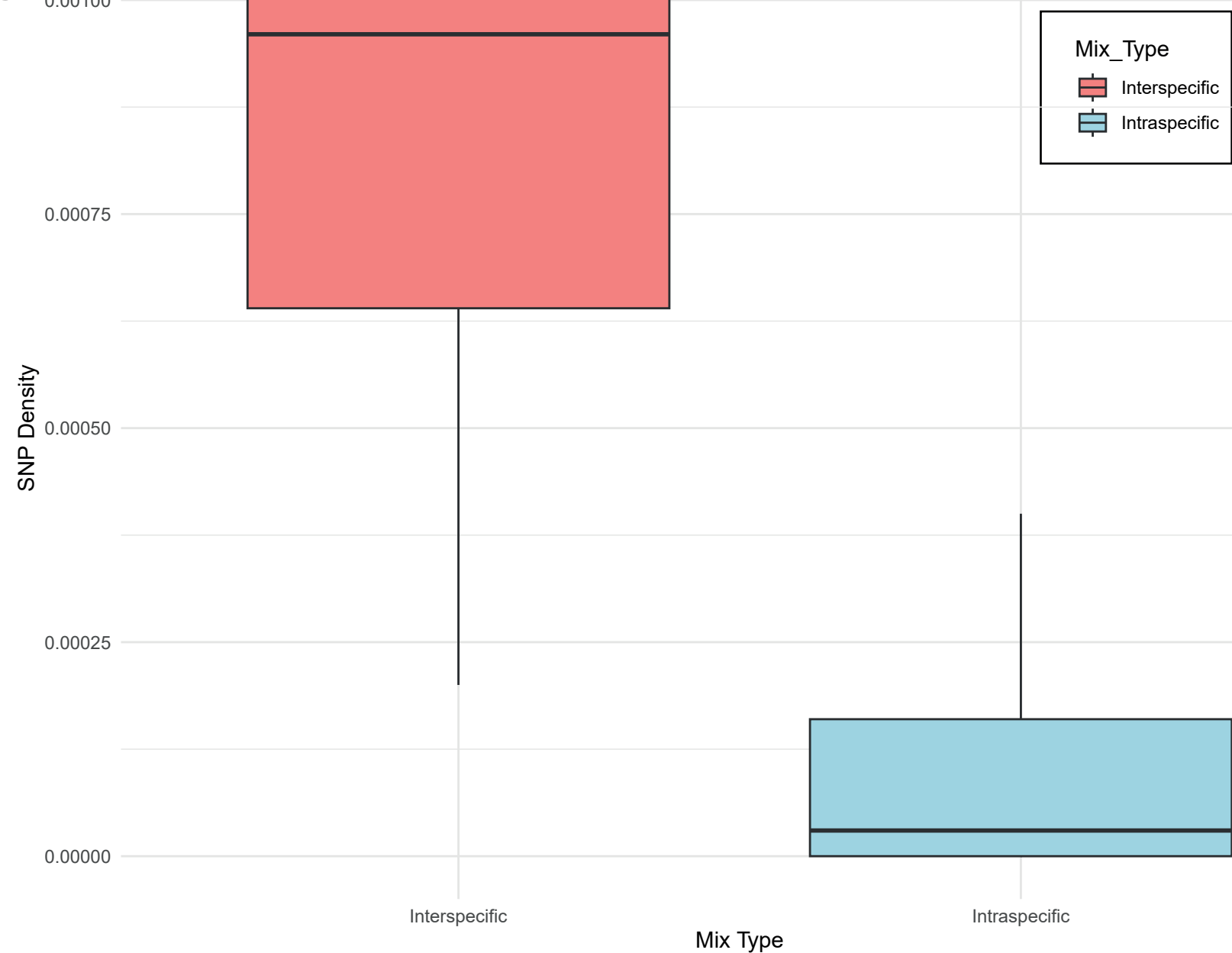

C

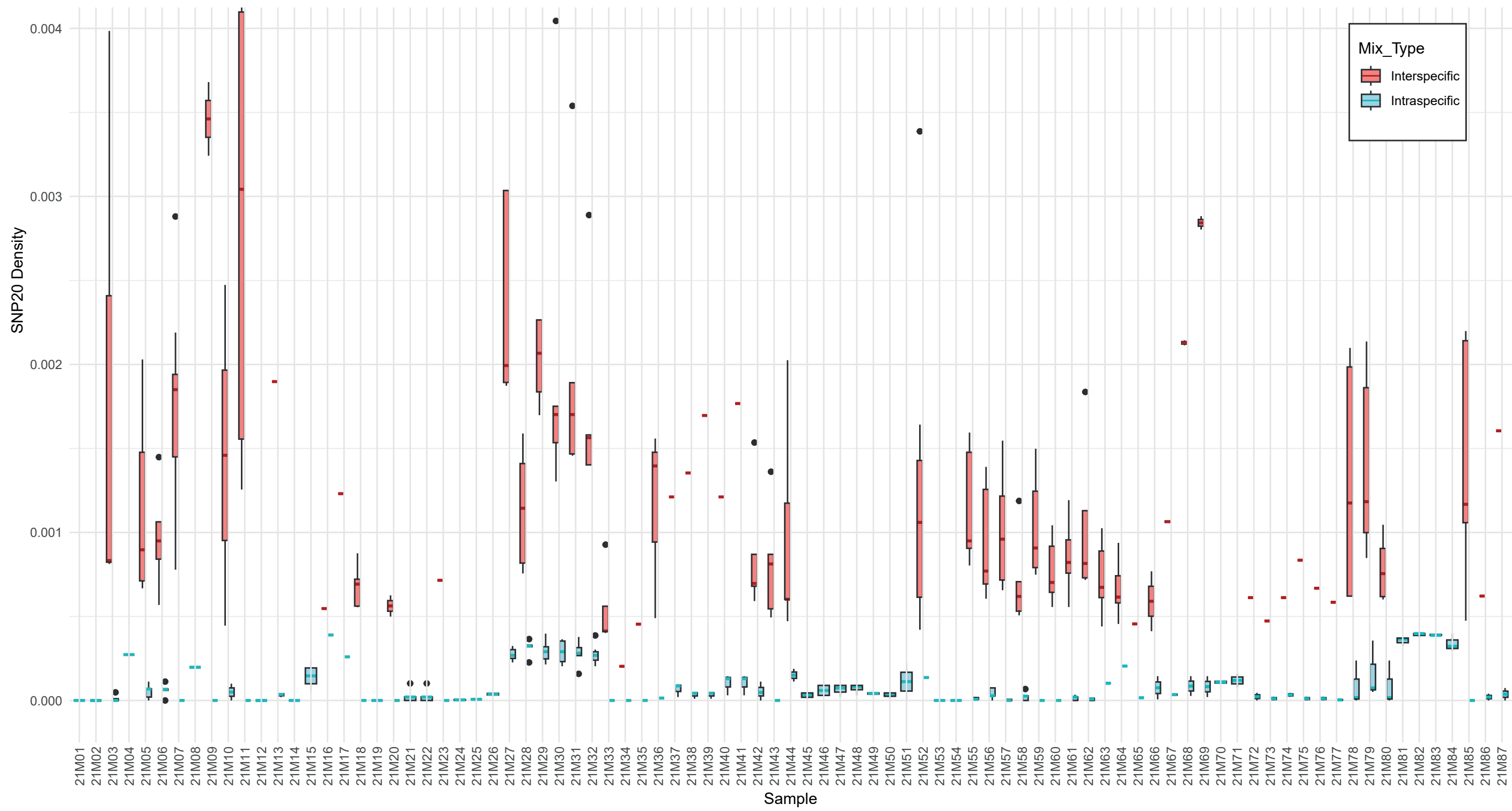

Supplement: Supplementary file 7 — Figure S6: Analysis of DNA mixes (see Materials and Methods). (A) SNP density values obtained for control samples done with interspecific and intraspecific mixes of seedlings. (B) boxplots with the overall SNP density values obtained for interspecific and intraspecific pairwise comparisons. (C) boxplots with SNP density values for interspecific and intraspecific pairwise comparisons for each mixed DNA sample. [file MEN-26-e70068-s005.pdf]
