# Supplementary material for: Phylogenomic Barcoding of Soil Seed Bank–Persistent and Wind‐Dispersed Non‐Native Plant Species in South Georgia
Source: Mol Ecol Resour. 2025 Nov 8;26(1):e70068. doi: 10.1111/1755-0998.70068 (PMC12627909; doi:10.1111/1755-0998.70068)
Supplement: Supplementary file 8 — Table S1: Results of comparing the SNP density between reference libraries and DNA mixes. Control 1 [RD217 = RD21E07‐5uL = 100 ng] + [RD233 = 21E23‐2.5uL = 102.5]. Control 2 [RD217 = RD21E07‐5uL = 100 ng] + [RD251 = 21E41‐5uL = 85 ng]. Control 3 [RD251 = 21e41‐3uL = 51] + [RD217 = 21E07‐3uL = 60 ng]. Control 4 [RD217 = RD21E07‐1uL = 20 ng] + [RD233 = 21E33‐4.5uL = 184.5 ng]. [file MEN-26-e70068-s007.docx]

| Table S1. Results of comparing the SNP density between reference libraries and DNA mixes. Control 1 [RD217=RD21E07-5uL=100ng]+[RD233=21E23-2.5uL=102.5]. Control 2 [RD217=RD21E07-5uL=100ng]+[RD251=21E41-5uL=85ng]. Control 3 [RD251=21e41-3uL=51]+[RD217=21E07-3uL=60ng]. Control 4 [RD217=RD21E07-1uL=20ng]+[RD233=21E33-4.5uL=184.5ng] | | | | | | | | | | | | |
| --- | --- | --- | --- | --- | --- | --- | --- | --- | --- | --- | --- | --- |
| MIX | OTU | SITE | SP Mixed | SP Ref | OTU ref | SITE ref | Seq ref | SNP20 | SNP10 | Ref Length | SNPdens20 | SNPdens10 |
| 21M01 | OTU01 | A01 | *Cerastium fontanum* | *Cerastium fontanum* | OTU01 | A01 | 21E01 | 0 | 4 | 9115 | 0.00000 | 0.00044 |
| 21M01 | OTU01 | A01 | *Cerastium fontanum* | *Cerastium fontanum* | OTU01 | A05 | 21E08 | 0 | 16 | 115955 | 0.00000 | 0.00014 |
| 21M02 | OTU01 | A01 | *Cerastium fontanum* | *Cerastium fontanum* | OTU01 | A01 | 21E01 | 0 | 0 | 9115 | 0.00000 | 0.00000 |
| 21M02 | OTU01 | A01 | *Cerastium fontanum* | *Cerastium fontanum* | OTU01 | A05 | 21E08 | 0 | 5 | 115955 | 0.00000 | 0.00004 |
| 21M03 | OTU02 | A02 | *Poa annua* | *Cerastium fontanum* | OTU01 | A02 | 21F39 | 120 | 350 | 147683 | 0.00081 | 0.00237 |
| 21M03 | OTU02 | A02 | *Poa annua* | *Cerastium fontanum* | OTU01 | A02 | 21F40 | 145 | 386 | 36388 | 0.00398 | 0.01061 |
| 21M03 | OTU02 | A02 | *Poa annua* | *Poa annua* | OTU02 | A02 | 21D47 | 1 | 50 | 95628 | 0.00001 | 0.00052 |
| 21M03 | OTU02 | A02 | *Poa annua* | *Poa annua* | OTU03 | A02 | 21E47 | 1 | 2 | 20736 | 0.00005 | 0.00010 |
| 21M03 | OTU02 | A02 | *Poa annua* | *Poa annua* | OTU03 | A02 | 21E49 | 0 | 0 | 11815 | 0.00000 | 0.00000 |
| 21M03 | OTU02 | A02 | *Poa annua* | *Poa annua* | OTU03 | A02 | 21F37 | 0 | 0 | 12290 | 0.00000 | 0.00000 |
| 21M03 | OTU02 | A02 | *Poa annua* | *Poa annua* | OTU02 | A02 | 21F38 | 0 | 0 | 6392 | 0.00000 | 0.00000 |
| 21M03 | OTU02 | A02 | *Poa annua* | *Cerastium fontanum* | OTU01 | A11 | 21F49 | 123 | 290 | 147683 | 0.00083 | 0.00196 |
| 21M04 | OTU01 | A03 | *Cerastium fontanum* | *Cerastium fontanum* | OTU01 | A03 | 21E03 | 4 | 6 | 8692 | 0.00046 | 0.00069 |
| 21M05 | OTU01 | A05 | *Cerastium fontanum* | *Acaena magellanica* | OTU07 | A05 | 21D48 | 85 | 145 | 94784 | 0.00090 | 0.00153 |
| 21M05 | OTU01 | A05 | *Cerastium fontanum* | *Acaena magellanica* | OTU07 | A05 | 21E45 | 51 | 79 | 34525 | 0.00148 | 0.00229 |
| 21M05 | OTU01 | A05 | *Cerastium fontanum* | *Cerastium fontanum* | OTU01 | A05 | 21E08 | 8 | 12 | 115955 | 0.00007 | 0.00010 |
| 21M05 | OTU01 | A05 | *Cerastium fontanum* | *Cerastium fontanum* | OTU01 | A05 | 21F36 | 3 | 9 | 148121 | 0.00002 | 0.00006 |
| 21M05 | OTU01 | A05 | *Cerastium fontanum* | *Cerastium fontanum* | OTU01 | A05 | 21F54 | 4 | 4 | 35623 | 0.00011 | 0.00011 |
| 21M05 | OTU01 | A05 | *Cerastium fontanum* | *Cerastium fontanum* | OTU01 | A05 | 21F55 | 0 | 0 | 45048 | 0.00000 | 0.00000 |
| 21M05 | OTU01 | A05 | *Cerastium fontanum* | *Cerastium fontanum* | OTU01 | A05 | 21F56 | 10 | 16 | 147039 | 0.00007 | 0.00011 |
| 21M05 | OTU01 | A05 | *Cerastium fontanum* | *Poa annua* | OTU04 | A05 | 21E06 | 42 | 71 | 20695 | 0.00203 | 0.00343 |
| 21M05 | OTU01 | A05 | *Cerastium fontanum* | *Poa annua* | OTU08 | A05 | 21E46 | 54 | 119 | 80926 | 0.00067 | 0.00147 |
| 21M05 | OTU01 | A05 | *Cerastium fontanum* | *Taraxacum officinale* | OTU06 | A05 | 21E07 | 77 | 170 | 108195 | 0.00071 | 0.00157 |
| 21M06 | OTU01 | A05 | *Cerastium fontanum* | *Acaena magellanica* | OTU07 | A05 | 21D48 | 90 | 168 | 94784 | 0.00095 | 0.00177 |
| 21M06 | OTU01 | A05 | *Cerastium fontanum* | *Acaena magellanica* | OTU07 | A05 | 21E45 | 50 | 82 | 34525 | 0.00145 | 0.00238 |
| 21M06 | OTU01 | A05 | *Cerastium fontanum* | *Cerastium fontanum* | OTU01 | A05 | 21E08 | 7 | 17 | 115955 | 0.00006 | 0.00015 |
| 21M06 | OTU01 | A05 | *Cerastium fontanum* | *Cerastium fontanum* | OTU01 | A05 | 21F36 | 0 | 0 | 148121 | 0.00000 | 0.00000 |
| 21M06 | OTU01 | A05 | *Cerastium fontanum* | *Cerastium fontanum* | OTU01 | A05 | 21F54 | 4 | 4 | 35623 | 0.00011 | 0.00011 |
| 21M06 | OTU01 | A05 | *Cerastium fontanum* | *Cerastium fontanum* | OTU01 | A05 | 21F55 | 3 | 14 | 45048 | 0.00007 | 0.00031 |
| 21M06 | OTU01 | A05 | *Cerastium fontanum* | *Cerastium fontanum* | OTU01 | A05 | 21F56 | 10 | 21 | 147039 | 0.00007 | 0.00014 |
| 21M06 | OTU01 | A05 | *Cerastium fontanum* | *Poa annua* | OTU04 | A05 | 21E06 | 22 | 61 | 20695 | 0.00106 | 0.00295 |
| 21M06 | OTU01 | A05 | *Cerastium fontanum* | *Poa annua* | OTU08 | A05 | 21E46 | 46 | 134 | 80926 | 0.00057 | 0.00166 |
| 21M06 | OTU01 | A05 | *Cerastium fontanum* | *Taraxacum officinale* | OTU06 | A05 | 21E07 | 91 | 207 | 108195 | 0.00084 | 0.00191 |
| 21M07 | OTU06 | A05 | *Taraxacum officinale* | *Acaena magellanica* | OTU07 | A05 | 21D48 | 105 | 153 | 94784 | 0.00111 | 0.00161 |
| 21M07 | OTU06 | A05 | *Taraxacum officinale* | *Acaena magellanica* | OTU07 | A05 | 21E45 | 67 | 76 | 34525 | 0.00194 | 0.00220 |
| 21M07 | OTU06 | A05 | *Taraxacum officinale* | *Cerastium fontanum* | OTU01 | A05 | 21E08 | 334 | 578 | 115955 | 0.00288 | 0.00498 |
| 21M07 | OTU06 | A05 | *Taraxacum officinale* | *Cerastium fontanum* | OTU01 | A05 | 21F36 | 274 | 494 | 148121 | 0.00185 | 0.00334 |
| 21M07 | OTU06 | A05 | *Taraxacum officinale* | *Cerastium fontanum* | OTU01 | A05 | 21F54 | 78 | 161 | 35623 | 0.00219 | 0.00452 |
| 21M07 | OTU06 | A05 | *Taraxacum officinale* | *Cerastium fontanum* | OTU01 | A05 | 21F55 | 82 | 170 | 45048 | 0.00182 | 0.00377 |
| 21M07 | OTU06 | A05 | *Taraxacum officinale* | *Cerastium fontanum* | OTU01 | A05 | 21F56 | 276 | 495 | 147039 | 0.00188 | 0.00337 |
| 21M07 | OTU06 | A05 | *Taraxacum officinale* | *Poa annua* | OTU04 | A05 | 21E06 | 30 | 64 | 20695 | 0.00145 | 0.00309 |
| 21M07 | OTU06 | A05 | *Taraxacum officinale* | *Poa annua* | OTU08 | A05 | 21E46 | 63 | 140 | 80926 | 0.00078 | 0.00173 |
| 21M07 | OTU06 | A05 | *Taraxacum officinale* | *Taraxacum officinale* | OTU06 | A05 | 21E07 | 0 | 1 | 108195 | 0.00000 | 0.00001 |
| 21M08 | OTU10 | A12 | *Rostkovia magellanica* | *Rostkovia magellanica* | OTU10 | A12 | 21E19 | 20 | 120 | 101325 | 0.00020 | 0.00118 |
| 21M09 | OTU01 | A13 | *Cerastium fontanum* | *Acaena magellanica* | OTU07 | A13 | 21E39 | 94 | 100 | 28994 | 0.00324 | 0.00345 |
| 21M09 | OTU01 | A13 | *Cerastium fontanum* | *Acaena magellanica* | OTU07 | A13 | 21E41 | 94 | 114 | 25540 | 0.00368 | 0.00446 |
| 21M09 | OTU01 | A13 | *Cerastium fontanum* | *Cerastium fontanum* | OTU01 | A13 | 21E42 | 0 | 1 | 10920 | 0.00000 | 0.00009 |
| 21M10 | OTU23 | A22 | *Phleum alpinum* | *Festuca contracta* | OTU24 | A22 | 21E34 | 51 | 90 | 114557 | 0.00045 | 0.00079 |
| 21M10 | OTU23 | A22 | *Phleum alpinum* | *Festuca contracta* | OTU24 | A22 | 21F51 | 40 | 95 | 16174 | 0.00247 | 0.00587 |
| 21M10 | OTU23 | A22 | *Phleum alpinum* | *Phleum alpinum* | OTU23 | A22 | 21D46 | 0 | 68 | 99072 | 0.00000 | 0.00069 |
| 21M10 | OTU23 | A22 | *Phleum alpinum* | *Phleum alpinum* | OTU23 | A22 | 21F41 | 9 | 86 | 90179 | 0.00010 | 0.00095 |
| 21M11 | OTU08 | B03 | *Poa annua* | *Cerastium fontanum* | OTU01 | B03 | 21E62 | 140 | 234 | 46855 | 0.00299 | 0.00499 |
| 21M11 | OTU08 | B03 | *Poa annua* | *Cerastium fontanum* | OTU01 | B03 | 21E63 | 126 | 185 | 30976 | 0.00407 | 0.00597 |
| 21M11 | OTU08 | B03 | *Poa annua* | *Poa annua* | OTU08 | B03 | 21E61 | 0 | 10 | 85721 | 0.00000 | 0.00012 |
| 21M11 | OTU08 | B03 | *Poa annua* | *Poa annua* | OTU32 | B03 | 21F23 | 0 | 4 | 9829 | 0.00000 | 0.00041 |
| 21M11 | OTU08 | B03 | *Poa annua* | *Sagina procumbens* | OTU40 | B03 | 21E64 | 109 | 169 | 8415 | 0.01295 | 0.02008 |
| 21M11 | OTU08 | B03 | *Poa annua* | *Sagina procumbens* | OTU40 | B03 | 21F20 | 112 | 189 | 26746 | 0.00419 | 0.00707 |
| 21M11 | OTU08 | B03 | *Poa annua* | *Sagina procumbens* | OTU40 | B03 | 21F21 | 182 | 341 | 58768 | 0.00310 | 0.00580 |
| 21M11 | OTU08 | B03 | *Poa annua* | *Taraxacum officinale* | OTU42 | B03 | 21E60 | 187 | 416 | 119099 | 0.00157 | 0.00349 |
| 21M11 | OTU08 | B03 | *Poa annua* | *Taraxacum officinale* | OTU42 | B03 | 21F19 | 188 | 411 | 124259 | 0.00151 | 0.00331 |
| 21M11 | OTU08 | B03 | *Poa annua* | *Taraxacum officinale* | OTU42 | B03 | 21F22 | 190 | 420 | 151339 | 0.00126 | 0.00278 |
| 21M12 | OTU26 | B07 | *Acaena magellanica* | *Acaena magellanica* | OTU26 | B07 | 21E80 | 0 | 0 | 28476 | 0.00000 | 0.00000 |
| 21M12 | OTU26 | B07 | *Acaena magellanica* | *Acaena magellanica* | OTU26 | B07 | 21F32 | 0 | 0 | 28236 | 0.00000 | 0.00000 |
| 21M13 | OTU36 | B12 | *Festuca contracta* | *Festuca contracta* | OTU36 | B12 | 21E50 | 2 | 21 | 102272 | 0.00002 | 0.00021 |
| 21M13 | OTU36 | B12 | *Festuca contracta* | *Festuca contracta* | OTU36 | B12 | 21F28 | 1 | 19 | 22686 | 0.00004 | 0.00084 |
| 21M13 | OTU36 | B12 | *Festuca contracta* | *Festuca contracta* | OTU36 | B12 | 21F29 | 1 | 17 | 27752 | 0.00004 | 0.00061 |
| 21M13 | OTU36 | B12 | *Festuca contracta* | *Ranunculus biternatus* | OTU37 | B12 | 21E51 | 185 | 478 | 97480 | 0.00190 | 0.00490 |
| 21M14 | OTU08 | C04 | *Poa annua* | *Poa annua* | OTU08 | C04 | 21E90 | 0 | 0 | 6392 | 0.00000 | 0.00000 |
| 21M14 | OTU08 | C04 | *Poa annua* | *Poa annua* | OTU08 | C04 | 21E93 | 0 | 0 | 3521 | 0.00000 | 0.00000 |
| 21M15 | OTU49 | C13 | *Phleum alpinum* | *Phleum alpinum* | OTU49 | C13 | 21F09 | 1 | 28 | 19232 | 0.00005 | 0.00146 |
| 21M15 | OTU49 | C13 | *Phleum alpinum* | *Phleum alpinum* | OTU49 | C13 | 21F10 | 8 | 70 | 33212 | 0.00024 | 0.00211 |
| 21M16 | OTU01 | A17 | *Vaccinium sp* | *Ranunculus biternatus* | OTU50 | A17 | 21E27 | 8 | 27 | 14626 | 0.00055 | 0.00185 |
| 21M16 | OTU01 | A17 | *Vaccinium sp* | *Vaccinium sp* | OTU01 | A17 | 21E26 | 57 | 117 | 146286 | 0.00039 | 0.00080 |
| 21M17 | OTU01 | A17 | *Vaccinium sp* | *Ranunculus biternatus* | OTU50 | A17 | 21E27 | 18 | 32 | 14626 | 0.00123 | 0.00219 |
| 21M17 | OTU01 | A17 | *Vaccinium sp* | *Vaccinium sp* | OTU01 | A17 | 21E26 | 38 | 78 | 146286 | 0.00026 | 0.00053 |
| 21M18 | OTU01 | B02 | *Cerastium fontanum* | *Cerastium fontanum* | OTU01 | B02 | 21E57 | 0 | 0 | 10012 | 0.00000 | 0.00000 |
| 21M18 | OTU01 | B02 | *Cerastium fontanum* | *Poa annua* | OTU08 | B02 | 21E56 | 17 | 38 | 19407 | 0.00088 | 0.00196 |
| 21M18 | OTU01 | B02 | *Cerastium fontanum* | *Ranunculus biternatus* | OTU39 | B02 | 21E59 | 89 | 169 | 123293 | 0.00072 | 0.00137 |
| 21M18 | OTU01 | B02 | *Cerastium fontanum* | *Taraxacum officinale* | OTU41 | B02 | 21E58 | 83 | 180 | 149501 | 0.00056 | 0.00120 |
| 21M18 | OTU01 | B02 | *Cerastium fontanum* | *Taraxacum officinale* | OTU41 | B02 | 21F24 | 85 | 185 | 151363 | 0.00056 | 0.00122 |
| 21M18 | OTU01 | B02 | *Cerastium fontanum* | *Taraxacum officinale* | OTU41 | B02 | 21F25 | 79 | 177 | 114094 | 0.00069 | 0.00155 |
| 21M19 | OTU01 | C08 | *Cerastium fontanum* | *Cerastium fontanum* | OTU01 | C08 | 21F17 | 0 | 4 | 11811 | 0.00000 | 0.00034 |
| 21M20 | OTU01 | C09 | *Cerastium fontanum* | *Cerastium fontanum* | OTU01 | C09 | 21F15 | 0 | 1 | 43609 | 0.00000 | 0.00002 |
| 21M20 | OTU01 | C09 | *Cerastium fontanum* | *Poa pratensis* | OTU48 | C09 | 21F16 | 5 | 15 | 7989 | 0.00063 | 0.00188 |
| 21M20 | OTU01 | C09 | *Cerastium fontanum* | *Poa pratensis* | OTU13 | A08 | 21D51 | 34 | 85 | 68085 | 0.00050 | 0.00125 |
| 21M21 | OTU08 | C12 | *Poa annua* | *Poa annua* | OTU08 | C12 | 21F11 | 0 | 0 | 6392 | 0.00000 | 0.00000 |
| 21M21 | OTU08 | C12 | *Poa annua* | *Poa annua* | OTU08 | C12 | 21F12 | 0 | 0 | 6617 | 0.00000 | 0.00000 |
| 21M21 | OTU08 | C12 | *Poa annua* | *Poa annua* | OTU08 | C01 | 21E84 | 2 | 7 | 97283 | 0.00002 | 0.00007 |
| 21M21 | OTU08 | C12 | *Poa annua* | *Poa annua* | OTU08 | C01 | 21E86 | 9 | 16 | 88698 | 0.00010 | 0.00018 |
| 21M21 | OTU08 | C12 | *Poa annua* | *Poa annua* | OTU08 | C01 | 21E88 | 2 | 12 | 95698 | 0.00002 | 0.00013 |
| 21M22 | OTU08 | C12 | *Poa annua* | *Poa annua* | OTU08 | C12 | 21F11 | 0 | 0 | 6392 | 0.00000 | 0.00000 |
| 21M22 | OTU08 | C12 | *Poa annua* | *Poa annua* | OTU08 | C12 | 21F12 | 0 | 0 | 6617 | 0.00000 | 0.00000 |
| 21M22 | OTU08 | C12 | *Poa annua* | *Poa annua* | OTU08 | C01 | 21E84 | 2 | 7 | 97283 | 0.00002 | 0.00007 |
| 21M22 | OTU08 | C12 | *Poa annua* | *Poa annua* | OTU08 | C01 | 21E86 | 9 | 16 | 88698 | 0.00010 | 0.00018 |
| 21M22 | OTU08 | C12 | *Poa annua* | *Poa annua* | OTU08 | C01 | 21E88 | 2 | 12 | 95698 | 0.00002 | 0.00013 |
| 21M23 | OTU01 | C02 | *Cerastium fontanum* | *Cerastium fontanum* | OTU01 | C02 | 21E91 | 0 | 24 | 42117 | 0.00000 | 0.00057 |
| 21M23 | OTU01 | C02 | *Cerastium fontanum* | *Rostkovia magellanica* | OTU44 | C02 | 21E85 | 74 | 151 | 103480 | 0.00072 | 0.00146 |
| 21M24 | OTU01 | B09 | *Cerastium fontanum* | *Cerastium fontanum* | OTU01 | B09 | 21E55 | 0 | 10 | 146356 | 0.00000 | 0.00007 |
| 21M24 | OTU01 | B09 | *Cerastium fontanum* | *Cerastium fontanum* | OTU01 | A11 | 21F49 | 1 | 4 | 147683 | 0.00001 | 0.00003 |
| 21M25 | OTU01 | B09 | *Cerastium fontanum* | *Cerastium fontanum* | OTU01 | B09 | 21E55 | 1 | 16 | 146356 | 0.00001 | 0.00011 |
| 21M25 | OTU01 | B09 | *Cerastium fontanum* | *Cerastium fontanum* | OTU01 | A11 | 21F49 | 1 | 4 | 147683 | 0.00001 | 0.00003 |
| 21M26 | OTU10 | B09 | *Cerastium fontanum* | *Cerastium fontanum* | OTU01 | B09 | 21E55 | 7 | 16 | 146356 | 0.00005 | 0.00011 |
| 21M26 | OTU10 | B09 | *Cerastium fontanum* | *Cerastium fontanum* | OTU01 | A11 | 21F49 | 4 | 7 | 147683 | 0.00003 | 0.00005 |
| 21M27 | OTU10 | A06 | *Juncus scheuchzerioides* | *Deschampsia* | OTU09 | A06 | 21D49 | 130 | 261 | 42831 | 0.00304 | 0.00609 |
| 21M27 | OTU10 | A06 | *Juncus scheuchzerioides* | *Deschampsia* | OTU09 | A06 | 21F52 | 75 | 159 | 13846 | 0.00542 | 0.01148 |
| 21M27 | OTU10 | A06 | *Juncus scheuchzerioides* | *Juncus scheuchzerioides* | OTU10 | A06 | 21D53 | 10 | 155 | 44174 | 0.00023 | 0.00351 |
| 21M27 | OTU10 | A06 | *Juncus scheuchzerioides* | *Juncus scheuchzerioides* | OTU10 | A06 | 21F53 | 8 | 88 | 30170 | 0.00027 | 0.00292 |
| 21M27 | OTU10 | A06 | *Juncus scheuchzerioides* | *Juncus scheuchzerioides* | OTU28 | B04 | 21E65 | 23 | 278 | 97980 | 0.00023 | 0.00284 |
| 21M27 | OTU10 | A06 | *Juncus scheuchzerioides* | *Juncus scheuchzerioides* | OTU28 | B04 | 21F33 | 25 | 265 | 93054 | 0.00027 | 0.00285 |
| 21M27 | OTU10 | A06 | *Juncus scheuchzerioides* | *Juncus scheuchzerioides* | OTU28 | B04 | 21F34 | 27 | 268 | 92994 | 0.00029 | 0.00288 |
| 21M27 | OTU10 | A06 | *Juncus scheuchzerioides* | *Juncus scheuchzerioides* | OTU29 | B08 | 21E53 | 30 | 276 | 92502 | 0.00032 | 0.00298 |
| 21M27 | OTU10 | A06 | *Juncus scheuchzerioides* | *Juncus scheuchzerioides* | OTU29 | B08 | 21E54 | 25 | 223 | 79388 | 0.00031 | 0.00281 |
| 21M27 | OTU10 | A06 | *Juncus scheuchzerioides* | *Deschampsia* | OTU05 | A04 | 21E05 | 171 | 349 | 91291 | 0.00187 | 0.00382 |
| 21M27 | OTU10 | A06 | *Juncus scheuchzerioides* | *Deschampsia* | OTU29 | A21 | 21E29 | 169 | 354 | 89286 | 0.00189 | 0.00396 |
| 21M27 | OTU10 | A06 | *Juncus scheuchzerioides* | *Deschampsia* | OTU35 | B16 | 21E81 | 164 | 348 | 82266 | 0.00199 | 0.00423 |
| 21M28 | OTU10 | A06 | *Juncus scheuchzerioides* | *Deschampsia* | OTU09 | A06 | 21D49 | 49 | 297 | 42831 | 0.00114 | 0.00693 |
| 21M28 | OTU10 | A06 | *Juncus scheuchzerioides* | *Deschampsia* | OTU09 | A06 | 21F52 | 22 | 171 | 13846 | 0.00159 | 0.01235 |
| 21M28 | OTU10 | A06 | *Juncus scheuchzerioides* | *Juncus scheuchzerioides* | OTU10 | A06 | 21D53 | 10 | 181 | 44174 | 0.00023 | 0.00410 |
| 21M28 | OTU10 | A06 | *Juncus scheuchzerioides* | *Juncus scheuchzerioides* | OTU10 | A06 | 21F53 | 10 | 214 | 30170 | 0.00033 | 0.00709 |
| 21M28 | OTU10 | A06 | *Juncus scheuchzerioides* | *Juncus scheuchzerioides* | OTU28 | B04 | 21E65 | 31 | 363 | 97980 | 0.00032 | 0.00370 |
| 21M28 | OTU10 | A06 | *Juncus scheuchzerioides* | *Juncus scheuchzerioides* | OTU28 | B04 | 21F33 | 34 | 363 | 93054 | 0.00037 | 0.00390 |
| 21M28 | OTU10 | A06 | *Juncus scheuchzerioides* | *Juncus scheuchzerioides* | OTU28 | B04 | 21F34 | 30 | 356 | 92994 | 0.00032 | 0.00383 |
| 21M28 | OTU10 | A06 | *Juncus scheuchzerioides* | *Juncus scheuchzerioides* | OTU29 | B08 | 21E53 | 30 | 342 | 92502 | 0.00032 | 0.00370 |
| 21M28 | OTU10 | A06 | *Juncus scheuchzerioides* | *Juncus scheuchzerioides* | OTU29 | B08 | 21E54 | 26 | 321 | 79388 | 0.00033 | 0.00404 |
| 21M28 | OTU10 | A06 | *Juncus scheuchzerioides* | *Deschampsia* | OTU05 | A04 | 21E05 | 69 | 572 | 91291 | 0.00076 | 0.00627 |
| 21M28 | OTU10 | A06 | *Juncus scheuchzerioides* | *Deschampsia* | OTU29 | A21 | 21E29 | 73 | 554 | 89286 | 0.00082 | 0.00620 |
| 21M28 | OTU10 | A06 | *Juncus scheuchzerioides* | *Deschampsia* | OTU35 | B16 | 21E81 | 116 | 560 | 82266 | 0.00141 | 0.00681 |
| 21M29 | OTU10 | A06 | *Juncus scheuchzerioides* | *Deschampsia* | OTU09 | A06 | 21D49 | 97 | 202 | 42831 | 0.00226 | 0.00472 |
| 21M29 | OTU10 | A06 | *Juncus scheuchzerioides* | *Deschampsia* | OTU09 | A06 | 21F52 | 79 | 149 | 13846 | 0.00571 | 0.01076 |
| 21M29 | OTU10 | A06 | *Juncus scheuchzerioides* | *Juncus scheuchzerioides* | OTU10 | A06 | 21D53 | 10 | 90 | 44174 | 0.00023 | 0.00204 |
| 21M29 | OTU10 | A06 | *Juncus scheuchzerioides* | *Juncus scheuchzerioides* | OTU10 | A06 | 21F53 | 12 | 116 | 30170 | 0.00040 | 0.00384 |
| 21M29 | OTU10 | A06 | *Juncus scheuchzerioides* | *Juncus scheuchzerioides* | OTU28 | B04 | 21E65 | 21 | 45 | 97980 | 0.00021 | 0.00046 |
| 21M29 | OTU10 | A06 | *Juncus scheuchzerioides* | *Juncus scheuchzerioides* | OTU28 | B04 | 21F33 | 25 | 55 | 93054 | 0.00027 | 0.00059 |
| 21M29 | OTU10 | A06 | *Juncus scheuchzerioides* | *Juncus scheuchzerioides* | OTU28 | B04 | 21F34 | 27 | 65 | 92994 | 0.00029 | 0.00070 |
| 21M29 | OTU10 | A06 | *Juncus scheuchzerioides* | *Juncus scheuchzerioides* | OTU29 | B08 | 21E53 | 30 | 98 | 92502 | 0.00032 | 0.00106 |
| 21M29 | OTU10 | A06 | *Juncus scheuchzerioides* | *Juncus scheuchzerioides* | OTU29 | B08 | 21E54 | 25 | 42 | 79388 | 0.00031 | 0.00053 |
| 21M29 | OTU10 | A06 | *Juncus scheuchzerioides* | *Deschampsia* | OTU05 | A04 | 21E05 | 155 | 281 | 91291 | 0.00170 | 0.00308 |
| 21M29 | OTU10 | A06 | *Juncus scheuchzerioides* | *Deschampsia* | OTU29 | A21 | 21E29 | 164 | 302 | 89286 | 0.00184 | 0.00338 |
| 21M29 | OTU10 | A06 | *Juncus scheuchzerioides* | *Deschampsia* | OTU35 | B16 | 21E81 | 170 | 333 | 82266 | 0.00207 | 0.00405 |
| 21M30 | OTU10 | A06 | *Juncus scheuchzerioides* | *Deschampsia* | OTU09 | A06 | 21D49 | 75 | 162 | 42831 | 0.00175 | 0.00378 |
| 21M30 | OTU10 | A06 | *Juncus scheuchzerioides* | *Deschampsia* | OTU09 | A06 | 21F52 | 56 | 92 | 13846 | 0.00404 | 0.00664 |
| 21M30 | OTU10 | A06 | *Juncus scheuchzerioides* | *Juncus scheuchzerioides* | OTU10 | A06 | 21D53 | 9 | 112 | 44174 | 0.00020 | 0.00254 |
| 21M30 | OTU10 | A06 | *Juncus scheuchzerioides* | *Juncus scheuchzerioides* | OTU10 | A06 | 21F53 | 11 | 103 | 30170 | 0.00036 | 0.00341 |
| 21M30 | OTU10 | A06 | *Juncus scheuchzerioides* | *Juncus scheuchzerioides* | OTU28 | B04 | 21E65 | 22 | 47 | 97980 | 0.00022 | 0.00048 |
| 21M30 | OTU10 | A06 | *Juncus scheuchzerioides* | *Juncus scheuchzerioides* | OTU28 | B04 | 21F33 | 27 | 44 | 93054 | 0.00029 | 0.00047 |
| 21M30 | OTU10 | A06 | *Juncus scheuchzerioides* | *Juncus scheuchzerioides* | OTU28 | B04 | 21F34 | 33 | 69 | 92994 | 0.00035 | 0.00074 |
| 21M30 | OTU10 | A06 | *Juncus scheuchzerioides* | *Juncus scheuchzerioides* | OTU29 | B08 | 21E53 | 22 | 70 | 92502 | 0.00024 | 0.00076 |
| 21M30 | OTU10 | A06 | *Juncus scheuchzerioides* | *Juncus scheuchzerioides* | OTU29 | B08 | 21E54 | 28 | 55 | 79388 | 0.00035 | 0.00069 |
| 21M30 | OTU10 | A06 | *Juncus scheuchzerioides* | *Deschampsia* | OTU05 | A04 | 21E05 | 119 | 255 | 91291 | 0.00130 | 0.00279 |
| 21M30 | OTU10 | A06 | *Juncus scheuchzerioides* | *Deschampsia* | OTU29 | A21 | 21E29 | 137 | 273 | 89286 | 0.00153 | 0.00306 |
| 21M30 | OTU10 | A06 | *Juncus scheuchzerioides* | *Deschampsia* | OTU35 | B16 | 21E81 | 140 | 280 | 82266 | 0.00170 | 0.00340 |
| 21M31 | OTU10 | A06 | *Juncus scheuchzerioides* | *Deschampsia* | OTU09 | A06 | 21D49 | 81 | 142 | 42831 | 0.00189 | 0.00332 |
| 21M31 | OTU10 | A06 | *Juncus scheuchzerioides* | *Deschampsia* | OTU09 | A06 | 21F52 | 49 | 92 | 13846 | 0.00354 | 0.00664 |
| 21M31 | OTU10 | A06 | *Juncus scheuchzerioides* | *Juncus scheuchzerioides* | OTU10 | A06 | 21D53 | 7 | 102 | 44174 | 0.00016 | 0.00231 |
| 21M31 | OTU10 | A06 | *Juncus scheuchzerioides* | *Juncus scheuchzerioides* | OTU10 | A06 | 21F53 | 8 | 115 | 30170 | 0.00027 | 0.00381 |
| 21M31 | OTU10 | A06 | *Juncus scheuchzerioides* | *Juncus scheuchzerioides* | OTU28 | B04 | 21E65 | 29 | 43 | 97980 | 0.00030 | 0.00044 |
| 21M31 | OTU10 | A06 | *Juncus scheuchzerioides* | *Juncus scheuchzerioides* | OTU28 | B04 | 21F33 | 31 | 37 | 93054 | 0.00033 | 0.00040 |
| 21M31 | OTU10 | A06 | *Juncus scheuchzerioides* | *Juncus scheuchzerioides* | OTU28 | B04 | 21F34 | 25 | 71 | 92994 | 0.00027 | 0.00076 |
| 21M31 | OTU10 | A06 | *Juncus scheuchzerioides* | *Juncus scheuchzerioides* | OTU29 | B08 | 21E53 | 26 | 82 | 92502 | 0.00028 | 0.00089 |
| 21M31 | OTU10 | A06 | *Juncus scheuchzerioides* | *Juncus scheuchzerioides* | OTU29 | B08 | 21E54 | 30 | 52 | 79388 | 0.00038 | 0.00066 |
| 21M31 | OTU10 | A06 | *Juncus scheuchzerioides* | *Deschampsia* | OTU05 | A04 | 21E05 | 133 | 208 | 91291 | 0.00146 | 0.00228 |
| 21M31 | OTU10 | A06 | *Juncus scheuchzerioides* | *Deschampsia* | OTU29 | A21 | 21E29 | 131 | 213 | 89286 | 0.00147 | 0.00239 |
| 21M31 | OTU10 | A06 | *Juncus scheuchzerioides* | *Deschampsia* | OTU35 | B16 | 21E81 | 140 | 250 | 82266 | 0.00170 | 0.00304 |
| 21M32 | OTU10 | A06 | *Juncus scheuchzerioides* | *Deschampsia* | OTU09 | A06 | 21D49 | 67 | 127 | 42831 | 0.00156 | 0.00297 |
| 21M32 | OTU10 | A06 | *Juncus scheuchzerioides* | *Deschampsia* | OTU09 | A06 | 21F52 | 40 | 76 | 13846 | 0.00289 | 0.00549 |
| 21M32 | OTU10 | A06 | *Juncus scheuchzerioides* | *Juncus scheuchzerioides* | OTU10 | A06 | 21D53 | 9 | 91 | 44174 | 0.00020 | 0.00206 |
| 21M32 | OTU10 | A06 | *Juncus scheuchzerioides* | *Juncus scheuchzerioides* | OTU10 | A06 | 21F53 | 8 | 106 | 30170 | 0.00027 | 0.00351 |
| 21M32 | OTU10 | A06 | *Juncus scheuchzerioides* | *Juncus scheuchzerioides* | OTU28 | B04 | 21E65 | 21 | 54 | 97980 | 0.00021 | 0.00055 |
| 21M32 | OTU10 | A06 | *Juncus scheuchzerioides* | *Juncus scheuchzerioides* | OTU28 | B04 | 21F33 | 36 | 47 | 93054 | 0.00039 | 0.00051 |
| 21M32 | OTU10 | A06 | *Juncus scheuchzerioides* | *Juncus scheuchzerioides* | OTU28 | B04 | 21F34 | 25 | 59 | 92994 | 0.00027 | 0.00063 |
| 21M32 | OTU10 | A06 | *Juncus scheuchzerioides* | *Juncus scheuchzerioides* | OTU29 | B08 | 21E53 | 28 | 91 | 92502 | 0.00030 | 0.00098 |
| 21M32 | OTU10 | A06 | *Juncus scheuchzerioides* | *Juncus scheuchzerioides* | OTU29 | B08 | 21E54 | 22 | 49 | 79388 | 0.00028 | 0.00062 |
| 21M32 | OTU10 | A06 | *Juncus scheuchzerioides* | *Deschampsia* | OTU05 | A04 | 21E05 | 128 | 183 | 91291 | 0.00140 | 0.00200 |
| 21M32 | OTU10 | A06 | *Juncus scheuchzerioides* | *Deschampsia* | OTU29 | A21 | 21E29 | 125 | 185 | 89286 | 0.00140 | 0.00207 |
| 21M32 | OTU10 | A06 | *Juncus scheuchzerioides* | *Deschampsia* | OTU35 | B16 | 21E81 | 130 | 190 | 82266 | 0.00158 | 0.00231 |
| 21M33 | OTU01 | B02 | *Cerastium fontanum* | *Cerastium fontanum* | OTU01 | B02 | 21E57 | 0 | 0 | 10012 | 0.00000 | 0.00000 |
| 21M33 | OTU01 | B02 | *Cerastium fontanum* | *Poa annua* | OTU08 | B02 | 21E56 | 18 | 41 | 19407 | 0.00093 | 0.00211 |
| 21M33 | OTU01 | B02 | *Cerastium fontanum* | *Ranunculus biternatus* | OTU39 | B02 | 21E59 | 51 | 121 | 123293 | 0.00041 | 0.00098 |
| 21M33 | OTU01 | B02 | *Cerastium fontanum* | *Taraxacum officinale* | OTU41 | B02 | 21E58 | 60 | 200 | 149501 | 0.00040 | 0.00134 |
| 21M33 | OTU01 | B02 | *Cerastium fontanum* | *Taraxacum officinale* | OTU41 | B02 | 21F24 | 62 | 185 | 151363 | 0.00041 | 0.00122 |
| 21M33 | OTU01 | B02 | *Cerastium fontanum* | *Taraxacum officinale* | OTU41 | B02 | 21F25 | 64 | 190 | 114094 | 0.00056 | 0.00167 |
| 21M34 | OTU01 | A16 | *Cerastium fontanum* | *Cerastium fontanum* | OTU01 | A16 | 21E28 | 0 | 11 | 8899 | 0.00000 | 0.00124 |
| 21M34 | OTU01 | A16 | *Cerastium fontanum* | *Scorzoneroides autumnalis* | OTU20 | A16 | 21D52 | 30 | 54 | 147557 | 0.00020 | 0.00037 |
| 21M35 | OTU01 | A16 | *Cerastium fontanum* | *Cerastium fontanum* | OTU01 | A16 | 21E28 | 0 | 0 | 8899 | 0.00000 | 0.00000 |
| 21M35 | OTU01 | A16 | *Cerastium fontanum* | *Scorzoneroides autumnalis* | OTU20 | A16 | 21D52 | 67 | 103 | 147557 | 0.00045 | 0.00070 |
| 21M36 | OTU01 | B11 | *Cerastium fontanum* | *Cerastium fontanum* | OTU01 | B11 | 21E68 | 1 | 2 | 70163 | 0.00001 | 0.00003 |
| 21M36 | OTU01 | B11 | *Cerastium fontanum* | *Poa annua* | OTU08 | B11 | 21E69 | 48 | 75 | 97950 | 0.00049 | 0.00077 |
| 21M36 | OTU01 | B11 | *Cerastium fontanum* | *Poa annua* | OTU08 | B11 | 21F30 | 36 | 58 | 23093 | 0.00156 | 0.00251 |
| 21M36 | OTU01 | B11 | *Cerastium fontanum* | *Poa annua* | OTU08 | B11 | 21F31 | 29 | 54 | 20776 | 0.00140 | 0.00260 |
| 21M37 | OTU08 | B11 | *Poa annua* | *Cerastium fontanum* | OTU01 | B11 | 21E68 | 85 | 173 | 70163 | 0.00121 | 0.00247 |
| 21M37 | OTU08 | B11 | *Poa annua* | *Poa annua* | OTU08 | B11 | 21E69 | 2 | 2 | 97950 | 0.00002 | 0.00002 |
| 21M37 | OTU08 | B11 | *Poa annua* | *Poa annua* | OTU08 | B11 | 21F30 | 2 | 2 | 23093 | 0.00009 | 0.00009 |
| 21M37 | OTU08 | B11 | *Poa annua* | *Poa annua* | OTU08 | B11 | 21F31 | 2 | 2 | 20776 | 0.00010 | 0.00010 |
| 21M38 | OTU08 | B11 | *Poa annua* | *Cerastium fontanum* | OTU01 | B11 | 21E68 | 95 | 197 | 70163 | 0.00135 | 0.00281 |
| 21M38 | OTU08 | B11 | *Poa annua* | *Poa annua* | OTU08 | B11 | 21E69 | 1 | 2 | 97950 | 0.00001 | 0.00002 |
| 21M38 | OTU08 | B11 | *Poa annua* | *Poa annua* | OTU08 | B11 | 21F30 | 1 | 2 | 23093 | 0.00004 | 0.00009 |
| 21M38 | OTU08 | B11 | *Poa annua* | *Poa annua* | OTU08 | B11 | 21F31 | 1 | 2 | 20776 | 0.00005 | 0.00010 |
| 21M39 | OTU08 | B11 | *Poa annua* | *Cerastium fontanum* | OTU01 | B11 | 21E68 | 119 | 249 | 70163 | 0.00170 | 0.00355 |
| 21M39 | OTU08 | B11 | *Poa annua* | *Poa annua* | OTU08 | B11 | 21E69 | 1 | 3 | 97950 | 0.00001 | 0.00003 |
| 21M39 | OTU08 | B11 | *Poa annua* | *Poa annua* | OTU08 | B11 | 21F30 | 1 | 2 | 23093 | 0.00004 | 0.00009 |
| 21M39 | OTU08 | B11 | *Poa annua* | *Poa annua* | OTU08 | B11 | 21F31 | 1 | 4 | 20776 | 0.00005 | 0.00019 |
| 21M40 | OTU08 | B11 | *Poa annua* | *Cerastium fontanum* | OTU01 | B11 | 21E68 | 85 | 157 | 70163 | 0.00121 | 0.00224 |
| 21M40 | OTU08 | B11 | *Poa annua* | *Poa annua* | OTU08 | B11 | 21E69 | 3 | 4 | 97950 | 0.00003 | 0.00004 |
| 21M40 | OTU08 | B11 | *Poa annua* | *Poa annua* | OTU08 | B11 | 21F30 | 3 | 11 | 23093 | 0.00013 | 0.00048 |
| 21M40 | OTU08 | B11 | *Poa annua* | *Poa annua* | OTU08 | B11 | 21F31 | 3 | 11 | 20776 | 0.00014 | 0.00053 |
| 21M41 | OTU08 | B11 | *Poa annua* | *Cerastium fontanum* | OTU01 | B11 | 21E68 | 124 | 313 | 70163 | 0.00177 | 0.00446 |
| 21M41 | OTU08 | B11 | *Poa annua* | *Poa annua* | OTU08 | B11 | 21E69 | 3 | 39 | 97950 | 0.00003 | 0.00040 |
| 21M41 | OTU08 | B11 | *Poa annua* | *Poa annua* | OTU08 | B11 | 21F30 | 3 | 39 | 23093 | 0.00013 | 0.00169 |
| 21M41 | OTU08 | B11 | *Poa annua* | *Poa annua* | OTU08 | B11 | 21F31 | 3 | 39 | 20776 | 0.00014 | 0.00188 |
| 21M42 | OTU01 | A05 | *Cerastium fontanum* | *Acaena magellanica* | OTU07 | A05 | 21D48 | 66 | 143 | 94784 | 0.00070 | 0.00151 |
| 21M42 | OTU01 | A05 | *Cerastium fontanum* | *Acaena magellanica* | OTU07 | A05 | 21E45 | 53 | 86 | 34525 | 0.00154 | 0.00249 |
| 21M42 | OTU01 | A05 | *Cerastium fontanum* | *Cerastium fontanum* | OTU01 | A05 | 21E08 | 9 | 12 | 115955 | 0.00008 | 0.00010 |
| 21M42 | OTU01 | A05 | *Cerastium fontanum* | *Cerastium fontanum* | OTU01 | A05 | 21F36 | 4 | 9 | 148121 | 0.00003 | 0.00006 |
| 21M42 | OTU01 | A05 | *Cerastium fontanum* | *Cerastium fontanum* | OTU01 | A05 | 21F54 | 4 | 4 | 35623 | 0.00011 | 0.00011 |
| 21M42 | OTU01 | A05 | *Cerastium fontanum* | *Cerastium fontanum* | OTU01 | A05 | 21F55 | 0 | 0 | 45048 | 0.00000 | 0.00000 |
| 21M42 | OTU01 | A05 | *Cerastium fontanum* | *Cerastium fontanum* | OTU01 | A05 | 21F56 | 7 | 12 | 147039 | 0.00005 | 0.00008 |
| 21M42 | OTU01 | A05 | *Cerastium fontanum* | *Poa annua* | OTU04 | A05 | 21E06 | 18 | 56 | 20695 | 0.00087 | 0.00271 |
| 21M42 | OTU01 | A05 | *Cerastium fontanum* | *Poa annua* | OTU08 | A05 | 21E46 | 55 | 114 | 80926 | 0.00068 | 0.00141 |
| 21M42 | OTU01 | A05 | *Cerastium fontanum* | *Taraxacum officinale* | OTU06 | A05 | 21E07 | 64 | 146 | 108195 | 0.00059 | 0.00135 |
| 21M43 | OTU01 | A05 | *Cerastium fontanum* | *Acaena magellanica* | OTU07 | A05 | 21D48 | 77 | 127 | 94784 | 0.00081 | 0.00134 |
| 21M43 | OTU01 | A05 | *Cerastium fontanum* | *Acaena magellanica* | OTU07 | A05 | 21E45 | 47 | 77 | 34525 | 0.00136 | 0.00223 |
| 21M43 | OTU01 | A05 | *Cerastium fontanum* | *Cerastium fontanum* | OTU01 | A05 | 21E08 | 0 | 7 | 115955 | 0.00000 | 0.00006 |
| 21M43 | OTU01 | A05 | *Cerastium fontanum* | *Cerastium fontanum* | OTU01 | A05 | 21F36 | 0 | 7 | 148121 | 0.00000 | 0.00005 |
| 21M43 | OTU01 | A05 | *Cerastium fontanum* | *Cerastium fontanum* | OTU01 | A05 | 21F54 | 0 | 0 | 35623 | 0.00000 | 0.00000 |
| 21M43 | OTU01 | A05 | *Cerastium fontanum* | *Cerastium fontanum* | OTU01 | A05 | 21F55 | 0 | 0 | 45048 | 0.00000 | 0.00000 |
| 21M43 | OTU01 | A05 | *Cerastium fontanum* | *Cerastium fontanum* | OTU01 | A05 | 21F56 | 0 | 7 | 147039 | 0.00000 | 0.00005 |
| 21M43 | OTU01 | A05 | *Cerastium fontanum* | *Poa annua* | OTU04 | A05 | 21E06 | 18 | 54 | 20695 | 0.00087 | 0.00261 |
| 21M43 | OTU01 | A05 | *Cerastium fontanum* | *Poa annua* | OTU08 | A05 | 21E46 | 40 | 106 | 80926 | 0.00049 | 0.00131 |
| 21M43 | OTU01 | A05 | *Cerastium fontanum* | *Taraxacum officinale* | OTU06 | A05 | 21E07 | 59 | 143 | 108195 | 0.00055 | 0.00132 |
| 21M44 | OTU19 | A15 | *Ranunculus biternatus* | *Cerastium fontanum* | OTU01 | A15 | 21E21 | 4 | 8 | 8479 | 0.00047 | 0.00094 |
| 21M44 | OTU19 | A15 | *Ranunculus biternatus* | *Phleum alpinum* | OTU18 | A15 | 21E23 | 58 | 113 | 96370 | 0.00060 | 0.00117 |
| 21M44 | OTU19 | A15 | *Ranunculus biternatus* | *Phleum alpinum* | OTU18 | A15 | 21E43 | 58 | 114 | 96317 | 0.00060 | 0.00118 |
| 21M44 | OTU19 | A15 | *Ranunculus biternatus* | *Poa annua* | OTU04 | A15 | 21E22 | 29 | 63 | 24693 | 0.00117 | 0.00255 |
| 21M44 | OTU19 | A15 | *Ranunculus biternatus* | *Ranunculus biternatus* | OTU19 | A15 | 21E40 | 1 | 15 | 8884 | 0.00011 | 0.00169 |
| 21M44 | OTU19 | A15 | *Ranunculus biternatus* | *Ranunculus biternatus* | OTU19 | A15 | 21F45 | 5 | 29 | 26570 | 0.00019 | 0.00109 |
| 21M44 | OTU19 | A15 | *Ranunculus biternatus* | *Cerastium fontanum* | OTU01 | A05 | 21F36 | 300 | 789 | 148121 | 0.00203 | 0.00533 |
| 21M45 | OTU04 | A21 | *Poa annua* | *Poa annua* | OTU04 | A21 | 21F42 | 1 | 2 | 21487 | 0.00005 | 0.00009 |
| 21M45 | OTU04 | A21 | *Poa annua* | *Poa annua* | OTU04 | A21 | 21E32 | 1 | 2 | 22621 | 0.00004 | 0.00009 |
| 21M45 | OTU04 | A21 | *Poa annua* | *Poa annua* | OTU04 | A21 | 21F43 | 1 | 9 | 66812 | 0.00001 | 0.00013 |
| 21M45 | OTU04 | A21 | *Poa annua* | *Poa annua* | OTU22 | A21 | 21E33 | 2 | 22 | 99269 | 0.00002 | 0.00022 |
| 21M46 | OTU04 | A21 | *Poa annua* | *Poa annua* | OTU04 | A21 | 21F42 | 2 | 2 | 21487 | 0.00009 | 0.00009 |
| 21M46 | OTU04 | A21 | *Poa annua* | *Poa annua* | OTU04 | A21 | 21E32 | 2 | 2 | 22621 | 0.00009 | 0.00009 |
| 21M46 | OTU04 | A21 | *Poa annua* | *Poa annua* | OTU04 | A21 | 21F43 | 2 | 6 | 66812 | 0.00003 | 0.00009 |
| 21M46 | OTU04 | A21 | *Poa annua* | *Poa annua* | OTU22 | A21 | 21E33 | 3 | 13 | 99269 | 0.00003 | 0.00013 |
| 21M47 | OTU04 | A21 | *Poa annua* | *Poa annua* | OTU04 | A21 | 21F42 | 2 | 2 | 21487 | 0.00009 | 0.00009 |
| 21M47 | OTU04 | A21 | *Poa annua* | *Poa annua* | OTU04 | A21 | 21E32 | 2 | 2 | 22621 | 0.00009 | 0.00009 |
| 21M47 | OTU04 | A21 | *Poa annua* | *Poa annua* | OTU04 | A21 | 21F43 | 4 | 8 | 66812 | 0.00006 | 0.00012 |
| 21M47 | OTU04 | A21 | *Poa annua* | *Poa annua* | OTU22 | A21 | 21E33 | 2 | 11 | 99269 | 0.00002 | 0.00011 |
| 21M48 | OTU04 | A21 | *Poa annua* | *Poa annua* | OTU04 | A21 | 21F42 | 2 | 2 | 21487 | 0.00009 | 0.00009 |
| 21M48 | OTU04 | A21 | *Poa annua* | *Poa annua* | OTU04 | A21 | 21E32 | 2 | 2 | 22621 | 0.00009 | 0.00009 |
| 21M48 | OTU04 | A21 | *Poa annua* | *Poa annua* | OTU04 | A21 | 21F43 | 5 | 24 | 66812 | 0.00007 | 0.00036 |
| 21M48 | OTU04 | A21 | *Poa annua* | *Poa annua* | OTU22 | A21 | 21E33 | 3 | 34 | 99269 | 0.00003 | 0.00034 |
| 21M49 | OTU04 | A21 | *Poa annua* | *Poa annua* | OTU04 | A21 | 21F42 | 1 | 3 | 21487 | 0.00005 | 0.00014 |
| 21M49 | OTU04 | A21 | *Poa annua* | *Poa annua* | OTU04 | A21 | 21E32 | 1 | 3 | 22621 | 0.00004 | 0.00013 |
| 21M49 | OTU04 | A21 | *Poa annua* | *Poa annua* | OTU04 | A21 | 21F43 | 2 | 15 | 66812 | 0.00003 | 0.00022 |
| 21M49 | OTU04 | A21 | *Poa annua* | *Poa annua* | OTU22 | A21 | 21E33 | 4 | 29 | 99269 | 0.00004 | 0.00029 |
| 21M50 | OTU04 | A21 | *Poa annua* | *Poa annua* | OTU04 | A21 | 21F42 | 1 | 2 | 21487 | 0.00005 | 0.00009 |
| 21M50 | OTU04 | A21 | *Poa annua* | *Poa annua* | OTU04 | A21 | 21E32 | 1 | 2 | 22621 | 0.00004 | 0.00009 |
| 21M50 | OTU04 | A21 | *Poa annua* | *Poa annua* | OTU04 | A21 | 21F43 | 2 | 15 | 66812 | 0.00003 | 0.00022 |
| 21M50 | OTU04 | A21 | *Poa annua* | *Poa annua* | OTU22 | A21 | 21E33 | 1 | 31 | 99269 | 0.00001 | 0.00031 |
| 21M51 | OTU08 | B10 | *Poa annua* | *Poa annua* | OTU08 | B10 | 21D54 | 14 | 47 | 62527 | 0.00022 | 0.00075 |
| 21M51 | OTU08 | B10 | *Poa annua* | *Poa annua* | OTU08 | B10 | 21E67 | 0 | 0 | 19232 | 0.00000 | 0.00000 |
| 21M52 | OTU08 | B13 | *Poa annua* | *Cerastium fontanum* | OTU01 | B13 | 21E74 | 109 | 234 | 66393 | 0.00164 | 0.00352 |
| 21M52 | OTU08 | B13 | *Poa annua* | *Poa annua* | OTU08 | B13 | 21E76 | 2 | 2 | 14638 | 0.00014 | 0.00014 |
| 21M52 | OTU08 | B13 | *Poa annua* | *Phleum alpinum* | OTU30 | B13 | 21E73 | 9 | 19 | 21368 | 0.00042 | 0.00089 |
| 21M52 | OTU08 | B13 | *Poa annua* | *Ranunculus biternatus* | OTU38 | B13 | 21E72 | 85 | 110 | 132527 | 0.00064 | 0.00083 |
| 21M52 | OTU08 | B13 | *Poa annua* | *Festuca contracta* | OTU43 | B13 | 21E75 | 7 | 10 | 13110 | 0.00053 | 0.00076 |
| 21M52 | OTU08 | B13 | *Poa annua* | *Festuca contracta* | OTU34 | B06 | 21E82 | 109 | 276 | 107720 | 0.00101 | 0.00256 |
| 21M52 | OTU08 | B13 | *Poa annua* | *Ranunculus biternatus* | OTU19 | A15 | 21F45 | 90 | 102 | 26570 | 0.00339 | 0.00384 |
| 21M52 | OTU08 | B13 | *Poa annua* | *Phleum alpinum* | OTU25 | A09 | 21E14 | 124 | 274 | 111946 | 0.00111 | 0.00245 |
| 21M52 | OTU08 | B13 | *Poa annua* | *Cerastium fontanum* | OTU01 | A05 | 21F36 | 201 | 294 | 148121 | 0.00136 | 0.00198 |
| 21M53 | OTU01 | C05 | *Cerastium fontanum* | *Cerastium fontanum* | OTU01 | C05 | 21E94 | 0 | 6 | 1987 | 0.00000 | 0.00302 |
| 21M53 | OTU01 | C05 | *Cerastium fontanum* | *Cerastium fontanum* | OTU01 | C05 | 21E95 | 0 | 1 | 9324 | 0.00000 | 0.00011 |
| 21M53 | OTU01 | C05 | *Cerastium fontanum* | *Cerastium fontanum* | OTU01 | C11 | 21F13 | 0 | 0 | 148673 | 0.00000 | 0.00000 |
| 21M53 | OTU01 | C05 | *Cerastium fontanum* | *Cerastium fontanum* | OTU01 | A11 | 21E17 | 0 | 0 | 148662 | 0.00000 | 0.00000 |
| 21M54 | OTU01 | C05 | *Cerastium fontanum* | *Cerastium fontanum* | OTU01 | C05 | 21E94 | 0 | 0 | 1987 | 0.00000 | 0.00000 |
| 21M54 | OTU01 | C05 | *Cerastium fontanum* | *Cerastium fontanum* | OTU01 | C05 | 21E95 | 0 | 0 | 9324 | 0.00000 | 0.00000 |
| 21M54 | OTU01 | C05 | *Cerastium fontanum* | *Cerastium fontanum* | OTU01 | C11 | 21F13 | 0 | 0 | 148673 | 0.00000 | 0.00000 |
| 21M54 | OTU01 | C05 | *Cerastium fontanum* | *Cerastium fontanum* | OTU01 | A11 | 21E17 | 0 | 0 | 148662 | 0.00000 | 0.00000 |
| 21M55 | OTU01 | A05 | *Cerastium fontanum* | *Cerastium fontanum* | OTU01 | A05 | 21F55 | 1 | 1 | 45048 | 0.00002 | 0.00002 |
| 21M55 | OTU01 | A05 | *Cerastium fontanum* | *Cerastium fontanum* | OTU01 | A05 | 21E08 | 2 | 10 | 115955 | 0.00002 | 0.00009 |
| 21M55 | OTU01 | A05 | *Cerastium fontanum* | *Cerastium fontanum* | OTU01 | A05 | 21F36 | 1 | 10 | 148121 | 0.00001 | 0.00007 |
| 21M55 | OTU01 | A05 | *Cerastium fontanum* | *Cerastium fontanum* | OTU01 | A05 | 21F54 | 0 | 0 | 35623 | 0.00000 | 0.00000 |
| 21M55 | OTU01 | A05 | *Cerastium fontanum* | *Cerastium fontanum* | OTU01 | A05 | 21F56 | 1 | 10 | 147039 | 0.00001 | 0.00007 |
| 21M55 | OTU01 | A05 | *Cerastium fontanum* | *Acaena magellanica* | OTU07 | A05 | 21D48 | 90 | 151 | 94784 | 0.00095 | 0.00159 |
| 21M55 | OTU01 | A05 | *Cerastium fontanum* | *Poa annua* | OTU04 | A05 | 21E06 | 33 | 71 | 20695 | 0.00159 | 0.00343 |
| 21M55 | OTU01 | A05 | *Cerastium fontanum* | *Poa annua* | OTU08 | A05 | 21E46 | 65 | 153 | 80926 | 0.00080 | 0.00189 |
| 21M55 | OTU01 | A05 | *Cerastium fontanum* | *Taraxacum officinale* | OTU06 | A05 | 21E07 | 98 | 202 | 108195 | 0.00091 | 0.00187 |
| 21M55 | OTU01 | A05 | *Cerastium fontanum* | *Acaena magellanica* | OTU07 | A05 | 21E45 | 51 | 72 | 34525 | 0.00148 | 0.00209 |
| 21M56 | OTU01 | A05 | *Cerastium fontanum* | *Cerastium fontanum* | OTU01 | A05 | 21F55 | 0 | 0 | 45048 | 0.00000 | 0.00000 |
| 21M56 | OTU01 | A05 | *Cerastium fontanum* | *Cerastium fontanum* | OTU01 | A05 | 21E08 | 9 | 15 | 115955 | 0.00008 | 0.00013 |
| 21M56 | OTU01 | A05 | *Cerastium fontanum* | *Cerastium fontanum* | OTU01 | A05 | 21F36 | 4 | 11 | 148121 | 0.00003 | 0.00007 |
| 21M56 | OTU01 | A05 | *Cerastium fontanum* | *Cerastium fontanum* | OTU01 | A05 | 21F54 | 1 | 4 | 35623 | 0.00003 | 0.00011 |
| 21M56 | OTU01 | A05 | *Cerastium fontanum* | *Cerastium fontanum* | OTU01 | A05 | 21F56 | 11 | 18 | 147039 | 0.00007 | 0.00012 |
| 21M56 | OTU01 | A05 | *Cerastium fontanum* | *Acaena magellanica* | OTU07 | A05 | 21D48 | 73 | 133 | 94784 | 0.00077 | 0.00140 |
| 21M56 | OTU01 | A05 | *Cerastium fontanum* | *Poa annua* | OTU04 | A05 | 21E06 | 26 | 58 | 20695 | 0.00126 | 0.00280 |
| 21M56 | OTU01 | A05 | *Cerastium fontanum* | *Poa annua* | OTU08 | A05 | 21E46 | 49 | 133 | 80926 | 0.00061 | 0.00164 |
| 21M56 | OTU01 | A05 | *Cerastium fontanum* | *Taraxacum officinale* | OTU06 | A05 | 21E07 | 75 | 176 | 108195 | 0.00069 | 0.00163 |
| 21M56 | OTU01 | A05 | *Cerastium fontanum* | *Acaena magellanica* | OTU07 | A05 | 21E45 | 48 | 59 | 34525 | 0.00139 | 0.00171 |
| 21M57 | OTU01 | A05 | *Cerastium fontanum* | *Cerastium fontanum* | OTU01 | A05 | 21F55 | 0 | 1 | 45048 | 0.00000 | 0.00002 |
| 21M57 | OTU01 | A05 | *Cerastium fontanum* | *Cerastium fontanum* | OTU01 | A05 | 21E08 | 0 | 10 | 115955 | 0.00000 | 0.00009 |
| 21M57 | OTU01 | A05 | *Cerastium fontanum* | *Cerastium fontanum* | OTU01 | A05 | 21F36 | 1 | 10 | 148121 | 0.00001 | 0.00007 |
| 21M57 | OTU01 | A05 | *Cerastium fontanum* | *Cerastium fontanum* | OTU01 | A05 | 21F54 | 0 | 0 | 35623 | 0.00000 | 0.00000 |
| 21M57 | OTU01 | A05 | *Cerastium fontanum* | *Cerastium fontanum* | OTU01 | A05 | 21F56 | 1 | 10 | 147039 | 0.00001 | 0.00007 |
| 21M57 | OTU01 | A05 | *Cerastium fontanum* | *Acaena magellanica* | OTU07 | A05 | 21D48 | 91 | 162 | 94784 | 0.00096 | 0.00171 |
| 21M57 | OTU01 | A05 | *Cerastium fontanum* | *Poa annua* | OTU04 | A05 | 21E06 | 32 | 72 | 20695 | 0.00155 | 0.00348 |
| 21M57 | OTU01 | A05 | *Cerastium fontanum* | *Poa annua* | OTU08 | A05 | 21E46 | 58 | 180 | 80926 | 0.00072 | 0.00222 |
| 21M57 | OTU01 | A05 | *Cerastium fontanum* | *Taraxacum officinale* | OTU06 | A05 | 21E07 | 71 | 171 | 108195 | 0.00066 | 0.00158 |
| 21M57 | OTU01 | A05 | *Cerastium fontanum* | *Acaena magellanica* | OTU07 | A05 | 21E45 | 42 | 80 | 34525 | 0.00122 | 0.00232 |
| 21M58 | OTU01 | A05 | *Cerastium fontanum* | *Cerastium fontanum* | OTU01 | A05 | 21F55 | 0 | 19 | 45048 | 0.00000 | 0.00042 |
| 21M58 | OTU01 | A05 | *Cerastium fontanum* | *Cerastium fontanum* | OTU01 | A05 | 21E08 | 3 | 51 | 115955 | 0.00003 | 0.00044 |
| 21M58 | OTU01 | A05 | *Cerastium fontanum* | *Cerastium fontanum* | OTU01 | A05 | 21F36 | 4 | 33 | 148121 | 0.00003 | 0.00022 |
| 21M58 | OTU01 | A05 | *Cerastium fontanum* | *Cerastium fontanum* | OTU01 | A05 | 21F54 | 0 | 18 | 35623 | 0.00000 | 0.00051 |
| 21M58 | OTU01 | A05 | *Cerastium fontanum* | *Cerastium fontanum* | OTU01 | A05 | 21F56 | 10 | 33 | 147039 | 0.00007 | 0.00022 |
| 21M58 | OTU01 | A05 | *Cerastium fontanum* | *Acaena magellanica* | OTU07 | A05 | 21D48 | 67 | 156 | 94784 | 0.00071 | 0.00165 |
| 21M58 | OTU01 | A05 | *Cerastium fontanum* | *Poa annua* | OTU04 | A05 | 21E06 | 11 | 55 | 20695 | 0.00053 | 0.00266 |
| 21M58 | OTU01 | A05 | *Cerastium fontanum* | *Poa annua* | OTU08 | A05 | 21E46 | 41 | 141 | 80926 | 0.00051 | 0.00174 |
| 21M58 | OTU01 | A05 | *Cerastium fontanum* | *Taraxacum officinale* | OTU06 | A05 | 21E07 | 67 | 204 | 108195 | 0.00062 | 0.00189 |
| 21M58 | OTU01 | A05 | *Cerastium fontanum* | *Acaena magellanica* | OTU07 | A05 | 21E45 | 41 | 90 | 34525 | 0.00119 | 0.00261 |
| 21M59 | OTU01 | A05 | *Cerastium fontanum* | *Cerastium fontanum* | OTU01 | A05 | 21F55 | 0 | 0 | 45048 | 0.00000 | 0.00000 |
| 21M59 | OTU01 | A05 | *Cerastium fontanum* | *Cerastium fontanum* | OTU01 | A05 | 21E08 | 0 | 9 | 115955 | 0.00000 | 0.00008 |
| 21M59 | OTU01 | A05 | *Cerastium fontanum* | *Cerastium fontanum* | OTU01 | A05 | 21F36 | 0 | 10 | 148121 | 0.00000 | 0.00007 |
| 21M59 | OTU01 | A05 | *Cerastium fontanum* | *Cerastium fontanum* | OTU01 | A05 | 21F54 | 0 | 0 | 35623 | 0.00000 | 0.00000 |
| 21M59 | OTU01 | A05 | *Cerastium fontanum* | *Cerastium fontanum* | OTU01 | A05 | 21F56 | 0 | 15 | 147039 | 0.00000 | 0.00010 |
| 21M59 | OTU01 | A05 | *Cerastium fontanum* | *Acaena magellanica* | OTU07 | A05 | 21D48 | 86 | 148 | 94784 | 0.00091 | 0.00156 |
| 21M59 | OTU01 | A05 | *Cerastium fontanum* | *Poa annua* | OTU04 | A05 | 21E06 | 31 | 68 | 20695 | 0.00150 | 0.00329 |
| 21M59 | OTU01 | A05 | *Cerastium fontanum* | *Poa annua* | OTU08 | A05 | 21E46 | 64 | 165 | 80926 | 0.00079 | 0.00204 |
| 21M59 | OTU01 | A05 | *Cerastium fontanum* | *Taraxacum officinale* | OTU06 | A05 | 21E07 | 81 | 171 | 108195 | 0.00075 | 0.00158 |
| 21M59 | OTU01 | A05 | *Cerastium fontanum* | *Acaena magellanica* | OTU07 | A05 | 21E45 | 43 | 81 | 34525 | 0.00125 | 0.00235 |
| 21M60 | OTU01 | A05 | *Cerastium fontanum* | *Cerastium fontanum* | OTU01 | A05 | 21F55 | 0 | 1 | 45048 | 0.00000 | 0.00002 |
| 21M60 | OTU01 | A05 | *Cerastium fontanum* | *Cerastium fontanum* | OTU01 | A05 | 21E08 | 0 | 9 | 115955 | 0.00000 | 0.00008 |
| 21M60 | OTU01 | A05 | *Cerastium fontanum* | *Cerastium fontanum* | OTU01 | A05 | 21F36 | 0 | 11 | 148121 | 0.00000 | 0.00007 |
| 21M60 | OTU01 | A05 | *Cerastium fontanum* | *Cerastium fontanum* | OTU01 | A05 | 21F54 | 0 | 0 | 35623 | 0.00000 | 0.00000 |
| 21M60 | OTU01 | A05 | *Cerastium fontanum* | *Cerastium fontanum* | OTU01 | A05 | 21F56 | 0 | 19 | 147039 | 0.00000 | 0.00013 |
| 21M60 | OTU01 | A05 | *Cerastium fontanum* | *Acaena magellanica* | OTU07 | A05 | 21D48 | 61 | 125 | 94784 | 0.00064 | 0.00132 |
| 21M60 | OTU01 | A05 | *Cerastium fontanum* | *Poa annua* | OTU04 | A05 | 21E06 | 19 | 39 | 20695 | 0.00092 | 0.00188 |
| 21M60 | OTU01 | A05 | *Cerastium fontanum* | *Poa annua* | OTU08 | A05 | 21E46 | 45 | 102 | 80926 | 0.00056 | 0.00126 |
| 21M60 | OTU01 | A05 | *Cerastium fontanum* | *Taraxacum officinale* | OTU06 | A05 | 21E07 | 76 | 157 | 108195 | 0.00070 | 0.00145 |
| 21M60 | OTU01 | A05 | *Cerastium fontanum* | *Acaena magellanica* | OTU07 | A05 | 21E45 | 36 | 71 | 34525 | 0.00104 | 0.00206 |
| 21M61 | OTU01 | A05 | *Cerastium fontanum* | *Cerastium fontanum* | OTU01 | A05 | 21F55 | 0 | 6 | 45048 | 0.00000 | 0.00013 |
| 21M61 | OTU01 | A05 | *Cerastium fontanum* | *Cerastium fontanum* | OTU01 | A05 | 21E08 | 4 | 16 | 115955 | 0.00003 | 0.00014 |
| 21M61 | OTU01 | A05 | *Cerastium fontanum* | *Cerastium fontanum* | OTU01 | A05 | 21F36 | 3 | 10 | 148121 | 0.00002 | 0.00007 |
| 21M61 | OTU01 | A05 | *Cerastium fontanum* | *Cerastium fontanum* | OTU01 | A05 | 21F54 | 0 | 6 | 35623 | 0.00000 | 0.00017 |
| 21M61 | OTU01 | A05 | *Cerastium fontanum* | *Cerastium fontanum* | OTU01 | A05 | 21F56 | 3 | 10 | 147039 | 0.00002 | 0.00007 |
| 21M61 | OTU01 | A05 | *Cerastium fontanum* | *Acaena magellanica* | OTU07 | A05 | 21D48 | 113 | 174 | 94784 | 0.00119 | 0.00184 |
| 21M61 | OTU01 | A05 | *Cerastium fontanum* | *Poa annua* | OTU04 | A05 | 21E06 | 17 | 48 | 20695 | 0.00082 | 0.00232 |
| 21M61 | OTU01 | A05 | *Cerastium fontanum* | *Poa annua* | OTU08 | A05 | 21E46 | 45 | 95 | 80926 | 0.00056 | 0.00117 |
| 21M61 | OTU01 | A05 | *Cerastium fontanum* | *Taraxacum officinale* | OTU06 | A05 | 21E07 | 82 | 187 | 108195 | 0.00076 | 0.00173 |
| 21M61 | OTU01 | A05 | *Cerastium fontanum* | *Acaena magellanica* | OTU07 | A05 | 21E45 | 33 | 82 | 34525 | 0.00096 | 0.00238 |
| 21M62 | OTU01 | A05 | *Cerastium fontanum* | *Cerastium fontanum* | OTU01 | A05 | 21F55 | 0 | 4 | 45048 | 0.00000 | 0.00009 |
| 21M62 | OTU01 | A05 | *Cerastium fontanum* | *Cerastium fontanum* | OTU01 | A05 | 21E08 | 1 | 17 | 115955 | 0.00001 | 0.00015 |
| 21M62 | OTU01 | A05 | *Cerastium fontanum* | *Cerastium fontanum* | OTU01 | A05 | 21F36 | 2 | 15 | 148121 | 0.00001 | 0.00010 |
| 21M62 | OTU01 | A05 | *Cerastium fontanum* | *Cerastium fontanum* | OTU01 | A05 | 21F54 | 0 | 4 | 35623 | 0.00000 | 0.00011 |
| 21M62 | OTU01 | A05 | *Cerastium fontanum* | *Cerastium fontanum* | OTU01 | A05 | 21F56 | 2 | 14 | 147039 | 0.00001 | 0.00010 |
| 21M62 | OTU01 | A05 | *Cerastium fontanum* | *Acaena magellanica* | OTU07 | A05 | 21D48 | 68 | 141 | 94784 | 0.00072 | 0.00149 |
| 21M62 | OTU01 | A05 | *Cerastium fontanum* | *Poa annua* | OTU04 | A05 | 21E06 | 38 | 73 | 20695 | 0.00184 | 0.00353 |
| 21M62 | OTU01 | A05 | *Cerastium fontanum* | *Poa annua* | OTU08 | A05 | 21E46 | 66 | 161 | 80926 | 0.00082 | 0.00199 |
| 21M62 | OTU01 | A05 | *Cerastium fontanum* | *Taraxacum officinale* | OTU06 | A05 | 21E07 | 79 | 172 | 108195 | 0.00073 | 0.00159 |
| 21M62 | OTU01 | A05 | *Cerastium fontanum* | *Acaena magellanica* | OTU07 | A05 | 21E45 | 39 | 68 | 34525 | 0.00113 | 0.00197 |
| 21M63 | OTU01 | A08 | *Cerastium fontanum* | *Cerastium fontanum* | OTU01 | A08 | 21E11 | 1 | 8 | 9766 | 0.00010 | 0.00082 |
| 21M63 | OTU01 | A08 | *Cerastium fontanum* | *Deschampsia* | OTU14 | A08 | 21E12 | 7 | 9 | 11434 | 0.00061 | 0.00079 |
| 21M63 | OTU01 | A08 | *Cerastium fontanum* | *Festuca contracta* | OTU15 | A08 | 21E13 | 66 | 191 | 98044 | 0.00067 | 0.00195 |
| 21M63 | OTU01 | A08 | *Cerastium fontanum* | *Festuca contracta* | OTU14 | A08 | 21E44 | 9 | 15 | 10412 | 0.00086 | 0.00144 |
| 21M63 | OTU01 | A08 | *Cerastium fontanum* | *Festuca contracta* | OTU15 | A08 | 21F47 | 15 | 17 | 14628 | 0.00103 | 0.00116 |
| 21M63 | OTU01 | A08 | *Cerastium fontanum* | *Festuca contracta* | OTU15 | A08 | 21F48 | 11 | 24 | 21224 | 0.00052 | 0.00113 |
| 21M63 | OTU01 | A08 | *Cerastium fontanum* | *Poa pratensis* | OTU13 | A08 | 21D51 | 30 | 73 | 68085 | 0.00044 | 0.00107 |
| 21M63 | OTU01 | A08 | *Cerastium fontanum* | *Poa pratensis* | OTU13 | A08 | 21E35 | 7 | 19 | 7870 | 0.00089 | 0.00241 |
| 21M63 | OTU01 | A08 | *Cerastium fontanum* | *Poa pratensis* | OTU13 | A08 | 21E48 | 10 | 25 | 10781 | 0.00093 | 0.00232 |
| 21M63 | OTU01 | A08 | *Cerastium fontanum* | *Deschampsia* | OTU05 | A04 | 21E05 | 58 | 162 | 91291 | 0.00064 | 0.00177 |
| 21M64 | OTU01 | A08 | *Cerastium fontanum* | *Cerastium fontanum* | OTU01 | A08 | 21E11 | 2 | 10 | 9766 | 0.00020 | 0.00102 |
| 21M64 | OTU01 | A08 | *Cerastium fontanum* | *Deschampsia* | OTU14 | A08 | 21E12 | 7 | 22 | 11434 | 0.00061 | 0.00192 |
| 21M64 | OTU01 | A08 | *Cerastium fontanum* | *Festuca contracta* | OTU15 | A08 | 21E13 | 92 | 241 | 98044 | 0.00094 | 0.00246 |
| 21M64 | OTU01 | A08 | *Cerastium fontanum* | *Festuca contracta* | OTU14 | A08 | 21E44 | 8 | 18 | 10412 | 0.00077 | 0.00173 |
| 21M64 | OTU01 | A08 | *Cerastium fontanum* | *Festuca contracta* | OTU15 | A08 | 21F47 | 9 | 16 | 14628 | 0.00062 | 0.00109 |
| 21M64 | OTU01 | A08 | *Cerastium fontanum* | *Festuca contracta* | OTU15 | A08 | 21F48 | 11 | 29 | 21224 | 0.00052 | 0.00137 |
| 21M64 | OTU01 | A08 | *Cerastium fontanum* | *Poa pratensis* | OTU13 | A08 | 21D51 | 31 | 127 | 68085 | 0.00046 | 0.00187 |
| 21M64 | OTU01 | A08 | *Cerastium fontanum* | *Poa pratensis* | OTU13 | A08 | 21E35 | 5 | 15 | 7870 | 0.00064 | 0.00191 |
| 21M64 | OTU01 | A08 | *Cerastium fontanum* | *Poa pratensis* | OTU13 | A08 | 21E48 | 8 | 17 | 10781 | 0.00074 | 0.00158 |
| 21M64 | OTU01 | A08 | *Cerastium fontanum* | *Deschampsia* | OTU05 | A04 | 21E05 | 53 | 215 | 91291 | 0.00058 | 0.00236 |
| 21M65 | OTU01 | A09 | *Cerastium fontanum* | *Cerastium fontanum* | OTU01 | A09 | 21E15 | 2 | 16 | 124241 | 0.00002 | 0.00013 |
| 21M65 | OTU01 | A09 | *Cerastium fontanum* | *Phleum alpinum* | OTU25 | A09 | 21E14 | 51 | 137 | 111946 | 0.00046 | 0.00122 |
| 21M66 | OTU01 | A11 | *Cerastium fontanum* | *Cerastium fontanum* | OTU01 | A11 | 21E17 | 1 | 12 | 148662 | 0.00001 | 0.00008 |
| 21M66 | OTU01 | A11 | *Cerastium fontanum* | *Cerastium fontanum* | OTU01 | A11 | 21F49 | 5 | 8 | 34572 | 0.00014 | 0.00023 |
| 21M66 | OTU01 | A11 | *Cerastium fontanum* | *Elymus* | OTU17 | A11 | 21E18 | 5 | 29 | 12131 | 0.00041 | 0.00239 |
| 21M66 | OTU01 | A11 | *Cerastium fontanum* | *Elymus* | OTU17 | A11 | 21F46 | 8 | 22 | 10408 | 0.00077 | 0.00211 |
| 21M67 | OTU01 | A11 | *Cerastium fontanum* | *Cerastium fontanum* | OTU01 | A11 | 21E17 | 6 | 16 | 148662 | 0.00004 | 0.00011 |
| 21M67 | OTU01 | A11 | *Cerastium fontanum* | *Cerastium fontanum* | OTU01 | A11 | 21F49 | 1 | 8 | 34572 | 0.00003 | 0.00023 |
| 21M67 | OTU01 | A11 | *Cerastium fontanum* | *Elymus* | OTU17 | A11 | 21E18 | 13 | 39 | 12131 | 0.00107 | 0.00321 |
| 21M67 | OTU01 | A11 | *Cerastium fontanum* | *Elymus* | OTU17 | A11 | 21F46 | 11 | 35 | 10408 | 0.00106 | 0.00336 |
| 21M68 | OTU01 | A11 | *Cerastium fontanum* | *Cerastium fontanum* | OTU01 | A11 | 21E17 | 4 | 12 | 148662 | 0.00003 | 0.00008 |
| 21M68 | OTU01 | A11 | *Cerastium fontanum* | *Cerastium fontanum* | OTU01 | A11 | 21F49 | 5 | 9 | 34572 | 0.00014 | 0.00026 |
| 21M68 | OTU01 | A11 | *Cerastium fontanum* | *Elymus* | OTU17 | A11 | 21E18 | 26 | 48 | 12131 | 0.00214 | 0.00396 |
| 21M68 | OTU01 | A11 | *Cerastium fontanum* | *Elymus* | OTU17 | A11 | 21F46 | 22 | 42 | 10408 | 0.00211 | 0.00404 |
| 21M69 | OTU01 | A11 | *Cerastium fontanum* | *Cerastium fontanum* | OTU01 | A11 | 21E17 | 3 | 10 | 148662 | 0.00002 | 0.00007 |
| 21M69 | OTU01 | A11 | *Cerastium fontanum* | *Cerastium fontanum* | OTU01 | A11 | 21F49 | 5 | 8 | 34572 | 0.00014 | 0.00023 |
| 21M69 | OTU01 | A11 | *Cerastium fontanum* | *Elymus* | OTU17 | A11 | 21E18 | 34 | 63 | 12131 | 0.00280 | 0.00519 |
| 21M69 | OTU01 | A11 | *Cerastium fontanum* | *Elymus* | OTU17 | A11 | 21F46 | 30 | 51 | 10408 | 0.00288 | 0.00490 |
| 21M70 | OTU04 | A20 | *Poa annua* | *Poa annua* | OTU04 | A20 | 21D45 | 5 | 11 | 51169 | 0.00010 | 0.00021 |
| 21M70 | OTU04 | A20 | *Poa annua* | *Poa annua* | OTU04 | A20 | 21F44 | 6 | 14 | 49937 | 0.00012 | 0.00028 |
| 21M71 | OTU04 | A20 | *Poa annua* | *Poa annua* | OTU04 | A20 | 21D45 | 4 | 48 | 51169 | 0.00008 | 0.00094 |
| 21M71 | OTU04 | A20 | *Poa annua* | *Poa annua* | OTU04 | A20 | 21F44 | 8 | 49 | 49937 | 0.00016 | 0.00098 |
| 21M72 | OTU01 | B01 | *Cerastium fontanum* | *Cerastium fontanum* | OTU01 | B01 | 21D56 | 2 | 2 | 43938 | 0.00005 | 0.00005 |
| 21M72 | OTU01 | B01 | *Cerastium fontanum* | *Cerastium fontanum* | OTU01 | B01 | 21F27 | 0 | 9 | 144784 | 0.00000 | 0.00006 |
| 21M72 | OTU01 | B01 | *Cerastium fontanum* | *Juncus scheuchzerioides* | OTU27 | B01 | 21F35 | 22 | 55 | 35935 | 0.00061 | 0.00153 |
| 21M73 | OTU01 | B01 | *Cerastium fontanum* | *Cerastium fontanum* | OTU01 | B01 | 21D56 | 1 | 2 | 43938 | 0.00002 | 0.00005 |
| 21M73 | OTU01 | B01 | *Cerastium fontanum* | *Cerastium fontanum* | OTU01 | B01 | 21F27 | 0 | 9 | 144784 | 0.00000 | 0.00006 |
| 21M73 | OTU01 | B01 | *Cerastium fontanum* | *Juncus scheuchzerioides* | OTU27 | B01 | 21F35 | 17 | 41 | 35935 | 0.00047 | 0.00114 |
| 21M74 | OTU01 | B01 | *Cerastium fontanum* | *Cerastium fontanum* | OTU01 | B01 | 21D56 | 2 | 9 | 43938 | 0.00005 | 0.00020 |
| 21M74 | OTU01 | B01 | *Cerastium fontanum* | *Cerastium fontanum* | OTU01 | B01 | 21F27 | 3 | 12 | 144784 | 0.00002 | 0.00008 |
| 21M74 | OTU01 | B01 | *Cerastium fontanum* | *Juncus scheuchzerioides* | OTU27 | B01 | 21F35 | 22 | 43 | 35935 | 0.00061 | 0.00120 |
| 21M75 | OTU01 | B01 | *Cerastium fontanum* | *Cerastium fontanum* | OTU01 | B01 | 21D56 | 1 | 4 | 43938 | 0.00002 | 0.00009 |
| 21M75 | OTU01 | B01 | *Cerastium fontanum* | *Cerastium fontanum* | OTU01 | B01 | 21F27 | 0 | 7 | 144784 | 0.00000 | 0.00005 |
| 21M75 | OTU01 | B01 | *Cerastium fontanum* | *Juncus scheuchzerioides* | OTU27 | B01 | 21F35 | 30 | 55 | 35935 | 0.00083 | 0.00153 |
| 21M76 | OTU01 | B01 | *Cerastium fontanum* | *Cerastium fontanum* | OTU01 | B01 | 21D56 | 1 | 1 | 43938 | 0.00002 | 0.00002 |
| 21M76 | OTU01 | B01 | *Cerastium fontanum* | *Cerastium fontanum* | OTU01 | B01 | 21F27 | 0 | 8 | 144784 | 0.00000 | 0.00006 |
| 21M76 | OTU01 | B01 | *Cerastium fontanum* | *Juncus scheuchzerioides* | OTU27 | B01 | 21F35 | 24 | 37 | 35935 | 0.00067 | 0.00103 |
| 21M77 | OTU01 | B01 | *Cerastium fontanum* | *Cerastium fontanum* | OTU01 | B01 | 21D56 | 0 | 1 | 43938 | 0.00000 | 0.00002 |
| 21M77 | OTU01 | B01 | *Cerastium fontanum* | *Cerastium fontanum* | OTU01 | B01 | 21F27 | 1 | 9 | 144784 | 0.00001 | 0.00006 |
| 21M77 | OTU01 | B01 | *Cerastium fontanum* | *Juncus scheuchzerioides* | OTU27 | B01 | 21F35 | 21 | 40 | 35935 | 0.00058 | 0.00111 |
| 21M78 | OTU40 | B03 | *Sagina procumbens* | *Cerastium fontanum* | OTU01 | B03 | 21E62 | 97 | 185 | 46855 | 0.00207 | 0.00395 |
| 21M78 | OTU40 | B03 | *Sagina procumbens* | *Cerastium fontanum* | OTU01 | B03 | 21E63 | 65 | 132 | 30976 | 0.00210 | 0.00426 |
| 21M78 | OTU40 | B03 | *Sagina procumbens* | *Poa annua* | OTU32 | B03 | 21F23 | 17 | 58 | 9829 | 0.00173 | 0.00590 |
| 21M78 | OTU40 | B03 | *Sagina procumbens* | *Sagina procumbens* | OTU40 | B03 | 21E64 | 2 | 3 | 8415 | 0.00024 | 0.00036 |
| 21M78 | OTU40 | B03 | *Sagina procumbens* | *Sagina procumbens* | OTU40 | B03 | 21F20 | 0 | 2 | 26746 | 0.00000 | 0.00007 |
| 21M78 | OTU40 | B03 | *Sagina procumbens* | *Sagina procumbens* | OTU40 | B03 | 21F21 | 1 | 3 | 58768 | 0.00002 | 0.00005 |
| 21M78 | OTU40 | B03 | *Sagina procumbens* | *Taraxacum officinale* | OTU42 | B03 | 21E60 | 74 | 160 | 119099 | 0.00062 | 0.00134 |
| 21M78 | OTU40 | B03 | *Sagina procumbens* | *Taraxacum officinale* | OTU42 | B03 | 21F19 | 77 | 141 | 124259 | 0.00062 | 0.00113 |
| 21M78 | OTU40 | B03 | *Sagina procumbens* | *Taraxacum officinale* | OTU42 | B03 | 21F22 | 94 | 199 | 151339 | 0.00062 | 0.00131 |
| 21M79 | OTU40 | B03 | *Sagina procumbens* | *Cerastium fontanum* | OTU01 | B03 | 21E62 | 63 | 173 | 46855 | 0.00134 | 0.00369 |
| 21M79 | OTU40 | B03 | *Sagina procumbens* | *Cerastium fontanum* | OTU01 | B03 | 21E63 | 63 | 145 | 30976 | 0.00203 | 0.00468 |
| 21M79 | OTU40 | B03 | *Sagina procumbens* | *Poa annua* | OTU32 | B03 | 21F23 | 21 | 44 | 9829 | 0.00214 | 0.00448 |
| 21M79 | OTU40 | B03 | *Sagina procumbens* | *Sagina procumbens* | OTU40 | B03 | 21E64 | 3 | 3 | 8415 | 0.00036 | 0.00036 |
| 21M79 | OTU40 | B03 | *Sagina procumbens* | *Sagina procumbens* | OTU40 | B03 | 21F20 | 2 | 2 | 26746 | 0.00007 | 0.00007 |
| 21M79 | OTU40 | B03 | *Sagina procumbens* | *Sagina procumbens* | OTU40 | B03 | 21F21 | 3 | 4 | 58768 | 0.00005 | 0.00007 |
| 21M79 | OTU40 | B03 | *Sagina procumbens* | *Taraxacum officinale* | OTU42 | B03 | 21E60 | 101 | 200 | 119099 | 0.00085 | 0.00168 |
| 21M79 | OTU40 | B03 | *Sagina procumbens* | *Taraxacum officinale* | OTU42 | B03 | 21F19 | 127 | 150 | 124259 | 0.00102 | 0.00121 |
| 21M79 | OTU40 | B03 | *Sagina procumbens* | *Taraxacum officinale* | OTU42 | B03 | 21F22 | 150 | 202 | 151339 | 0.00099 | 0.00133 |
| 21M80 | OTU40 | B03 | *Sagina procumbens* | *Cerastium fontanum* | OTU01 | B03 | 21E62 | 49 | 111 | 46855 | 0.00105 | 0.00237 |
| 21M80 | OTU40 | B03 | *Sagina procumbens* | *Cerastium fontanum* | OTU01 | B03 | 21E63 | 27 | 73 | 30976 | 0.00087 | 0.00236 |
| 21M80 | OTU40 | B03 | *Sagina procumbens* | *Poa annua* | OTU32 | B03 | 21F23 | 9 | 31 | 9829 | 0.00092 | 0.00315 |
| 21M80 | OTU40 | B03 | *Sagina procumbens* | *Sagina procumbens* | OTU40 | B03 | 21E64 | 2 | 3 | 8415 | 0.00024 | 0.00036 |
| 21M80 | OTU40 | B03 | *Sagina procumbens* | *Sagina procumbens* | OTU40 | B03 | 21F20 | 0 | 2 | 26746 | 0.00000 | 0.00007 |
| 21M80 | OTU40 | B03 | *Sagina procumbens* | *Sagina procumbens* | OTU40 | B03 | 21F21 | 1 | 4 | 58768 | 0.00002 | 0.00007 |
| 21M80 | OTU40 | B03 | *Sagina procumbens* | *Taraxacum officinale* | OTU42 | B03 | 21E60 | 76 | 174 | 119099 | 0.00064 | 0.00146 |
| 21M80 | OTU40 | B03 | *Sagina procumbens* | *Taraxacum officinale* | OTU42 | B03 | 21F19 | 76 | 154 | 124259 | 0.00061 | 0.00124 |
| 21M80 | OTU40 | B03 | *Sagina procumbens* | *Taraxacum officinale* | OTU42 | B03 | 21F22 | 91 | 184 | 151339 | 0.00060 | 0.00122 |
| 21M81 | OTU28 | B04 | *Juncus scheuchzerioides* | *Juncus scheuchzerioides* | OTU28 | B04 | 21E65 | 37 | 70 | 97980 | 0.00038 | 0.00071 |
| 21M81 | OTU28 | B04 | *Juncus scheuchzerioides* | *Juncus scheuchzerioides* | OTU28 | B04 | 21F33 | 34 | 54 | 93054 | 0.00037 | 0.00058 |
| 21M81 | OTU28 | B04 | *Juncus scheuchzerioides* | *Juncus scheuchzerioides* | OTU28 | B04 | 21F34 | 30 | 71 | 92994 | 0.00032 | 0.00076 |
| 21M82 | OTU28 | B04 | *Juncus scheuchzerioides* | *Juncus scheuchzerioides* | OTU28 | B04 | 21E65 | 39 | 39 | 97980 | 0.00040 | 0.00040 |
| 21M82 | OTU28 | B04 | *Juncus scheuchzerioides* | *Juncus scheuchzerioides* | OTU28 | B04 | 21F33 | 35 | 39 | 93054 | 0.00038 | 0.00042 |
| 21M82 | OTU28 | B04 | *Juncus scheuchzerioides* | *Juncus scheuchzerioides* | OTU28 | B04 | 21F34 | 37 | 69 | 92994 | 0.00040 | 0.00074 |
| 21M83 | OTU28 | B04 | *Juncus scheuchzerioides* | *Juncus scheuchzerioides* | OTU28 | B04 | 21E65 | 38 | 57 | 97980 | 0.00039 | 0.00058 |
| 21M83 | OTU28 | B04 | *Juncus scheuchzerioides* | *Juncus scheuchzerioides* | OTU28 | B04 | 21F33 | 36 | 58 | 93054 | 0.00039 | 0.00062 |
| 21M83 | OTU28 | B04 | *Juncus scheuchzerioides* | *Juncus scheuchzerioides* | OTU28 | B04 | 21F34 | 37 | 66 | 92994 | 0.00040 | 0.00071 |
| 21M84 | OTU28 | B04 | *Juncus scheuchzerioides* | *Juncus scheuchzerioides* | OTU28 | B04 | 21E65 | 29 | 47 | 97980 | 0.00030 | 0.00048 |
| 21M84 | OTU28 | B04 | *Juncus scheuchzerioides* | *Juncus scheuchzerioides* | OTU28 | B04 | 21F33 | 37 | 58 | 93054 | 0.00040 | 0.00062 |
| 21M84 | OTU28 | B04 | *Juncus scheuchzerioides* | *Juncus scheuchzerioides* | OTU28 | B04 | 21F34 | 30 | 64 | 92994 | 0.00032 | 0.00069 |
| 21M85 | OTU01 | B08 | *Cerastium fontanum* | *Cerastium fontanum* | OTU01 | B08 | 21E79 | 0 | 5 | 57484 | 0.00000 | 0.00009 |
| 21M85 | OTU01 | B08 | *Cerastium fontanum* | *Colobanthus* | OTU40 | B08 | 21E52 | 4 | 19 | 8425 | 0.00047 | 0.00226 |
| 21M85 | OTU01 | B08 | *Cerastium fontanum* | *Juncus scheuchzerioides* | OTU29 | B08 | 21E53 | 108 | 253 | 92502 | 0.00117 | 0.00274 |
| 21M85 | OTU01 | B08 | *Cerastium fontanum* | *Juncus scheuchzerioides* | OTU29 | B08 | 21E54 | 84 | 203 | 79388 | 0.00106 | 0.00256 |
| 21M85 | OTU01 | B08 | *Cerastium fontanum* | *Poa annua* | OTU08 | B08 | 21E77 | 46 | 77 | 21484 | 0.00214 | 0.00358 |
| 21M85 | OTU01 | B08 | *Cerastium fontanum* | *Poa annua* | OTU08 | B08 | 21E78 | 27 | 46 | 12281 | 0.00220 | 0.00375 |
| 21M86 | OTU01 | C07 | *Cerastium fontanum* | *Cerastium fontanum* | OTU01 | C07 | 21F06 | 6 | 16 | 148503 | 0.00004 | 0.00011 |
| 21M86 | OTU01 | C07 | *Cerastium fontanum* | *Cerastium fontanum* | OTU01 | C07 | 21F08 | 0 | 7 | 54831 | 0.00000 | 0.00013 |
| 21M86 | OTU01 | C07 | *Cerastium fontanum* | *Phleum alpinum* | OTU47 | C07 | 21F18 | 12 | 55 | 19315 | 0.00062 | 0.00285 |
| 21M87 | OTU01 | C07 | *Cerastium fontanum* | *Cerastium fontanum* | OTU01 | C07 | 21F06 | 11 | 19 | 148503 | 0.00007 | 0.00013 |
| 21M87 | OTU01 | C07 | *Cerastium fontanum* | *Cerastium fontanum* | OTU01 | C07 | 21F08 | 0 | 7 | 54831 | 0.00000 | 0.00013 |
| 21M87 | OTU01 | C07 | *Cerastium fontanum* | *Phleum alpinum* | OTU47 | C07 | 21F18 | 31 | 58 | 19315 | 0.00160 | 0.00300 |
| 21M88 |  |  | CONTROL1 | *Taraxacum officinale* |  |  | 21E07 | 3415 | 3635 | 108195 | 0.03156 | 0.03360 |
| 21M88 |  |  | CONTROL1 | *Phleum alpinum* |  |  | 21E23 | 3798 | 4299 | 96370 | 0.03941 | 0.04461 |
| 21M88 |  |  | CONTROL1 | *Acaena magellanica* |  |  | 21E41 | 1015 | 1146 | 25540 | 0.03974 | 0.04487 |
| 21M88 |  |  | CONTROL1 | *Poa annua* |  |  | 21E33 | 3703 | 4251 | 99269 | 0.03730 | 0.04282 |
| 21M89 |  |  | CONTROL2 | *Taraxacum officinale* |  |  | 21E07 | 4941 | 5193 | 108195 | 0.04567 | 0.04800 |
| 21M89 |  |  | CONTROL2 | *Phleum alpinum* |  |  | 21E23 | 2260 | 2449 | 96370 | 0.02345 | 0.02541 |
| 21M89 |  |  | CONTROL2 | *Acaena magellanica* |  |  | 21E41 | 1119 | 1135 | 25540 | 0.04381 | 0.04444 |
| 21M89 |  |  | CONTROL2 | *Poa annua* |  |  | 21E33 | 2136 | 2450 | 99269 | 0.02152 | 0.02468 |
| 21M90 |  |  | CONTROL3 | *Taraxacum officinale* |  |  | 21E07 | 6003 | 7520 | 108195 | 0.05548 | 0.06950 |
| 21M90 |  |  | CONTROL3 | *Phleum alpinum* |  |  | 21E23 | 1438 | 4516 | 96370 | 0.01492 | 0.04686 |
| 21M90 |  |  | CONTROL3 | *Acaena magellanica* |  |  | 21E41 | 1526 | 1962 | 25540 | 0.05975 | 0.07682 |
| 21M90 |  |  | CONTROL3 | *Poa annua* |  |  | 21E33 | 3548 | 4235 | 99269 | 0.03574 | 0.04266 |
| 21M91 |  |  | CONTROL4 | *Taraxacum officinale* |  |  | 21E07 | 599 | 3376 | 108195 | 0.00554 | 0.03120 |
| 21M91 |  |  | CONTROL4 | *Phleum alpinum* |  |  | 21E23 | 250 | 883 | 96370 | 0.00259 | 0.00916 |
| 21M91 |  |  | CONTROL4 | *Acaena magellanica* |  |  | 21E41 | 172 | 883 | 25540 | 0.00673 | 0.03457 |
| 21M91 |  |  | CONTROL4 | *Poa annua* |  |  | 21E33 | 83 | 1024 | 99269 | 0.00084 | 0.01032 |
| 21M94 | OTU07 | A13 | *Acaena magellanica* | *Taraxacum officinale* |  |  | 21E07 | 63 | 185 | 108195 | 0.00058 | 0.00171 |
| 21M94 | OTU07 | A13 | *Acaena magellanica* | *Phleum alpinum* |  |  | 21E23 | 64 | 242 | 96370 | 0.00066 | 0.00251 |
| 21M94 | OTU07 | A13 | *Acaena magellanica* | *Acaena magellanica* |  |  | 21E41 | 0 | 0 | 25540 | 0.00000 | 0.00000 |
| 21M94 | OTU07 | A13 | *Acaena magellanica* | *Poa annua* |  |  | 21E33 | 78 | 256 | 99269 | 0.00079 | 0.00258 |
| 21M95 | OTU16 | A05 | *Taraxacum officinale* | *Taraxacum officinale* |  |  | 21E07 | 0 | 79 | 108195 | 0.00000 | 0.00073 |
| 21M95 | OTU16 | A05 | *Taraxacum officinale* | *Phleum alpinum* |  |  | 21E23 | 69 | 214 | 96370 | 0.00072 | 0.00222 |
| 21M95 | OTU16 | A05 | *Taraxacum officinale* | *Acaena magellanica* |  |  | 21E41 | 57 | 97 | 25540 | 0.00223 | 0.00380 |
| 21M95 | OTU16 | A05 | *Taraxacum officinale* | *Poa annua* |  |  | 21E33 | 69 | 211 | 99269 | 0.00070 | 0.00213 |
| 21M96 | OTU18 | A15 | *Phleum alpinum* | *Taraxacum officinale* |  |  | 21E07 | 119 | 286 | 108195 | 0.00110 | 0.00264 |
| 21M96 | OTU18 | A15 | *Phleum alpinum* | *Phleum alpinum* |  |  | 21E23 | 8 | 99 | 96370 | 0.00008 | 0.00103 |
| 21M96 | OTU18 | A15 | *Phleum alpinum* | *Acaena magellanica* |  |  | 21E41 | 78 | 120 | 25540 | 0.00305 | 0.00470 |
| 21M96 | OTU18 | A15 | *Phleum alpinum* | *Poa annua* |  |  | 21E33 | 74 | 248 | 99269 | 0.00075 | 0.00250 |
